# Understanding fibrosis pathogenesis via modeling macrophage-fibroblast interplay in immune-metabolic context

**DOI:** 10.1038/s41467-022-34241-5

**Published:** 2022-10-30

**Authors:** Elisa Setten, Alessandra Castagna, Josué Manik Nava-Sedeño, Jonathan Weber, Roberta Carriero, Andreas Reppas, Valery Volk, Jessica Schmitz, Wilfried Gwinner, Haralampos Hatzikirou, Friedrich Feuerhake, Massimo Locati

**Affiliations:** 1 IRCCS Humanitas Research Hospital, Rozzano, Italy; 2 Department of Medical Biotechnologies and Translational Medicine, Università degli Studi di Milano, Milan, Italy; 3 Mathematics Department, National Autonomous University of Mexico, Mexico City, Mexico; 4 IRIMAS Institute, Université de Haute-Alsace, Mulhouse, France; 5 Charité-Universitätsmedizin Berlin, corporate member of Freie Universität Berlin, Humboldt-Universität, Berlin, Germany; 6 Institute for Pathology, Hannover Medical School, Hannover, Germany; 7 Department of Nephrology, Hannover Medical School, Hannover, Germany; 8 Mathematics Department, Khalifa University, PO Box 127788, Abu Dhabi, UAE; 9 Technische Univesität Dresden, Center for Information Services and High Performance Computing, Nöthnitzer Straße 46, 01062 Dresden, Germany; 10 Healthcare Engineering Innovation Center (HEIC), Khalifa University of Science and Technology, Abu Dhabi, UAE; 11 Institute for Neuropathology, University Clinic Freiburg, Freiburg, Germany; 12 Present Address: Department of Oncology, University of Torino, Torino, Italy; 13 Present Address: Candiolo Cancer Institute-IRCCS-FPO, Candiolo, Italy; 14 Present Address: Technical Research and Development, GSK, 1 Via Fiorentina, 53100 Siena, SI Italy

**Keywords:** Systems biology, Computational models, Renal fibrosis, Monocytes and macrophages

## Abstract

Fibrosis is a progressive biological condition, leading to organ dysfunction in various clinical settings. Although fibroblasts and macrophages are known as key cellular players for fibrosis development, a comprehensive functional model that considers their interaction in the metabolic/immunologic context of fibrotic tissue has not been set up. Here we show, by transcriptome-based mathematical modeling in an in vitro system that represents macrophage-fibroblast interplay and reflects the functional effects of inflammation, hypoxia and the adaptive immune context, that irreversible fibrosis development is associated with specific combinations of metabolic and inflammatory cues. The in vitro signatures are in good alignment with transcriptomic profiles generated on laser captured glomeruli and cortical tubule-interstitial area, isolated from human transplanted kidneys with advanced stages of glomerulosclerosis and interstitial fibrosis/tubular atrophy, two clinically relevant conditions associated with organ failure in renal allografts. The model we describe here is validated on tissue based quantitative immune-phenotyping of biopsies from transplanted kidneys, demonstrating its feasibility. We conclude that the combination of in vitro and in silico modeling represents a powerful systems medicine approach to dissect fibrosis pathogenesis, applicable to specific pathological conditions, and develop coordinated targeted approaches.

## Abstract (web summary)

Renal fibrosis is a progressive process with complex etiopathology, causing organ failure. Here authors present a mathematical model, based on an in vitro system faithfully contemplating macrophage-fibroblast interaction and the metabolic-immunologic signals that are affecting kidney fibrosis, that is applicable to kidney transplant failure.

## Introduction

Fibrosis is the final state of continuous scarring that occurs normally during healing processes but also significantly contributes reducing organ function in several chronic diseases^1^. Four core mechanisms are involved: (i) early inflammatory events with involvement of various immune cells, including T cells and macrophages (Mφ); (ii) activation of fibroblasts (Fb) to myofibroblasts (collagen-secreting α-SMA^+^activated Fb) and extracellular matrix (ECM) deposition, generating interstitial scars; (iii) loss of epithelial cells regenerative properties; (iv) loss of interstitial capillary integrity, compromising oxygen delivery and leading to a cascade of events related to hypoxia-oxidant stress, further promoting the fibrotic process^2^. Chemokines and profibrotic cytokines tightly control recruitment and activation of inflammatory cells at the site of injury. In a tissue microenvironment dominated by Th2 cytokines (IL-4, IL-13), Mφ enhance secretion and activation of latent TGFβ1, Fb proliferation and ECM production, conversion of epithelial cells into collagen-producing myofibroblasts via epithelial-mesenchymal transition, and release of proangiogenic factors to promote vascular remodeling and angiogenesis^3–5^. Pathologic angiogenesis and vessel sprouting in hypoxic tissues worsen fibrosis by leading to continuous myofibroblast activation and proliferation^6–9^.

Fibrosis characterizes the progression of chronic diseases occurring in many different tissues, including skin, lung, liver, heart, and kidney^1^. In chronic kidney diseases, and in particular, after renal transplantation, fibrosis can result in glomerulosclerosis with loss of glomerular filter capacity and interstitial fibrosis and tubular atrophy (IF/TA), which is associated with impaired tubular adsorption and secretion processes and regulatory functions and declining renal function over time^10^. Accumulation of ECM components in glomeruli and in the cortical interstitium collectively results in progressive loss of renal function^11^. The role of Mφ in acute rejection of kidney transplant, through their contribution to both T cell-mediated and antibody-mediated rejection processes, is well established^12,13^. On the opposite, their role in IF/TA development is still under debate, since positive and negative effects of Mφ infiltration on long-term renal allograft functions were reported^12,14–16^. In IF/TA, Mφ have been shown to switch from a classical proinflammatory phenotype (also alluded to as M1) to an alternative phenotype (also alluded to as M2) typically associated with progression of fibrosis^17,18^ prompted by the release of profibrotic factors, such as TGFβ1, FGF2 and PDGF, that promote myofibroblasts proliferation and activation, leading to ECM overproduction^19^. However, in different models, chronic inflammation and subsequent fibrosis have been shown to be promoted by depletion of Mφ in certain stages of the disease, and in several diseases an increase in M2 Mφ characterizes the recovery phase^5,20–22^.

Mathematical modeling in biology has been proven an invaluable tool in investigating existing biological hypotheses in realistic scenarios or generating experimentally testable ones^23,24^. Based on the in vitro analysis of Fb and Mφ interplay in the context of wound healing and scarring processes a mathematical model of fibrosis has been recently developed, which under parameter variations robustly predicted three functional states: a state of healing associated with modest ECM production, and two fibrotic states associated with excessive ECM production and different cellularity where a prominent myofibroblast infiltration is associated with high or low numbers of Mφ, termed “hot” and “cold” fibrosis, respectively^25^. The model comprehensively described central aspects of fibrosis and potentially predicted cell networks leading to clinically observed conditions, but was limited by the fact that Mφ/Fb interactions always occur in the context of specific immunological and metabolic features characterizing the tissue microenvironment^26^, an aspect that has not yet been modeled. Here we develop a mathematical model by translating in vitro observations, on the relative relevance of different immune and metabolic microenvironmental cues to the Mφ/Fb interactions, to an in vivo fibrotic scenario related to IF/TA progression and glomerulosclerosis in transplanted kidneys (research design is shown in Fig. S1a). We consider fibrosis after transplantation as a clinically relevant application case for the model. Large scale evaluation of immune cell populations in renal biopsies^27,28^ showed some potential for prognosis^14–16^ but was insufficient to predict fibrosis and functional decline^29^.

Starting from an in vitro setting that allows transcriptomic dissection of the effects of different microenvironmental cues on the macrophage/fibroblast interplay occurring during fibrosis development, we instruct a mathematical model that predicts key interactions for fibrosis development and apply it to tissue sections from human transplanted kidneys with different degree of glomerulosclerosis and IF/TA. We propose an integrated approach based on a combination of biological and mathematical models that allow the implementation of immune and metabolic cues in the current “hot” and “cold” fibrosis model. By demonstrating the feasibility of this model on transplanted kidney biopsies, we provide an innovative approach to analyze fibrosis pathogenesis and guide the development of targeted therapies.

## Results

### Global transcriptomes associated with Mφ and Fb activation and their cell-cell interactions

**Fig. 1:**
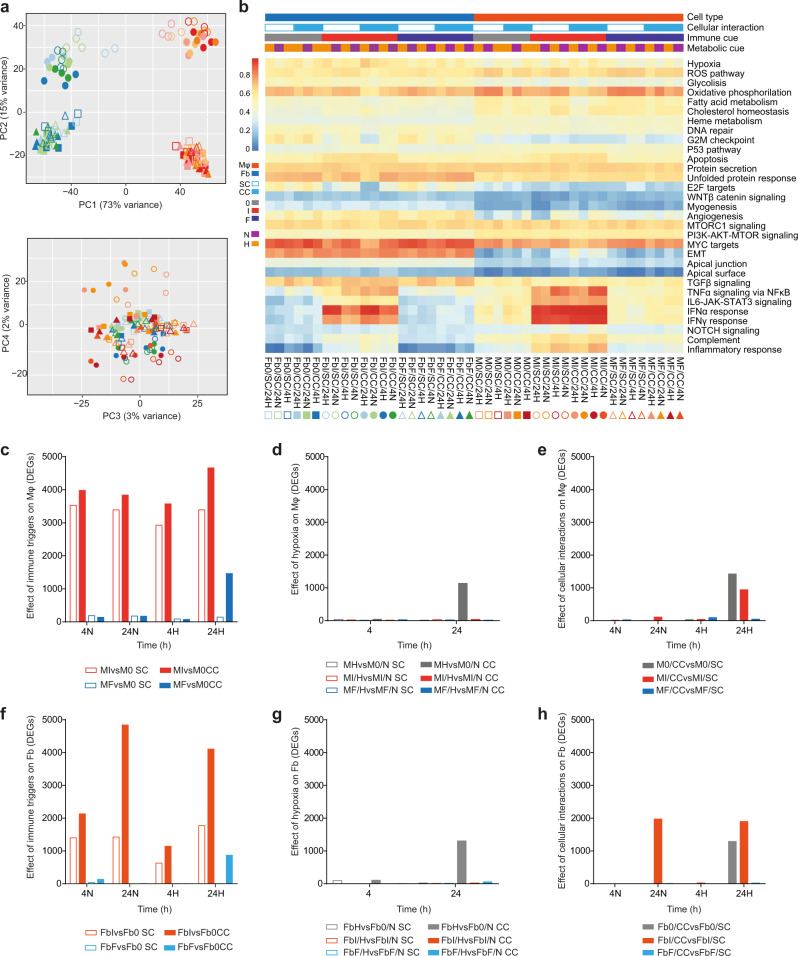
Global transcriptome analysis of macrophages (Mφ) and fibroblasts (Fb) exposed to distinct immune, metabolic and culture conditions. **a** PCA on all samples indicates that the 2 components describing most of the system variability are represented by the cell type and inflammation (73% - PC1 and 15% - PC2, respectively, in the upper panel). Other sources of variability are not detectable (see PC3 and PC4 in the bottom panel). For sample codes refer to Fig. 1b. **b** Single sample GSEA (ssGSEA) on the hallmark database of enriched gene ontologies. Labels indicate 44 sample conditions (columns) and hallmark categories enriched (rows). Samples are grouped by cell type (Mφ or Fb), cellular interaction (CC or SC), immune cues (0 or I or F) and metabolic cue (H or N; Mφ and Fb in different shades of orange or blue, respectively). Different shape and color indicate different sample conditions: squares = resting (0), circles = proinflammatory (I) and triangles = profibrotic (F); empty and full shapes are related to single culture (SC) and coculture (CC), respectively; different shade of color are related to time (4 or 24 h) and oxygen status (N = normoxia, H = hypoxia). **c**–**h** Quantification of differentially expressed genes (DEG; FDR ≤ 0.05) in Mφ and Fb depending on immune cues, hypoxia, or cellular interaction. **c** reports the effect of immune cues comparing proinflammatory or profibrotic Mφ to resting Mφ (MIvsM0 = red bars, MFvsM0 = blue bars, respectively) in SC or CC (empty and full bars, respectively) at 4 or 24 h of normoxia (4 N or 24 N) or hypoxia (4H or 24H). **d** reports the effect of hypoxia on Mφ comparing hypoxic to normoxic Mφ in resting (MHvsM0/N = gray bars), proinflammatory (MI/HvsMI/N = red bars) and profibrotic (MF/HvsMF/N = blue bars) conditions, in SC or CC (empty and full bars, respectively), both at 4 or 24 h. **e** reports the effect of Fb on Mφ comparing cocultivated to single cultivated Mφ in resting (M0/CCvsM0/SC = gray bars), proinflammatory (MI/CCvsMI/SC = red bars) and profibrotic (MF/CCvsMF/SC = blue bars) conditions at 4 or 24 h of normoxia (4 N or 24 N) or hypoxia (4H or 24H). **f**–**h** report the same comparisons of **c**–**e**, respectively, but performed on Fb. Source data of **c**–**h** are provided as a Source Data file.

Transcriptional events related to the interaction of Mφ and Fb in the context of defined immune and metabolic settings were investigated by RNAseq (see Fig. S1b for experimental design and sample coding of the in vitro approach). Principal component analysis (PCA) of transcriptomes associated with the 44 experimental conditions revealed that the most relevant sources of variability in the system were the cell types and their response to inflammatory conditions (PC1 and PC2, respectively, collectively accounting for 88% of total variance; Fig. 1a). Biological hallmarks were investigated by single-sample Gene Set Enrichment Analysis (ssGSEA), revealing that hypoxia, the reactive oxygen species (ROS) pathway, glycolysis, oxidative phosphorylation, fatty acid metabolism, cholesterol homeostasis, and TGFβ signaling were the major drivers of differential gene expression across of the entire dataset. In both Mφ and Fb, proinflammatory conditions were associated with IFN- and TNF-related pathways, whereas genes related to epithelial-mesenchymal transition and angiogenesis were enriched in Fb but not in Mφ (Fig. 1b).

The contribution of the distinct parameters was then investigated by multi-level analysis to define the impact of each single variable (1st level analysis) and the combinatorial effects of two (2nd level analysis) or three of them (3rd level analysis). The differentially expressed genes (DEG), selected as described in Methods section, were then clustered based on the main variable taken into account. When the analysis was focused on the relevance of the immune network, inflammatory/Th1 conditions, mimicked by exposing cells to the combined effect of the proinflammatory mediator lipopolysaccharide (LPS) plus the type 1 cytokine IFNγ, emerged as a major driver for both cell types, generating a high number of DEGs in both Mφ and Fb. On the opposite, profibrotic/Th2 conditions, mimicked by exposing cells to the type 2 cytokine IL-4, showed a divergent effect between Mφ, which regulated hundreds of DEGs, and Fb, which did not significantly differ from their untreated counterpart (Fig. 1c, f for Mφ and Fb, respectively). The relevance of taking into consideration the combined effects of multiple parameters was exemplified by the analysis on hypoxia, whose effects in both cell types were significantly enhanced when cells were activated in coculture conditions as compared to single cell cultures (Fig. 1d, g for Mφ and Fb, respectively). Similarly, Mφ and particularly Fb exposed to proinflammatory conditions regulated hundreds of additional DEGs specifically when activated in the presence of the second cell type (Fig. 1e, h for Mφ and Fb, respectively).

### Effects of inflammation on Fb and Mφ are highly influenced by the tissue microenvironment

**Fig. 2:**
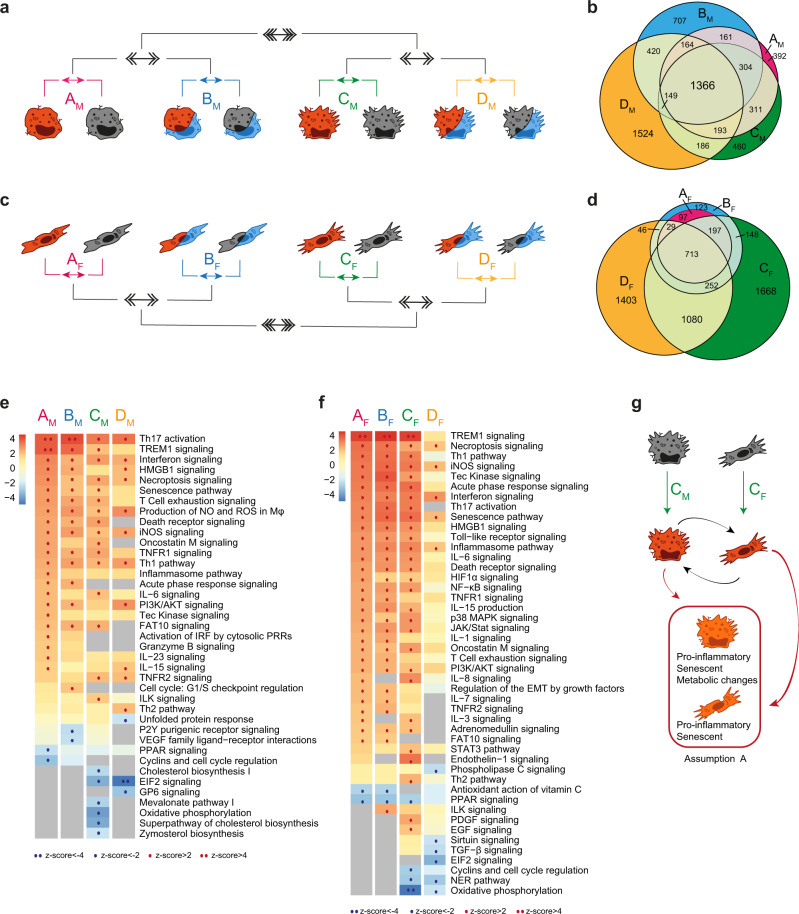
Role of inflammation on the global biological response. **a**, **c** Schematic representation of the multi-level approach to study the effect of inflammation on Mφ (**a**, **b**, **e**) and Fb (**c**, **d**, **f**) in different metabolic and culture conditions. 1st level compares LPS+IFNγ stimulated cells to resting cells in four different conditions: normoxic and single culture (comparison A_M_/A_F_ in pink), hypoxic and single culture (B_M_/B_F_ in blue), normoxic and coculture (C_M_/C_F_ in green), hypoxic and coculture (D_M_/D_F_ in yellow). 2nd level (double arrows) compares hypoxic to normoxic treatment (AvsB and CvsD), 3rd level (triple arrows) compares coculture to single culture conditions [(A+B)vs(C+D)]. **b**, **d** Venn diagrams show the distribution of DEGs in each 1st level comparison in Mφ (**b**) and Fb (**d**). **e**, **f** Pathways enrichment analysis in Mφ (**e**) and Fb (**f**) comparisons. Columns represent 1st level of comparisons (A-B-C-D), rows report pathways significantly modulated (|z-score | ≥ 2) in at least one comparison. Color intensity bar indicates the level of positive (in red) or negative (in blue) enrichment; dots appear only when pathways are significantly enriched; gray indicates no modulation. See also Fig. S2. **g** Schematic representation of cellular changes in the key condition which feeds assumption A in the mathematical model. Source data of heatmaps **e**, **f** are provided as a Source Data file.

As inflammation is a potent immune driver, able to induce phenotypic and functional changes in different cell types^30,31^, we first focused our analysis on its functional effects on Mφ and Fb (Fig. 2a, c, respectively) comparing LPS + IFNγ-stimulated Mφ (MI) and Fb (FbI) with resting counterparts in single culture/normoxic conditions (comparisons A_M_ and A_F_), in single culture/hypoxic conditions (comparisons B_M_ and B_F_), in coculture/normoxic conditions (comparisons C_M_ and C_F_), and in coculture/hypoxic conditions (comparisons D_M_ and D_F_). As expected, this analysis revealed that both Mφ and Fb acquired a proinflammatory phenotype across different metabolic/culture conditions when exposed to an inflammatory setting (Fig. S2 and Supplementary Data 1). Keeping the focus on the role of inflammation, we then applied a 2nd level analysis comparing hypoxic versus normoxic conditions in single culture (comparison AvsB) and in coculture (comparison CvsD) conditions, and then a 3rd level analysis comparing single culture and coculture conditions (comparison [(AvsB)vs(CvsD)]). This analysis revealed that a high amount of DEGs was shared among all Mφ comparisons, with the remarkable exception of D_M_, which was characterized by the emergence of 1524 specific DEGs, revealing that even if inflammation per se is a major Mφ activator the concomitant presence of Fb and hypoxic conditions adds further complexity to its functional effects on Mφ (Fig. 2b). In line with the robustness of inflammation as Mφ-activating driver, pathway enrichment analysis revealed that in Mφ most functional pathways were linked to inflammation, adaptive immune response and cell senescence, and were shared through different experimental conditions (Fig. 2e). A remarkable exception was represented by the negative regulation of a set of functional pathways related to cholesterol metabolism in Mφ inflamed in the presence of Fb (comparison C_M_ in Fig. 2e).

Similar to Mφ, inflammatory cues induced also in Fb a high number of DEGs sustaining functional pathways clearly associated with fibrosis and cell senescence (Fig. 2d, f). Unexpectedly, however, most of these DEGs were significantly modulated only in Fb cocultivated with Mφ (comparisons C_F_ and/or D_F_ in Fig. 2f). Focusing on the gene enrichment analysis in Fb in coculture with Mφ, a lower activation of several pathways related to inflammation and fibrosis was evident in the concomitant presence of hypoxia (comparison D_F_ in Fig. 2f), while Mφ per se had a significant impact on pathways related to cell cycle regulation and metabolism (comparison C_F_ in Fig. 2f). These findings indicate the emergence of a senescent phenotype of both Mφ and Fb in inflamed tissues (here mimicked by coculture conditions), with Mφ modifying their metabolic profile and Fb regulating their cell proliferation properties, and further subtle metabolic adjustments related to the concomitant presence of hypoxia. These phenotypic changes lay the ground for assumption A of our mathematical model (Fig. 2g).

### Type 2 immune responses have direct effect on Mφ, which then indirectly affect Fb

**Fig. 3:**
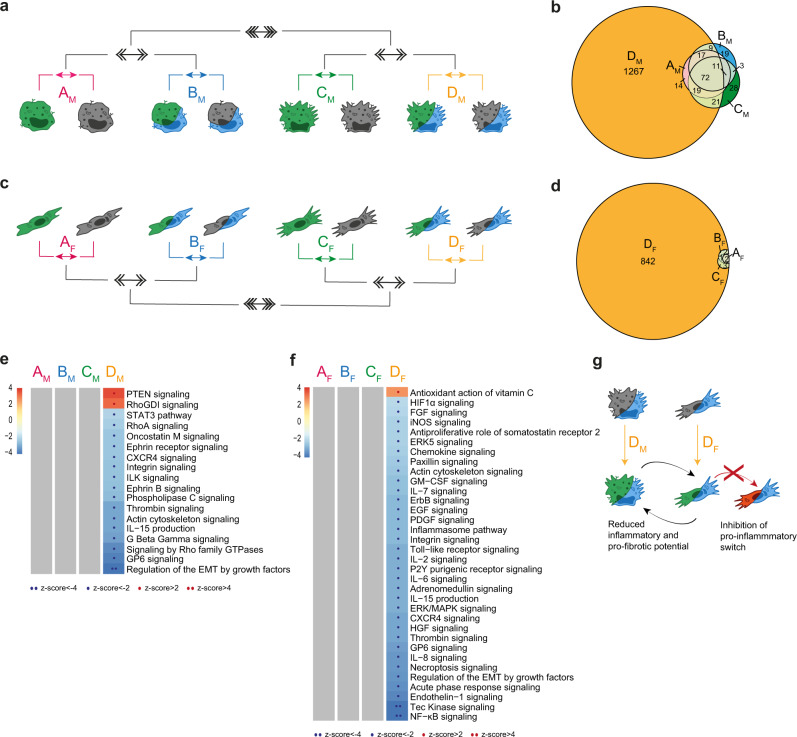
Role of adaptive Th2 immune response (IL-4) on the global biological response. **a**, **c** Schematic representation of the multi-level approach to study the effect of Th2 cytokine on Mφ (**a**, **b**, **e**) and Fb (**c**, **d**, **f**) in different metabolic and culture conditions. 1st level compares IL-4 stimulated cells to resting cells in four different conditions: normoxic and single culture (comparison A_M_/A_F_ in pink), hypoxic and single culture (B_M_/B_F_ in blue), normoxic and coculture (C_M_/C_F_ in green), hypoxic and coculture (D_M_/D_F_ in yellow). 2nd level (double arrows) compares hypoxic to normoxic treatment (AvsB and CvsD) and 3rd level (triple arrows) compares coculture to single culture conditions [(A+B)vs(C+D)]. **b**, **d** Venn diagrams with DEGs distribution in each 1st level comparison in Mφ (**b**) and Fb (**d**). **e**, **f** Pathways enrichment analysis in Mφ (**e**) and Fb (**f**) comparisons. Columns represent 1st level comparisons (A-B-C-D), rows report pathways significantly modulated (|z-score | ≥2) in at least one comparison. Bar color intensity indicates the level of positive (red) or negative (blue) enrichment; dots appear only when pathways are significantly enriched; gray indicates no modulation. See also Fig. S3. **g** Schematic representation of cellular changes in the condition with higher number of DEGs. Source data of heatmaps **e**, **f** are provided as a Source Data file.

During fibrosis development, adaptive immune responses in the site of lesion influence both immune and non-immune cells. Th2 cytokines in particular induce in Mφ a phenotypic switching into an alternative phenotype (M2, here indicated as MF), while on Fb contribute to promoting their activation into myofibroblasts involved in ECM components production (here indicated as FbF)^32^. As expected, IL-4 (here used to mimic the contribution of the Th2 immune pathway) induced a restricted but well-defined transcriptional profile in Mφ, which was largely insensitive to the hypoxic context or the concomitant presence of Fb (Fig. 3b and comparisons A_M_, B_M_, and C_M_ in Fig. S3). On the opposite, IL-4 had no direct effect on Fb, nor was it able to induce Fb activation when hypoxia and Mφ were added as single variables (Fig. 3d and comparisons A_F_, B_F_, and C_F_ in Fig. S3 and Supplementary Data 2). However, a dramatic change in the transcriptional profile of both Mφ and Fb was observed when IL-4 was applied to both cell types under hypoxic conditions (Fig. 3b, d). Specifically, when cocultivated in hypoxia and in the presence of Fb, IL-4-conditioned Mφ showed a negative regulation of pathways linked to ECM deposition. A similar effect was observed in IL-4-conditioned Fb, which also showed a negative regulation of inflammation- and cell growth-related pathways specifically when exposed to hypoxia and in the concomitant presence of Mφ. Under these experimental conditions, both cell types also showed a significant regulation of functional pathways involved in cell-cell contacts, indicating that a direct Mφ/Fb interaction may be involved. Thus, at least in this in vitro setting, the development of a Th2 immune response per se has distinct effects on Mφ and negligible effects on Fb, but when hypoxic conditions are imposed to the tissue (i.e., Mφ and Fb are allowed to directly interact with each other) IL-4 profoundly influences both inflammatory and profibrotic potential of both cell types (Fig. 3g).

### Hypoxia effects are insensitive to the specific immune microenvironment but critical to cell-cell interactions

**Fig. 4:**
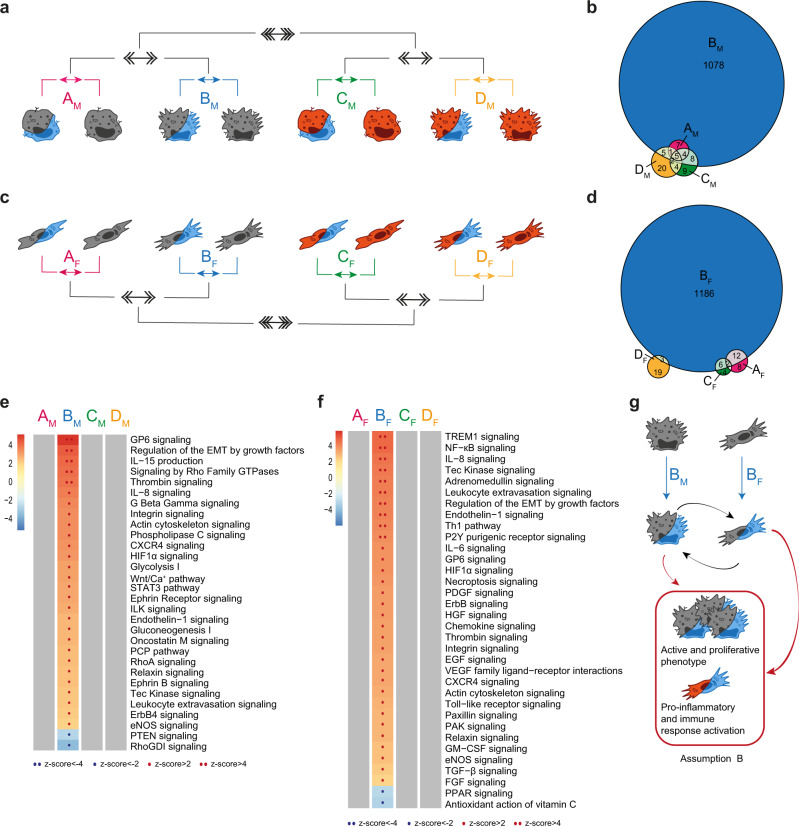
Role of hypoxia on the global biological response. **a**, **c** Schematic representation of the multi-level approach to study the effect of hypoxia on Mφ (**a**, **b**, **e**) and Fb (**c**, **d**, **f**) in different immune and culture conditions. 1st level compares hypoxic cells to normoxic cells in four different conditions: resting and single culture (comparison A_M_/A_F_ in pink), resting and coculture (B_M_/B_F_ in blue), LPS+IFNγ and single culture (C_M_/C_F_ in green), LPS+IFNγ and coculture (D_M_/D_F_ in yellow); 2nd level (double arrows) compares coculture to single culture conditions (AvsB and CvsD); 3rd level (triple arrows) compares resting to proinflammatory conditions [(A+B)vs(C+D)]. **b**, **d** Venn diagrams with DEGs distribution in each 1st level comparison in Mφ (**b**) and Fb (**d**). **e**, **f** Pathways enrichment analysis in Mφ (**e**) and Fb (**f**) comparisons. Columns represent 1st level analysis (A-B-C-D), rows report pathways significantly modulated (|z-score | ≥ 2) in at least one comparison. Color intensity bar indicates the level of positive (in red) or negative (in blue) enrichment; dots appear only when pathways are significantly enriched; gray indicates no modulation. See also Fig. S5. **g** Schematic representation of cellular changes in the key condition which feeds assumption B in the mathematical model. Source data of heatmaps **e**, **f** are provided as a Source Data file.

Reduction of oxygen tension induces a metabolic switch in cells^33–35^. In our experimental setting, hypoxia (1% O_2_) regulated a restricted set of DEGs in Mφ and Fb, including well-known hypoxia-responsive genes such as *GLUT1*, *VEGFA*, *CXCR4*, and *BNIP3* (Fig. S4a–c and S4d–f for Mφ and Fb, respectively).When we compared the global effect of hypoxia in single cell cultures (Fig. 4 and S5; comparisons A_M_ and A_F_ for Mφ and Fb, respectively) with its effects in coculture setting (Fig. 4 and S5; comparisons B_M_ and B_F_ for Mφ and Fb, respectively), we observed a dramatic increase in hypoxia-regulated DEGs when the two cell types were stimulated in tissue-like conditions (Fig. 4b, d, respectively). Conversely, the concomitant presence of proinflammatory conditions had no significant impact on the effects of hypoxia on neither Mφ nor Fb, neither in single cell nor in coculture conditions (comparisons in Fig. S5; C_M_ and D_M_ for Mφ and C_F_ and D_F_ for Fb, respectively). Similar findings were obtained when the effects of IL-4 were analyzed (Fig. S5; comparisons E_M_ and F_M_ for MF and E_F_ and F_F_ for FbF, respectively).

Functional analysis performed on macrophages hypoxia-responsive DEGs showed that hypoxia regulated pathways were related to actin remodeling, extracellular matrix deposition, and proliferation (Fig. 4e and Supplementary Data 3). Of note, these effects of hypoxia were evident only when Mφ were cocultivated with Fb, and were abolished when cells were exposed to proinflammatory or Th2 immune stimuli (Fig. 4 and S5). A similar regulation of hypoxia effects on the cell transcriptome was observed in Fb, where hypoxia regulated DEGs related to inflammation and leukocyte recruitment (Fig. 4f and Supplementary Data 3).

We conclude that hypoxia has a deep impact on both Mφ and Fb specifically when these cells can establish intercellular networks, with Mφ acquiring an active and proliferative phenotype and Fb assuming proinflammatory and angiogenic properties. These findings constitutes the basis for assumption B of our mathematical model (Fig. 4g). Our results also suggest that this effect is mostly relevant in tissues exposed to hypoxic conditions in the absence of significant immune reactions, while the specific contribution of hypoxia to the cells transcriptome is significantly reduced when inflammatory/immune triggers concomitantly act on Mφ and Fb (Fig. 4e, f).

### Functional interactions between Mφ and Fb are enhanced in hypoxic and inflammatory conditions

**Fig. 5:**
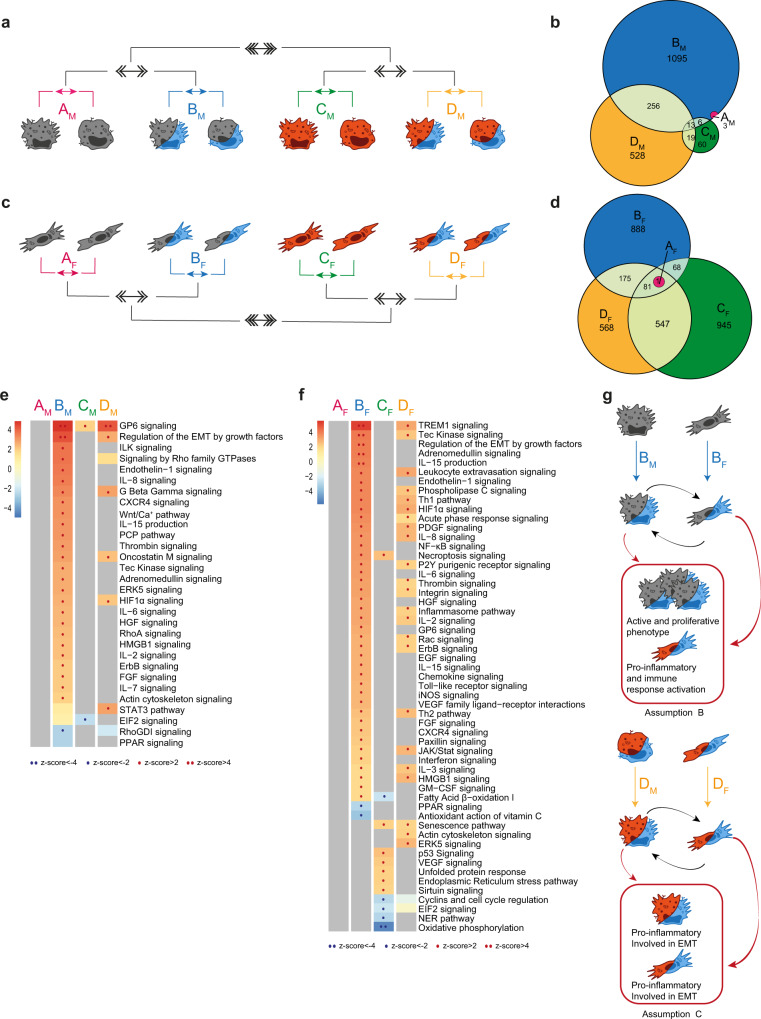
Role of cellular interplay on the global biological response. **a**, **c** Schematic representation of the multi-level approach to study the effect of Fb on Mφ (**a**, **b**, **e**) and of Mφ on Fb (**c**, **d**, **f**) in a tissue-like setting with different metabolic and immune conditions.1st level compares cocultivated cells to single cultivated cells in four different conditions: in normoxic and resting (comparison A_M_/A_F_ in pink), in hypoxic and resting (B_M_/B_F_ in blue), in normoxic and inflamed (C_M_/C_F_ in green), in hypoxic and inflamed (D_M_/D_F_ in yellow). 2nd level (double arrows) compares normoxic to hypoxic environments (AvsB and CvsD) and 3rd level (triple arrows) compares resting to proinflammatory conditions [(A+B)vs(C+D)]. **b**, **d** Venn diagrams show the distribution of DEGs in each 1st level comparison in Mφ (**b**) and Fb (**d**). **e**, **f** Pathways enrichment analysis in Mφ (**e**) and Fb (**f**) comparisons. Columns represent 1st level of comparisons (A-B-C-D), rows report pathways significantly modulated (|z-score | ≥ 2) in at least one comparison. Color intensity bar indicates the level of positive (in red) or negative (in blue) enrichment; dots appear only when pathways are significantly enriched; gray indicates no modulation. See also Fig. S6. **g** Schematic representation of cellular changes in the key condition which feeds assumptions B and C in the mathematical model. Source data of heatmaps **e**, **f** are provided as a Source Data file.

Cellular communication is a key element to adopt appropriate complex responses to stimuli of various origin (mechanic, chemical, immune, metabolic)^36^. We took advantage of our in vitro setting to specifically investigate how an immune cell, such as the Mφ, interacts with a stromal cell, represented by the Fb, in different immune and metabolic contexts and how this interaction affects the response to these cues. The comparison of cocultivated Mφ and Fb to their single cultivated counterparts revealed that in resting conditions (i.e., normoxia, absence of inflammation and immune challenging) cocultivated cells did not differ from single cultivated counterparts (1st level analysis; comparisons A_M_ and A_F_ in Fig. 5 and Fig. S6). Coculture also did not affect Mφ response to inflammatory triggers, while the presence of Mφ has significant impact on Fb response to inflammation (comparisons C_M_ and C_F_ in Fig. 5, respectively, and Fig. S6). At variance, coculture significantly affected the activation of both cell types in response to hypoxia, both in the absence (comparisons B_M_ and B_F_ in Fig. 5 and S6) and in the concomitant presence of hypoxia and inflammation (comparisons D_M_ and D_F_ in Fig. 5 and S6). Of note, coculture in Th2-conditioned environment induced no changes nor in Mφ neither in Fb; however, IL-4-conditioned Mφ in hypoxic and tissue-like environment are able to block the switch of Fb into proinflammatory phenotype (Fig. 3g). We conclude that Fb have an impact on Mφ activation only if this interplay takes place in hypoxic conditions, while Mφ influence Fb response to hypoxia and to inflammation independently from oxygen tension levels.

Functional analysis showed that, when cells were challenged with hypoxia, coculture significantly enriched the expression of genes related to intercellular communication in both Mφ and Fb (Fig. 5e, f and Supplementary Data 4). These functional networks related to cell-cell interactions are likely responsible for the enhanced expression of genes related to cell proliferation observed in Mφ exposed to hypoxia in the presence of Fb and genes related to inflammation, particularly evident in Fb exposed to hypoxia in the presence of Mφ. When hypoxia operates in an inflammatory setting, a reduction in the number of enriched pathways was observed both in Mφ and Fb. In functional terms, whereas in these experimental conditions cocultivated Fb maintained an enriched expression of genes related to inflammation, cocultivated Mφ showed a significant enrichment only in pathways related to cell-cell interaction and hypoxia, while a gene signature related to cell proliferation was not evident anymore. Finally, Fb were susceptible to signals derived from the presence of Mφ also in inflammatory settings, irrespective of the presence of hypoxia. In this setting, their response showed an upregulation of genes mainly related to cell cycle control, cell response to stress, and senescence. We conclude that in normoxic conditions the establishment of an inflammatory milieu in the tissue supports the ability of Mφ to induce cellular stress and activate senescence programs in Fb. On the opposite, in absence of an immune challenge, the interplay between Fb and Mφ occurring in tissues in response to hypoxia modifies the response of both cell types, promoting the acquisition of proliferative properties in Mφ and inducing a proinflammatory phenotype in Fb. Of note, these effects of hypoxia are significantly attenuated when the Mφ/Fb interplay occurs in an inflammatory setting. These changes in cell response will be applied as assumptions B and C in our mathematical model (Fig. 5g).

### Different tissue districts in fibrotic kidneys are characterized by distinct patterns of immune infiltrate and transcriptional profiles

These in vitro results indicate the interaction between Fb and Mφ profoundly influence the functional effects of immunological and metabolic cues operating in tissues, likely affecting their role in fibrosis development. Therefore, translation of these in vitro findings to specific human diseases requires considering the fine anatomy of the involved organ, which influences the likelihood of direct Mφ/Fb interactions and the degree of hypoxia.

The kidney is composed of different tissue compartments, which in the cortical area include the glomeruli and the tubulointerstitial compartments. It also presents a unique type of vascularization, with each individual glomerulus connected to the circulation by an exclusive afferent and efferent arteriolar vessels, the latter continuing into longitudinally arranged capillaries along the tubules. The distinct anatomy of the interstitial compartment and the presence of kidney-specific epithelial boundary structures (e.g., the Bowman capsule confining glomeruli) were our rationale to investigate the potential relevance of tissue organization for fibrosis development. We therefore investigated the cellular composition of different compartments of the cortical tissue by multiplexed immunohistochemistry (mIHC) and used laser capture microdissection (LCM) to enrich transcriptional programs in distinct anatomic districts defined as reported in the Methods section: the glomerulus; the 200–250 µm surrounding area (from now referred to as “surrounding”), which represent the range where cytokine/chemokine gradients control motility of immune cells and their mutual communication^37,38^; the tubulointerstitial area (from now referred to as “interstitium”). Comprehensive immune cell phenotyping and transcriptional analysis was performed in these anatomical areas on transplanted kidneys at different stages of fibrosis and in control samples, represented by non-fibrotic tissue specimen obtained from tumor-distant areas of kidneys removed for renal cell carcinomas.

**Fig. 6:**
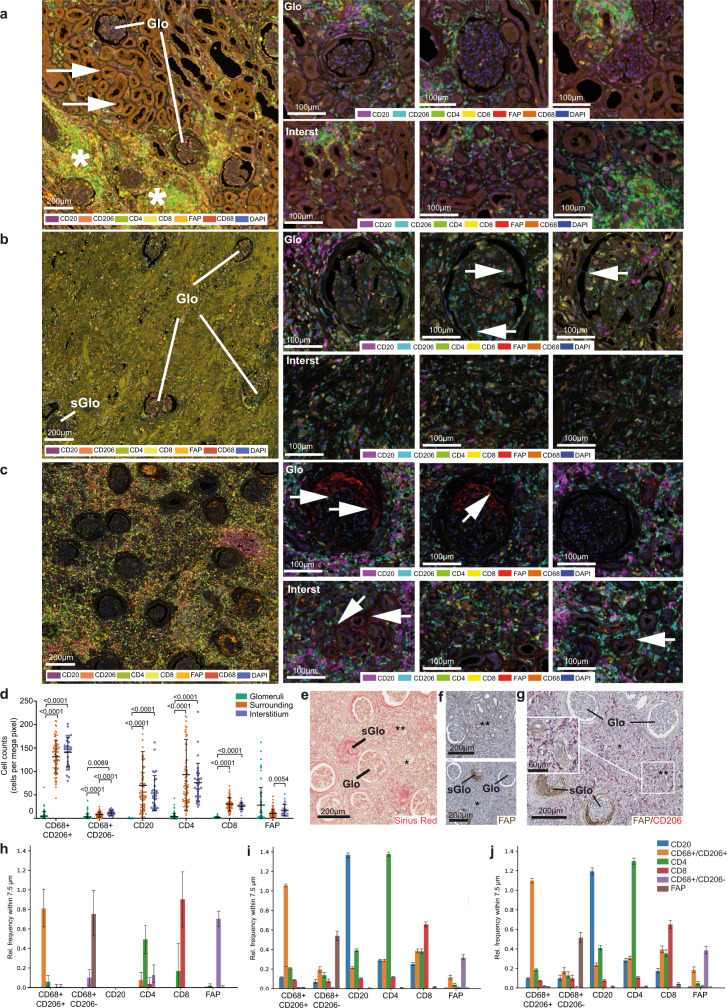
Different stages of fibrosis and heterogeneity of immune cell infiltrates characterizing distinct anatomical kidney regions. **a**–**c** Multiplexed immunohistochemistry of transplant nephrectomy samples in **a** acute cell-mediated rejection (TCMR; BANFF type IIa), **b** chronic antibody-mediated rejection (ABMR), and **c** active stage of fibrosis (ca. 90% IF/TA; BANFF category 5, grade III) with signs of chronic rejection consistent with combined TCMR and ABMR. The left panel of **a**–**c** shows fluorescent whole slide scans for overview in tissue context. The right three panels show region-specific immune cell phenotyping in multiple fields of view after multispectral unmixing (see color code in right panel of **a**–**c**). While multiplexed immunohistochemistry was performed in only a single final experiment, all immunohistochemical staining results for individual markers (FAP, CD206, CD68, CD4, CD8, CD20) have been systematically controlled by chromogenic single- and duplex immunohistochemistry on consecutive sequential sectioning levels (Fig. 6f, g, and ref. 27). Representative examples of the glomerular compartment (Glo) and the tubulointerstitial areas (Interst) are displayed. Co-existence of almost normal Glo and tubulointerstitial areas (**a**, left panel, arrows) and severely inflamed regions (**a**, left panel, asterisks) can be observed. Glomeruli were almost devoid of Mφ with few exceptions (**b**, right panel, arrows). If present in glomeruli, Mφ were rarely in direct neighborhood to FAP^+^ myofibroblasts (**c**, right panel, arrows). **d** Scatter plot representing cell density of different cell types: macrophages (CD68^+^/CD206^+^, CD68^+^/CD206^−^), T lymphocytes (CD8^+^, CD4^+^), B lymphocytes (CD20^+^) and activated myofibroblasts (FAP^+^), in the same case as presented in (c) in each anatomical compartment (glomeruli (G), Points indicate results for each ROI, *N* = 79; surrounding (S), *N* = 79; interstitium (I), *N* = 31; center line represents mean values, whiskers depict SD. Two-way ANOVA with Tukey’s multiple comparisons test were used to evaluate the differences (*p*-values are reported on the graph). **e** Sirius red staining (corresponding to **c**) showing co-existence of morphologically almost intact Glo and sclerotic glomeruli (sGlo), and tubulointerstitial areas ranging from severe (**) to moderate/low fibrosis (*). **f** IHC for FAP (corresponding to **c**), showing co-existence of almost normal Glo and areas of massive myofibroblast activation (sGlo), and tubulointerstitial areas ranging from severe (**) to moderate/low myofibroblast activation (*). **g** Duplex immunohistochemistry (corresponding to **c**) confirmed the heterogeneous distribution of activated myofibroblasts (FAP^+^), and alternatively activated CD206^+^Mφ in different anatomical regions. **h**–**j** Neighborhood analysis on multiplexed IHC. For each region, glomeruli (**h**), interstitium (**i**) and surrounding (**j**), each cell of interest indicated on the horizontal axis (CD68^+^/CD206^+^,CD68^+^/CD206^−^, CD8^+^,CD4^+^,CD20^+^, and FAP^+^) was evaluated for the relative frequency of the corresponding cell types (vertical axis) within a radius of 7.5 μm. Data are presented as mean values (top of bar graph) with SD (whiskers). The number of analyzed cells was (**h**) CD68^+^/CD206^+^ *n* = 99, CD68^+^/CD206^−^ *n* = 50, CD8^+^ *n* = 18, CD4^+^ *n* = 78, CD20^+^ *n* = 0, and FAP^+^ *n* = 462; **i**CD68^+^/CD206^+^ *n* = 15179, CD68^+^/CD206^−^ *n* = 1356, CD8^+^ *n* = 3659, CD4^+^*n* = 9286, CD20^+^ *n* = 4756, and FAP^+^ *n* = 1443; **j** CD68^+^/CD206^+^ *n* = 1072, CD68^+^/CD206^−^ *n* = 37, CD8^+^ *n* = 265, CD4^+^ *n* = 829, CD20^+^ *n* = 810, and FAP^+^ *n* = 69. Source data of the multiplex immunohistochemistry experiment depicted in Fig. 6d, h–j are provided as Source data files and in Zenodo repository.

Conventional microscopy and mIHC showed that different stages and degrees of fibrosis coexist within a specimen. Some morphologically almost intact glomeruli and normal-appearing tubular structures were still present even in kidneys with terminal graft failure after different types of chronic rejection (Fig. 6a, b). In an earlier stage of fibrosis, a broad range from severely affected to almost intact glomeruli (Fig. 6c, right upper panels) and locally different degrees of inflammation in interstitial areas (Fig. 6c, right lower panels) were present (Fig. 6e, f). Spatial heterogeneity between tissue compartments was also observed for the composition of the immune cell infiltrate (Fig. 6d). CD4^+^ and CD8^+^ T cells showed increased density in the interstitium compared to the glomerular compartment, and CD20^+^ B cells were almost exclusively present outside the glomeruli. Mφ subsets also showed tissue compartment-specific distribution, with abundant CD206^+^/CD68^+^ alternatively activated Mφ highly represented in the interstitium and almost absent in the glomeruli (Fig. 6d). In contrast, activated (FAP^+^) Fb were present in all compartments, but significantly enriched in the glomeruli (Fig. 6g). Large-scale neighborhood analysis revealed that: (i) CD20^+^ B cells were largely clustered with themselves, with few Mφ in the direct neighborhood; (ii) mixed infiltrates composed of CD8^+^ effector and CD4^+^helper T cells tended to cluster together with dispersed Mφ of different types; (iii) FAP^+^ Fb were in relatively close contact to other immune cells in the interstitial area, while being predominantly located in neighborhood with each other in glomeruli, suggesting that their activation mechanisms were different in distinct tissue compartments (Fig. 6h–j).

As different tissue compartments showed different patterns of immune infiltrate, we compared the LCM-enriched sub-compartment-specific transcriptome profiles of the three distinct anatomical regions of interest (Fig. S7a). PCA showed that the anatomical area was the first source of variation among all samples, and that for each anatomical district investigated samples from control and fibrotic kidneys were clearly distinct (Fig. S7b). Gene ontology (GO) and pathways enrichment analysis revealed a number of functional categories significantly enriched in fibrotic samples, most of which related to immune response and ECM organization (Fig. S7c). We then investigated the potential relevance in vivo of the different cellular networks identified in vitro by defining their relative contribution to the transcriptional profiles detected in fibrotic reactions occurring in these different anatomical districts. To this purpose, GSEA and overlap analysis were applied to define the enrichment of specific signatures identified by in vitro comparisons in the ex vivo signature defined by the comparison between fibrotic kidneys and controls. Overall, our in vitro signatures were able to explain 45.3% of total DEGs that discriminated fibrotic kidneys from controls. This percentage included genes shared by more than one signature (35.6%) as well as genes only present in a single signature (9.7% in total). A limited number of experimental in vitro conditions could explain each a significant fraction (up to 20%) of the ex vivo gene signature (Fig. S7d, middle panel). Most of these gene sets were shared among two or more settings, but some provided a univocal contribution (i.e., DEGs detected in the ex vivo signature only present in one specific in vitro experimental comparison) shading light on the biological processes occurred in the tissue. Gene sets with a univocal signature were grouped depending on the discriminating variable that defines in vitro comparison. We observed that univocal signatures that better describe fibrotic kidneys presented inflammation as a major trigger, being other variables investigated in the in vitro system (IL-4, cell-cell interaction, hypoxia) able to explain only a minor number of genes characterizing fibrotic kidneys (Fig. S7d, left panel). Considering both univocal and general contribution of in vitro signatures, we observed that genes associated with leukocyte activation and inflammatory events occurring in fibrotic kidneys were mostly overlapping with our in vitro data related to Mφ subjected to inflammatory stimuli mainly in coculture setting, independently of oxygen level. On the other side, in vitro signatures describing Fb subjected to Mφ influence in hypoxic environment provided a description of fibrotic and angiogenic properties characterizing fibrotic kidneys compared to control samples (Fig. S7d, right and middle panels).

### Enrichment of proinflammatory signatures outside glomeruli during renal allograft rejection

**Fig. 7:**
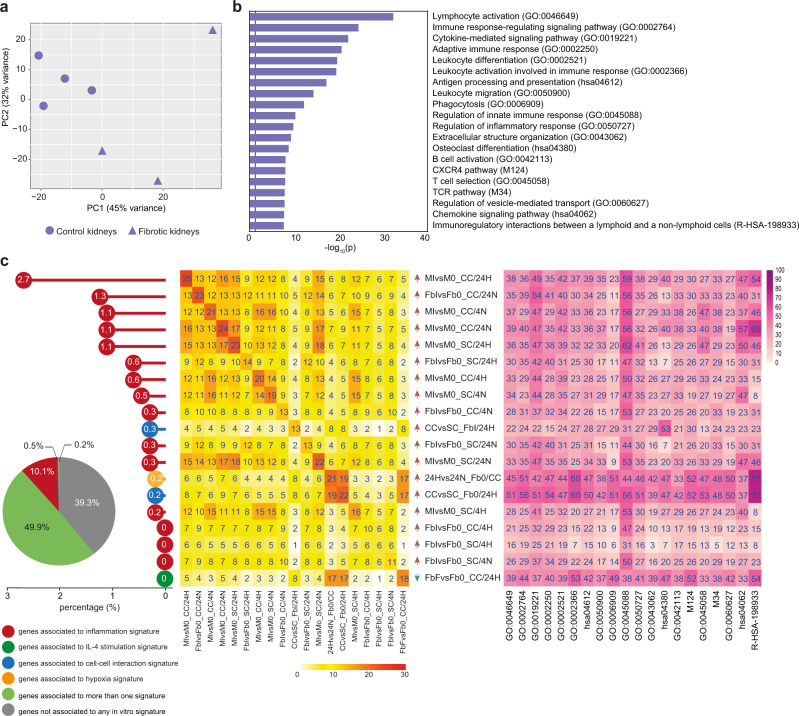
Gene overlap analysis of ex vivo tubulointerstitial area from fibrotic kidneys with in vitro signatures. **a** PCA of ex vivo interstitium samples. Triangles indicate fibrotic kidneys, dots control kidneys. **b** Bar graph reports pathways significantly enriched (log_10_ (*p*-value) ≥1.3) in fibrotic vs control interstitium samples as calculated by Metascape software (for statistical type of analysis see ref. 45). **c** Overlap analysis was performed comparing DEGs of ex vivo fibrotic vs control interstitium with DEGs of each in vitro comparison (which defined 78 corresponding signatures). Pie chart reveals that 49.9% of ex vivo signature is explained by at least one in vitro signature (in light green); 10.8% is specifically explained by only one in vitro signature and different colors are referred to the discriminating variable: inflammation in red, IL-4 stimulation in green, cell-cell interaction in blue, hypoxia in yellow. The remaining 39.3% of the ex vivo interstitium signature (633 genes) is not explained by in vitro model (in gray). Dot chart reports on the vertical axis 19 in vitro signatures enriched in ex vivo model and their relative contribution with unique expressed genes: white numbers in each dot represent relative percentages on the totality of explained and not explained genes. The central heatmap highlights the overlapping percentage of the same 19 in vitro signatures on the ex vivo signature. On the diagonal, numbers indicate the overlapping percentage of each signature; the other numbers explain the overlapping percentage shared between two in vitro and the ex vivo signatures. Color intensity indicates the overlapping percentage level referred to ex vivo signature. Arrows near each in vitro signature indicate the positive (red) or negative (blue) enrichment of the first term of in vitro comparison (see also Fig. S8). The heatmap on the right reports Metascape pathways in ex vivo comparison (**b**) with the percentage of enrichment given by each in vitro signature (on the left).

**Fig. 8:**
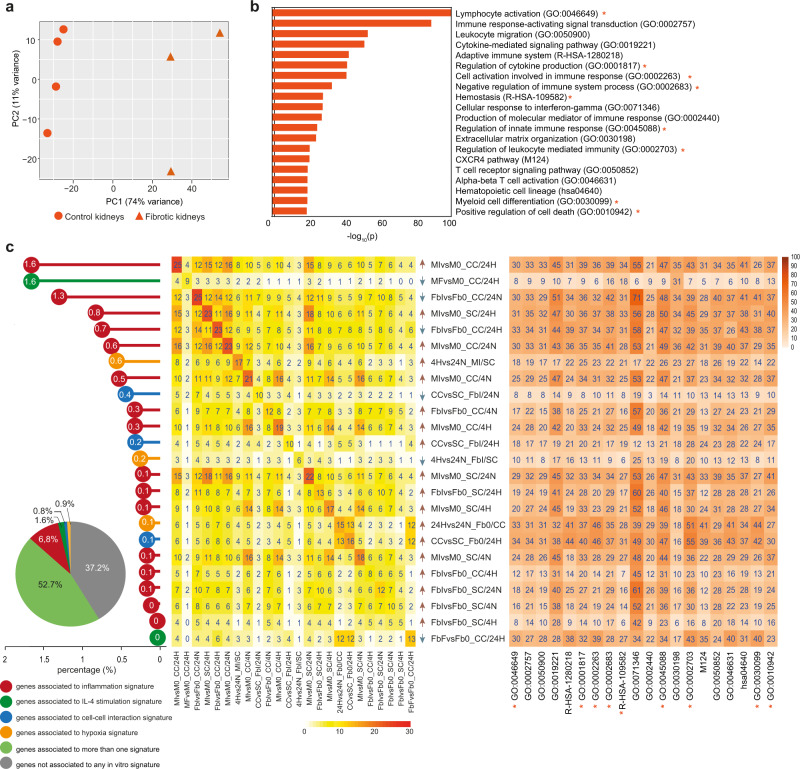
Gene overlap analysis of ex vivo glomeruli surrounding area from fibrotic kidneys with in vitro signatures. **a** PCA of ex vivo glomeruli surrounding area samples. Triangles indicate fibrotic kidneys, dots control samples. **b** Bar graph reports pathways significantly enriched (log_10_ (*p*-value) ≥1.3) in pathological vs control surrounding samples as calculated by Metascape software (for statistical type of analysis see ref. 45). **c** Overlap analysis was performed comparing DEGs of ex vivo fibrotic vs control surrounding areas with DEGs of each in vitro comparison (which defined 78 corresponding signatures). Pie chart reveals that 52.7% of ex vivo signature is explained by at least one in vitro signature (in light green); 10.1% is specifically explained by only one in vitro signature and different colors are referred to the discriminating variable of in vitro signatures: inflammation in red, IL-4 stimulation in green, cell-cell interaction in blue, hypoxia in yellow. The remaining 37.2% of the surrounding ex vivo signature (2685 genes) is not explained by in vitro model (in gray). Dot chart reports on the vertical axis 24 in vitro signatures enriched in ex vivo model and their relative contribution with unique expressed genes: white numbers in each dot represent relative percentages on the totality of explained and not explained genes. The central heatmap highlights the overlapping percentage of the same 24 in vitro signatures. On the diagonal, numbers indicate the overlapping percentage of each signature; the other numbers explain the overlapping percentage shared between two in vitro and the ex vivo signatures. Color intensity indicates the overlapping percentage level referred to ex vivo signature. Arrows near each in vitro signature indicate the positive (red) or negative (blue) enrichment of the first term of in vitro comparison (see also Fig. S8). The heatmap on the right reports Metascape pathways in ex vivo comparison (**b**) with the percentage of enrichment given by each in vitro signature (on the left). Orange stars highlight pathways that are specifically enriched in surrounding nephrectomies and are not shared with other renal districts (interstitium, glomeruli).

**Fig. 9:**
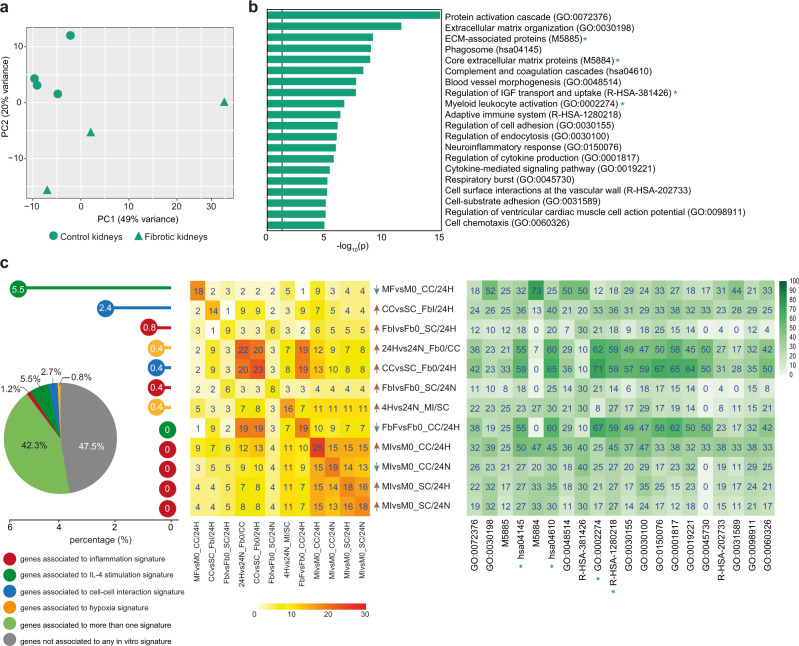
Gene overlap analysis of ex vivo glomeruli from fibrotic kidneys with in vitro signatures. **a** PCA of ex vivo glomeruli samples. Triangles indicate fibrotic kidneys, dots control samples. **b** Bar graph reports pathways significantly enriched (log_10_ (*p*-value) ≥1.3) in fibrotic vs control glomeruli samples as calculated by Metascape software (for statistical type of analysis see ref. 45). **c** Overlap analysis was performed comparing DEGs of ex vivo fibrotic vs control glomeruli with DEGs of each in vitro comparison (which defined 78 corresponding signatures). Pie chart reveals that 42.3% of ex vivo signature is explained by at least one in vitro signature (in light green); 10.2% is specifically explained by only one in vitro signature and different colors are referred to the discriminating variable of in vitro signatures: inflammation in red, IL-4 stimulation in green, cell-cell interaction in blue, hypoxia in yellow. The remaining 47.5% of the ex vivo glomeruli signature (255 genes) is not explained by in vitro model (in gray). Dot chart reports on the vertical axis 12 in vitro signatures enriched in ex vivo model and their relative contribution with unique expressed genes: white numbers in each dot represent relative percentages on the totality of explained and not explained genes. The central heatmap highlights the overlapping percentage of the same 12 in vitro signatures on the ex vivo signature. On the diagonal, numbers indicate the overlapping percentage of each signature; the other numbers explain the overlapping percentage shared between two in vitro and the ex vivo signatures. Color intensity indicates the overlapping percentage level referred to ex vivo signature. Arrows near each in vitro signature indicate the positive (red) or negative (blue) enrichment of the first term of in vitro comparison. (See also Fig. S8). The heatmap on the right reports Metascape pathways in ex vivo comparison (**b**) with the percentage of enrichment given by each in vitro signature (on the left). Green stars highlight pathways that are specifically enriched in glomeruli nephrectomies and are not shared with other renal districts (interstitium, surrounding area).

When distinct anatomical regions were investigated, PCA confirmed the clustering of fibrotic and control samples in each district (Figs. 7a, 8a, and 9a for the interstitium, the surrounding area, and glomeruli, respectively). Both in the interstitium and in surrounding regions we confirmed the enrichment of inflammatory pathways in pathological samples, with GO terms related to leukocyte activation and migration, adaptive immune response, cellular response to IFNγ, regulation of cytokine production being clearly enriched in fibrotic samples (Figs. 7b and 8b). Our in vitro signatures explained about 60% of the fibrosis-related signatures emerging in these two anatomical districts, with an evident predominance of signatures related to inflammation testified by 10% of DEGs included in functional categories emerging exclusively in response to inflammatory triggers used in vitro. In both anatomical districts, the in vitro generated signature contributing the most to DEGs observed in vivo was related to Mφ response to inflammatory stimuli, requiring however the concomitant presence of Fb and hypoxia (MIvsM0_CC/24H signature; Figs. 7c and 8c). Of note, in the interstitium all signatures providing significant contribution to explain DEGs observed ex vivo were related to response to inflammation, either by Mφ or Fb. At variance, a distinct signature associated with Mφ response to IL-4, again requiring the concomitant presence of Fb and hypoxia, emerged only in the surrounding tissue (MFvsM0_CC/24H signature; Fig. 8c, left and middle panels), and appeared to be specifically related to the GO category ECM organization (Fig. 8c, right panel). In vitro signature enrichment was validated and confirmed also by GSEA analysis (Fig. S8, violet and orange boxes).We conclude that, both in the interstitium and in the surrounding area, proinflammatory signatures explain most of DEGs characterizing fibrotic samples, with many DEGs provided by inflammatory Mφ activated in the presence of hypoxia (Figs. 7c and 8c, right panels). Interestingly, the presence only in the surrounding area of a signature associated with Mφ response to IL-4 suggests that different biological networks support fibrosis development in these two anatomical districts.

### Enrichment of IL-4 conditioned signature in fibrotic glomeruli during renal allograft rejection

Pathological glomeruli from fibrotic kidneys were clearly distinct from the corresponding control samples (Fig. 9a) and showed enrichment in pathways related to ECM organization, blood vessel morphogenesis, and cellular adhesion (Fig. 9b). In vitro signatures were clearly different from signatures that enriched the other renal districts previously described. Indeed, the contribution of inflammation-related pathways was reduced (1.2%; Fig. 9c, left panel), and a unique signature corresponding to IL-4-activated Mφ in the concomitant presence of Fb and hypoxia emerged, which contributed the most to describe DEGs enriched in the glomeruli area from fibrotic kidneys (MFvsM0_CC/24H; Fig. 9c, left and central panels). Of note, this signature explained more than 50% of DEGs that contributed to GO terms related to ECM deposition and blood vessels in these pathological samples (Fig. 9c, right panel). Signatures related to Fb interacting with Mφ, in hypoxic conditions but independently from inflammation, also significantly contributed to the description of glomeruli from fibrotic kidneys (CCvsSC_FbI/24H, 24Hvs24N_Fb0/CC, CCvsSC_Fb0/24H; left and central panels in Fig. 9c, green box in Fig. S8). Interestingly, these Fb-related in vitro signatures explained a large fraction (from 30% to 70%) of DEGs associated with different GO categories related to cytokine signaling, immune response, cell adhesion, and chemotaxis (Fig. 9c, right panel). However, while all Fb-related GO categories were not specific for pathological glomeruli, as genes that enriched those pathways were also contributing to the interstitium and surrounding areas, most Mφ-related signatures were only evident in this anatomical compartment, suggesting that IL-4 conditioned Mφ interacting with Fb in hypoxic areas provide a specific spatial-restricted driver in the glomeruli area in fibrotic kidneys.

### A mathematical model based on in vitro signatures identifies three dynamic states in fibrosis development

**Fig. 10:**
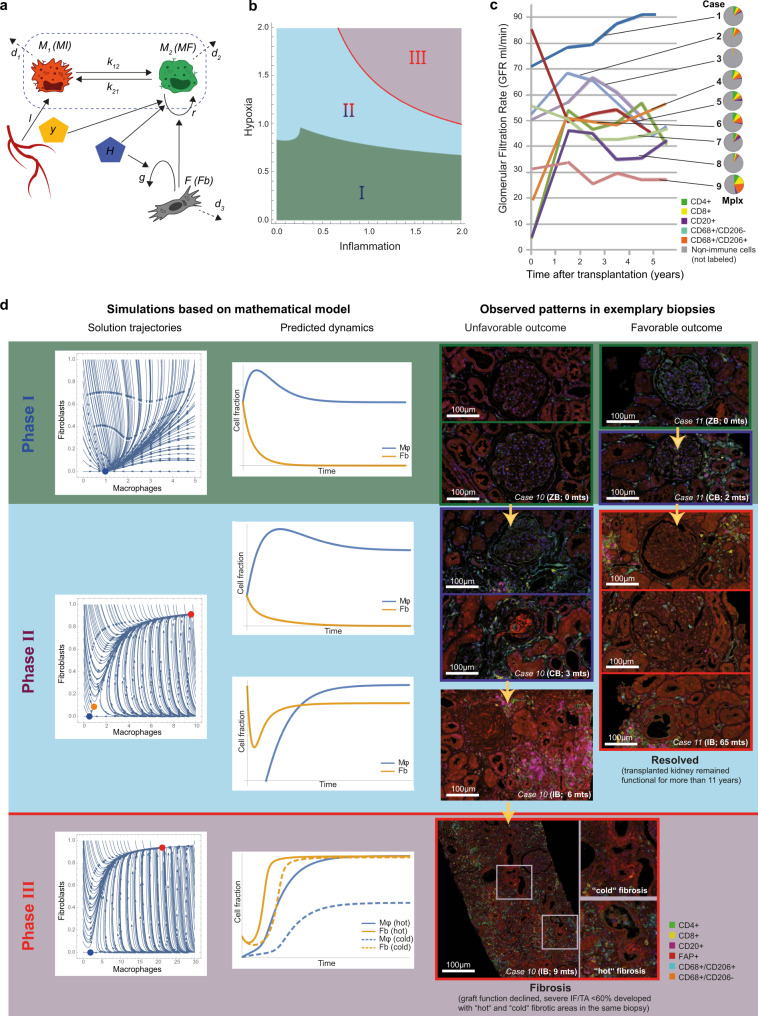
Mathematical model based on in vitro data and exemplary clinical observations reflecting the predicted outcomes. **a** Schematic representation of Mφ (MI in red and MF in green) and Fb interactions under hypoxia (H) and inflammation (Y). Details are provided in Methods section. **b** Phase diagram of mathematical prediction. Three phases (I-II-II) were identified on different levels of inflammation (horizontal axis) and hypoxia (vertical axis). The red line between phases II and III is the separatrix. **c** Nine exemplary cases (cases 1–9, for clinical details see Supplementary Table 1) reflecting the stable “Phase I”: The curves depict the approximate glomerular filtration rate (GFR) over time (for exact GFR measurements and time points of biopsies, see Fig. S11). Pie charts describe the individual immune cell infiltrate (lymphocytes and Mφ) evaluated by quantitative digital pathology after multiplex immunohistochemistry (Mplx) (source data are provided as Source data file). The percentage of immune cells (color code in the figure legend indicates the immune phenotype) in comparison to all nucleated cells in the biopsy as defined by DAPI nuclear staining (gray area in the pie charts) can massively vary between almost no inflammation (e.g., case 3, 8) and abundant infiltrates (e.g., cases 5, 6, 9). **d** Graphical depiction of in silico simulations (left panels) show the solution trajectory and the predicted dynamics of Mφ and Fb depending on time (horizontal axis) and cell fraction (vertical axis) for each of the three phases as predicted by the mathematical model in more detail. The right panel shows corresponding representative clinical constellations that confirmed the hypotheses generated by computer simulations, based on the exemplary cases 10 (unfavorable outcome with irreversible “hot” and “cold” fibrosis) and case 11 (favorable outcome despite “borderline” immune infiltration). The color legend indicates the different cell types as detected my multiplexed immunohistochemistry. Note the abundant CD206^+^ macrophages in the indicated biopsy of case 11 in “Phase II” in the surrounding area (upper image), inside the glomerulus (middle image) and in the interstitial compartment (bottom image), and in case 10 in “Phase III” the co-existence of areas with many CD206^+^ macrophages (“hot” fibrosis) and low density of this phenotype (“cold” fibrosis) co-localized with myofibroblast activation (FAP^+^ cells) in the same biopsy as predicted by in silico modeling.

The combination of our in vitro and ex vivo transcriptomic approaches identified the key parameters driving the interplay between Mφ and Fb in different biological settings, their specific functional implications, and their correspondence with fibrotic events occurring in vivo in different anatomical regions (Fig. S1a). Here, we exploited this information to develop a mathematical model able to translate the Mφ/Fb interactions in vitro identified into their corresponding in vivo dynamics (Fig. 10a). Specifically, the model was based on literature information showing that inflammatory Mφ are mainly sustained by local recruitment in inflamed tissues while alternative Mφ accumulation can be sustained by their proliferative abilities^39^, and that Mφ phenotypic switching can occur relatively fast and at shorter time point compared to their proliferation/death rate^40^, integrated by the three key observations emerged in our in vitro analysis related to inflammation and hypoxia:

A. when inflammatory conditions develop in the absence of significant hypoxia, both Mφ and Fb develop a proinflammatory phenotype but also activate a senescence program (comparisons C_M_ and C_F_ in Fig. 2e, f);
B. in hypoxic conditions, Mφ acquire proliferative properties while Fb assume a proinflammatory phenotype (comparisons B_M_ and B_F_ in Fig. 4e, f, comparisons B_M_ and B_F_ in Fig. 5e, f);
C. when inflammatory conditions and hypoxia are combined, Fb remain proinflammatory while Mφ become involved in regulation of epithelial-mesenchymal transition and angiogenesis (comparisons D_M_ and D_F_ in Fig. 5e, f).

The above has been translated into the assumptions A1–A5 (see Methods section), which allowed us to build our mathematical model. It is worth mentioning that as in vitro parameters can largely deviate from the in vivo (e.g., human macrophages do not proliferate in vitro but in vivo they do so), the model parameters have not been calibrated to the in vitro kinetics. Instead, we have non-dimensionalised the model and exhaustively explored the corresponding parametric space. After model analysis, we identified three dynamic phases/regimes resulting from the inflammation/hypoxia interplay (Fig. 10b), which were reflected in renal biopsies representative of cases with different clinical courses (Fig. 10c) and in cases experiencing clinically manifest rejection (Fig. 10d). Interestingly, qualitatively these dynamics regimes were robust under large variations of the model parameters (for details see Figs. S9 and S10).

Phase I is characterized by a low degree of hypoxia, independently of inflammation that could range from low to high levels (green area/panel in Fig. 10b, d, respectively). This regime is characterized by model conditions where the predictive cell trajectory starts with an arbitrary number of cells and ends with a particular amount of Mφ dependent on the local inflammatory signals, while all conditions are characterized by a very low number of Fb. A specific example of a clinical situation reflecting this stable *quasi*-healthy state can be found in “protocol” biopsies, which are obtained from patients at a defined time point after uncomplicated renal transplantation, as exemplified by cases of variable inflammation in the protocol biopsy 6 months after renal transplantation and favorable long-term clinical course (stable renal function over 2–5 years as shown in nine exemplary cases (cases 1–9, Fig. 10c)): various levels of inflammation can be present, but since there is no relevant hypoxia the system can always return to normal. Another example consistent with phase I could be considered “time-point zero” biopsies taken at very early time points during or shortly after the uncomplicated surgical procedure as shown in two exemplary cases (case 10 and 11, Fig. 10d, green upper panel). In this setting, very close to the physiological situation of the kidney in the donor, the recipient’s immune system did not yet have time to respond to the allograft, and consequently we do not yet observe severe inflammation.

Phase II, a bistable dynamic regime that can lead either to healthy or fibrotic states, is characterized by wide range of tolerable inflammation and hypoxia levels (blue area/panel in Fig. 10b, d, respectively). The critical quantity for this regime is the initial condition of Mφ and Fb. In particular, there is a separatrix indicating that only high Mφ/low Fb or vice versa may lead to a healthy renal state, with all the other initial conditions with moderate or high Mφ and Fb levels are predicted to evolve into fibrosis development. Clinical examples reflecting such bistable condition include so-called “borderline” states, mostly observed in “indicated” biopsies that are obtained for diagnostic purposes in clinical situations requiring a decision on potential adaptation of the current immunosuppressive therapy. “Borderline” is a microscopic pattern defined by the Banff classification as evident inflammation that is suspicious, but not sufficient to justify the diagnosis of rejection. Borderline states, but also some cases of manifest rejection can still resolve, e.g., case 11 that resolved into long-term sufficient renal function after “borderline” diagnosis in an indicated biopsy after 65 months in (Fig. 10d). Both clinical conditions, borderline and some cases of manifest rejection can be successfully treated and may return to a healthy state, but a chance of turning into a profibrotic state remains.

Phase III (red area/panel in Fig. 10b, d, respectively) is the fully pathological, compromised state with an unfavorable combination of hypoxia and inflammation. It can be associated with “hot” or “cold” fibrosis (with or without persisting presence of immune cells, respectively). In case of “hot” fibrosis, both Mφ and Fb are highly concentrated, whereas in case of “cold” fibrosis only the number of Fb remains high. Exemplary cases that clinically represent such states include persistent viral infection with BK virus (Fig. 10d, case 10, left panel of the clinical examples) and irreversible end-stages after chronic rejection (Fig. 6a, b).

The mathematical model predicts a potential favorable outcome for phases I and II, which is consistent with the clinical observation of successful transplants in case of high Mφ infiltration but low Fb number, even in the presence of dense immune cell infiltrates (Fig. 10c). Of note, we found an explicit equation separating stable (phases I and II) from clinically relevant conditions (phase III) which dictates:

$${{{{{\rm{Hypoxia}}}}}} \ < \ \frac{{{{{{\rm{Pro}}}}}}-{{{{{\rm{inflammatory\; activity}}}}}}}{{{{{{\rm{Fibroblast\; viability}}}}}}}$$

where proinflammatory activity corresponds to the combined effect of proinflammatory Mφ polarization (λ), proinflammatory T- and B-cell presence (y) and anti-inflammatory decay rate and Fb viability is the ratio of Fb proliferation/apoptosis (mathematical details are in Methods section).

## Discussion

Although current treatments for fibrotic diseases typically target the inflammatory response, there is accumulating evidence that the mechanisms driving fibrogenesis are distinct from those regulating inflammation^1^. The key cellular mediator of fibrosis is the myofibroblast, which is activated by a variety of mechanisms including autocrine factors and paracrine signals derived from immune cells. In particular, in tissue repair and fibrosis Mφ exert relevant effects, which go beyond their proinflammatory activities and include Fb activation^41^. As highlighted by ref. 25, the reciprocal Mφ-Fb interaction is a key component for understanding observations such as the fibrotic time-window, the long timescale of scar maturation, and the paradoxical effect of Mφ depletion. Equally important are the environmental metabolic status and the cytokines/growth factors networks, being these parameters capable to influence Mφ functions and their ability to interact with neighboring cells, including Fb, as well as other immune cells such as lymphocytes. In our study, we implemented these immune and metabolic stimuli in a simplified in vitro system where cell circuits recreated a tissue-like scenario, giving at the same time the possibility to discriminate the weight of single variables and their combinatorial effects in the progression of the fibrotic process.

Mφ and Fb were not influencing each other when interacting in resting conditions, suggesting that cellular contacts without external stimuli are insufficient to induce major phenotype/functional changes. As reported, hypoxic conditions per se were able to activate a specific signature in both cell types, but surprisingly when the hypoxic environment acted on the two cell types directly interacting with each other, both Mφ and Fb regulated a significantly larger set of DEGs. This effect constitutes the first observation used as basis of our mathematical model. Similarly, the well-known strong response of Mφ to inflammatory mediators was highly influenced by the presence and absence of hypoxia and Fb. The concomitant exposure to inflammatory stimuli and hypoxic coculture conditions also influenced Fb, which acquired a clear proinflammatory phenotype, while Mφ remained involved in ECM remodeling and angiogenesis. This effect constitutes the second observation at the basis of our mathematical model. Instead, when Mφ and Fb were cocultivated in normoxic but proinflammatory environment, they both acquired proinflammatory properties, suggesting that in this context proinflammatory factors were sufficient to promote phenotypic changes without the contribution of metabolic switch induced by hypoxia. Moreover, both cell types downregulated oxidative phosphorylation and the EIF2 signaling pathways, indicating the activation of senescence processes that we suppose necessary to the exhaustion of inflammation. This effect constitutes the third observation included in our mathematical model. Finally, while profibrotic stimulation by IL-4 promoted in Mφ the alternative activation phenotype, which was not significantly influenced by neither the direct contact with Fb nor the presence of hypoxia, Fb conditioned with IL-4 did not respond in hypoxia, neither when in contact with Mφ. However, IL-4 conditioned Fb differed from the resting ones, which changed their phenotype in hypoxic environment when in contact with Mφ. These results suggest that IL-4 per se has no impact on Fb, but through conditioning of Mφ it induces an inhibitory mechanism preventing Fb from acquiring a proinflammatory phenotype. Indeed, we can suppose that though IL-4 is not sufficient to induce the acquisition of profibrotic properties in Fb, through Mφ it is able to influence Fb and their response to hypoxia.

Taken together, these observations obtained in an integrated tissue-like context candidate hypoxia, inflammation, and the Th2 cytokine IL-4 as key regulators of fibrosis. Consistent with this, the in vitro signatures generated in our in vitro model were able to describe up to 50% of the ex vivo signatures generated from laser microdissected fibrotic transplant nephrectomies, a surprisingly high percentage considering the variety of cell types and factors acting in the tissue that were not taken into account in the model. Moreover, we were able to distinguish different signatures, suggesting enrichment for variable functions, in three distinct anatomical regions of kidneys: interstitium, glomeruli surrounding area, and the compartment of glomeruli themselves. A recent work by Barwinska et al. has reported a 15-gene signature list characterizing different kidney anatomical regions^42^. We observed a good overlap of these signatures with our results on healthy tissues, which validated all the 15 markers specifically associated with glomeruli and 7 out of the 15 markers, likely due to higher complexity in delineating this second region. Specifically, our approach of micro-dissecting a fixed radius around glomeruli results in bulk signatures composed of epithelial and interstitial elements, which can be expected to be distinct from profiling the tubular structures separately. In contrast to the considerable overlap in healthy samples, the transcriptomic profile associated with the IF/TA interstitium of our kidney transplant rejection cases showed only a limited overlap (4.9%) with the IF/TA interstitium of diabetic patients reported by Barwinska et al., indicating that IF/TA can be driven by fundamentally different pathological processes in different clinical conditions. Unfortunately, we could not evaluate whether this also applies to glomeruli as this anatomical compartment has not been molecularly characterized in the diabetic patients study.

A prominent proinflammatory feature of the interstitial compartment, with immune response and leukocyte activation, was already evident from the GO analysis performed on the transcriptome of the nephrectomy ex vivo samples. In addition, the in vitro signatures of Mφ cocultivated with Fb in hypoxic and proinflammatory conditions shared the proinflammatory features as major source of variation and were able to explain 2.7% of differentially expressed genes in this anatomical region univocally. Moreover, interstitium showed a higher density of Mφ, T and B lymphocytes than the other regions suggesting a massive inflammatory response in this area. In the glomeruli surrounding region, inflammation remained an important feature, similarly but at lower levels than in the interstitium, but in addition the transcriptomic profile of this compartment showed also a slightly increased relevance of processes linked to ECM remodeling. This corresponded to enrichment of transcriptomic signatures connected to Mφ IL-4 stimulation that were obtained by in vitro experiments under hypoxic conditions with Fb coculture. Finally, the transcriptome of the glomerular compartment showed a strikingly different composition: in these areas we found predominant signatures of Fb and enrichment for gene sets clearly suggesting profibrotic behavior with a reduction of proinflammatory properties. The trend towards gradually declining levels of inflammation from high in the interstitium, lower in glomeruli surrounding area, and lowest in glomeruli was paralleled by an opposite for signatures of fibrosis for which enrichment was least prominent in the interstitium, increased in the glomeruli surrounding area and was highest in the glomeruli. Corresponding to the transcriptomic profiles, IHC showed in some glomeruli aggregates of FAP^+^ activated Fb in largely isolated localization, suggesting regions of “cold” fibrosis as proposed by ref. 25, where no direct interaction with Mφ or other immune cells is required to maintain a profibrotic state. Moreover, the same microscopic section of renal tissue could concomitantly include glomeruli with different density of FAP^+^ cells, and different degrees of fibrosis. This spatial heterogeneity of the extent of fibrosis and profibrotic states within a kidney can be predicted, based on the mathematical model that we have developed on the basis of in vitro observations. Given that the model predicts the existence of three dynamic regimes that can modify the interplay between Mφ and Fb depending on local hypoxia and inflammation, it can be expected that the regional outcome in a kidney with its complex vascular system, potential pre-existing pathological conditions and variable presence of Mφ and Fb due to locally different of focal inflammatory infiltrates will be heterogeneous. The predicted distinct cellular trajectories, which could lead to different degrees of stability ranging from stable healthy to irreversible pathological conditions, is reflected by immunohistological data confirming spatial heterogeneity with the co-existence of hot and cold fibrosis regions. Though based on a single case, the analysis of renal biopsies from a SV40-positive case with reactivation of the polyomavirus BK suggests that the mathematical prediction is not necessarily specific for graft rejection.

Taken together, the findings confirm that Fb and Mφ are central to the development of fibrosis, and show that additional local microenvironmental cues such as inflammation, Mφ polarization, and hypoxia have relevant impact on the regional outcome of profibrotic constellations. Consequently, therapeutic interventions in order to avoid or reduce fibrosis may have variable effects on the delicate balance between different factors, including oxygenation, the degree and type of inflammation, and local anatomical conditions in different kidney compartments. This may have some intriguing therapeutic implications. On a first note, our results confirm the plausibility of anti-inflammatory drug usage, which is the major strategy in the current Standard of Care. However, the model-based predictions reveal that a critical inflammation intensity highly depends on local hypoxia and cell interactions, which may explain why anti-inflammatory treatments may fail to fully prevent fibrosis. Moreover, interventions controlling Fb proliferation, for example, PDGFR-targeted drugs such as Nintedanib, may be helpful against fibrosis development. However, the inhibitory effect on Fb proliferation rates and the overall outcome may again depend on local inflammation and hypoxia, implying that multimodal approaches optimizing immunological and metabolic cues may be the most promising application of such drugs. The same holds true for drugs modifying Mφ reprogramming, which could potentially contribute to effective treatment strategies, but only under certain microenvironmental circumstances. For example, the present model would suggest that this could only be successful when Mφ phenotypic switch rate (λ) is larger than the Fb viability rate β/δ2 (proliferation/death). Overall, therapeutic interventions avoiding or significantly reducing development of fibrosis in anatomically complex organs like the kidney are unlikely to be successful in the setting of unimodal treatment.

Results reported here indicate that combining in vitro/ex vivo transcriptomics and in silico modeling is a powerful approach to dissect molecular and cellular mechanisms underlying complex biological processes and possibly support the discovery and development of new therapeutic strategies. It is equally important however to define the main limitations of this approach. First, the restricted availability of biopsy-based clinical data largely limited the parametrization of cell kinetic to human in vivo cell dynamics. On the other hand, calibrating the model to in vitro parameters would have had little relevance to any clinical scenario. Therefore, we chose an alternative route where we explored model dynamics for large biologically relevant parametric regimes. In turn, we focused on the robustness of the three identified dynamic fibrotic states under parametric variations (see Figs. S1a, S9, S10). Second, the model indirectly included the impact of main immunological cell populations, such as T cells. Although their lump effect has been taken into account by the inflammation parameter (y), the full complexity of immune interactions is not yet reflected and could confer further interesting implications. This is a limitation, but it also suggests a clear path for extending the model. Third, our model involved only mean-field non-spatial dynamics. The importance of space/anatomy has been exemplified in the analysis of different tissue specimens in terms of immune constitution and transcriptomic profile. In our model each tissue district would correspond to a different inflammation value (y) and hypoxia dynamics (H). This could not be realized within the given limitations in resources and represents a promising extension of this model. Fourth, although including general aspects of fibrosis, the clinical use case for this model is specifically limited to ex vivo findings related to IF/TA progression and glomerulosclerosis in transplanted kidneys. We present this as a relevant clinical example, as any transplantation inevitably involves severe hypoxia (e.g., the cold ischemia during surgery and transport) and inflammation through rejection (except genetically identical twin transplantation). The qualitative relevance of the model itself may be broader than this clinical use case, as it has been developed based on in vitro data generated without any reference to a specific tissue. Nevertheless, its application to distinct diseases will require further consideration of specific pathogenic mechanisms.

## Methods

### Cell cultures

Human monocytes were obtained from healthy blood donor buffy coats, upon approval by the local ethical committee. Monocytes were isolated by two-step density gradient centrifugations using Lympholyte H (Cederlane) and 46% Percoll (Lonza) followed by incubation of purified cells in RPMI 1640 (Lonza) without serum, for 20 min at RT. Adherent monocytes were washed twice with PBS and then cultured in RPMI medium supplemented with 10% fetal bovine serum (FBS; Lonza), 100 U/mL penicillin/streptomycin (Lonza), and 2 mM L-glutamine (Lonza). Monocyte purity was >90% as assessed by CD14/CD16 FACS analysis^43^. Mφ were obtained by culturing monocytes for 7 days in complete RPMI supplemented with human M-CSF (100 ng/ml; Miltenyi). The human dermal BJ fibroblast cell line (CRL­2522; ATCC) was cultivated in high glucose D-MEM (Lonza) 10% FBS, 100 U/mL penicillin/streptomycin, and 2 mM L-glutamine. When cultivated in normoxic conditions, Mφ and Fb were maintained at 37 °C in a humidified incubator settled at 20% O_2_, 5% CO_2_ in air, while hypoxic treatment was performed moving cells at 37 °C in a humidified incubator with a mixture of 1% O_2_, 5% CO_2_ and 94% N_2_. Hypoxic and normoxic cells are labeled “H” and “N”, respectively. Mφ were polarized toward a proinflammatory phenotype (MI) by incubation with 100 ng/ml LPS (Sigma) plus 20 ng/ml IFNγ (R&D Systems) or into an alternative phenotype (MF) by incubation with 20 ng/ml IL-4 (Miltenyi)^44^. Resting Mφ (M0) were left unstimulated for the same period. Fb were stimulated as Mφ, with cells treated with LPS + IFNγ (FbI), IL-4 (FbF) or left unstimulated (Fb0). Polarizing stimuli and hypoxia were applied simultaneously. For coculture experiments, differentiated Mφ were replated directly onto adherent Fb (plated 16 h before) with a 2:1 ratio. After 24 h of coculture in basal conditions (normoxia without stimuli), cells were stimulated as described above, detached, and FACS sorted (FACS Aria III; BD Bioscience) based on staining with anti-human CD45 clone 2D1 (dilution 1:1000; Cat.No: 560178; BD Bioscience) to distinguish CD45^+^ Mφ from CD45^−^ Fb using a FACS Aria III cell sorter (BD Bioscience). Zombie Aqua Fixable Viability kit (Cat.No: 423101; BioLegend) was used to exclude dead cells (L/D^+^) (see Fig. S1c for the gating strategy and Source Data file for sorted cell numbers). Single cell and FACS-sorted cocultivated cells are labeled “SC” and “CC”, respectively. The combination of different parameters applied to Mφ and Fb generated a total of 44 different experimental conditions, detailed in Fig. S1b. The experiment was performed in triplicate and the transcriptomic profiles were then investigated.

### Histological samples

Immune cell phenotyping and transcriptional analysis were performed on formalin-fixed paraffin-embedded 3 µm thick consecutive sections from archival material of four transplant nephrectomy specimens (Cases A-D, see Supplementary Table 1) that were selected to represent different stages and underlying causes of fibrosis. The cases include three explanted kidneys that lost function due to previous TCMR and/or ABMR, and one nephrectomy specimen surgically removed because of the development of renal cell carcinoma within the transplanted organ^27^. As non-fibrotic control samples we used tissue selected as distant as possible from the tumor margin form four tumor nephrectomy specimens. Protocol biopsies and indicated biopsies from kidney transplants were obtained from a previous study^28^, and the clinical follow-up (development of GFR over time) was obtained in the context of the SYSIMIT systems medicine study (www.sysim.it). The study was approved by the local institutional review board (IRB) the *Ethikkommission* (Ethics Commission) of Hannover Medical School; approval number #2063-2013 and its amendment #2968-2015. Additional ethical approval was obtained according to the guideline for ERACoSysMed- funded translational projects, including *Comité de Protection des Personnes*, Est-IV (Ethical Research Committee), Strasbourg, France and the *Comitato Etico Indipendente* (Independent Ethics Committee) of IRCCS, Milan, Italia. The approvals cover (1) research use of surplus archivial material from nephrectomy and indicated biopsy samples after completion of the diagnostic workup and associated anonymized (non-identifiable) clinical information by a waiver for individual informed consent, and (2) research use of surplus archivial biopsy material and associated pseudonymised clinical information of patients who gave their written informed consent when entering the Hannover Medical School protocol biopsy registry program. Sections were stored at 4 °C in the dark to minimize antigen aging over time until further processing.

### Immunohistochemistry (IHC)

Standard histology (H&E, PAS, sirius red) and IHC to detect (fibroblast-activating protein FAP, CD206) as well as control experiments to calibrate the multiplexing IHC for CD4, CD8, CD20, CD68, CD206, MS4A4A, and FAP) were performed using an automated staining instrument (Ventana Benchmark Ultra) following the manufacturer’s recommendations, and using 3,3 diaminobenzidineor Fast-Red as chromogens. Primary antibodies are listed below, except polyclonal anti-MS4A4A (Sigma Life Science, 1:200, #HPA029323), and anti-SV40 (clone MRQ-4; Cell Marque, 1:750,#351M-16). Multiplexed IHC analysis was optimized following the manufacturer’s instructions (OPAL Multiplex IHC Assay Development Guide, Akoya Bioscience). 2–4 validation experiments per IHC assay were performed on subsequent nearly consecutive sections with identical primary antibodies in automated chromogenic single or duplex immunohistochemical staining experiments, confirming identical staining patterns in comparison with single-channel view after multiplexed immunohistochemistry. Some of these multiple stainings on sequential tissue sections were used for robustness testing and development of image analysis algorithms [ref. 27]. Slides were deparaffinized, initial antigen retrieval was performed by microwave cooking in Tris-buffered saline (TBS) at pH 9, and blocking of unspecific protein binding was performed using Protein Block Serum-free solution (Agilent/Dako). Subsequent antigen retrieval and deactivation of the preceding staining step was performed either in TBS at pH 9 or citrate buffer at pH 6. Consecutive IHC staining using the OPAL 7-plex fluorescence system was performed using the following primary antibodies diluted in REAL Antibody Diluent Agilent/Dako #S2022: anti-CD4 (clone SP35; Zytomed Systems, 1:50, #503–3354), anti-CD8 (clone C8/144B; Agilent/Dako, 1:350, #M7103), anti-CD20 (clone L26; Dako, 1:500, #M0755), anti-CD68 (clone PG-M1; Dako, 1:1000, #M0876), anti-CD206 (clone 5C11; Bio-Rad, 1:1500, #MCA5552Z), anti-FAP (clone D8; Vitatex, #MABS1001, 1:1500). Cell nuclei were stained with DAPI. The following fluorophores were used in the tyramide signal amplification-based multiplexed system to detect bound antibodies: Opal 520, Opal 540, Opal 570, Opal 620, Opal 650 or Opal 690. Fluoromount-G mounting medium (Thermo Fisher Scientific) was applied to cover slides before imaging.

### Multispectral image analysis and quantitative evaluation

Whole slide image scanning was done at 20x magnification using the Vectra Polaris instrument (Akoya Bioscience). Three-channel fluorescent whole slide images were used to select regions of interest in nephrectomies (466 × 349 µm size) for subsequent targeted scanning of image stacks at 40x magnification across the visible spectrum (420–720 nm) for multispectral imaging, containing glomeruli and the surrounding region, or representative areas of the interstitial compartment. Biopsies were fully scanned and spectral libraries were generated using single stained scans of tonsil tissue for each reagent. Deconvolution and training for the machine learning-based segmentation, tissue classification and cell phenotyping algorithms were performed using the inForm v2.4.8 software (Akoya Bioscience). Visual quality control of results was performed by comparing all composite images and selected single-channel images and phenotyping results with corresponding chromogenic single and duplex staining of adjacent consecutive sections.

### Cell density and neighborhood analysis

Cell phenotypes and individual coordinates of each cell were exported in text (.csv) format and further processed in GraphPad and MS Excel (Source data is provided as Source data file). In the context of neighborhood analysis the term “glomerulus” refers to the area within the Bowman membrane. For this analysis, the term “surrounding” refers to the area within a ROI (see above) of 466 × 349 µm size, where the glomerulus is in the center (Fig. 6a–c, panels labeled with “Glo”), subtracting the glomerulus area from each ROI. The term “interstitium” refers to ROIs of the same 466 × 349 µm size that are taken exclusively in interstitial areas, strictly avoiding areas meeting the “glomerulus” or “surrounding” criteria.

### Laser-captured microdissection (LCM) and RNA sequencing

Formalin-fixed paraffin-embedded 5 μm thick tissue sections adjacent to the sectioning levels used for multiplexed IHC were mounted on a poly‐l‐lysin‐coated membrane attached to a metal frame under RNAse-free conditions. LCM was performed on deparaffinized, hemalaun-stained sections using the Cell Cut Plus System (MMI Molecular Machines & Industries). In the context of LCM analysis, the term “glomerulus” refers to the area within the Bowman capsule; the term “surrounding” refers to a circle with a diameter of 550 µm with the glomerulus in the center, obtained after the glomerulus had been removed, and considering that in paraffin sections glomeruli show a diameter of ∼200 µm (range 150–250 µm) and some tissue loss caused by the laser beam, in practical terms this area corresponds to a ring area of 200–250 µm around each glomerulus adopted for the cell density and neighborhood analysis; the term “interstitial” refers to areas of variable shape selected by strictly avoiding the other two compartments (Fig. S7). We chose this pragmatic terminology to make the LMD and multispectral image analysis results comparable using similar distances/areas. RNA isolation was performed using the RNeasy Mini Kit (Qiagen) in combination with Qiashredder columns (Qiagen) according to the manufacturer’s instructions. Obtained RNA quality was checked using the RNA Nano Kit (Agilent Technologies) on an Agilent BioAnalyzer 2100 (Agilent Technologies). Prior to library generation, nucleic acid samples were pretreated with DNase (Qiagen). 5 ng equivalents from DNase pretreated samples were used for library preparation with the SMARTer Stranded Total RNA-Seq Kit – Pico Input Mammalian (#635006; Takara/Clontech) according to conditions recommended in user manual #101215. Generated libraries were barcoded by dual indexing approach and were finally amplified with 15 cycles of PCR. Fragment length distribution of generated libraries was monitored using a BioAnalyzer High Sensitivity DNA assay (5067–4626; Agilent Technologies). Quantification of libraries was performed by use of the Qubit® dsDNA HS Assay Kit (Q32854; Thermo Fisher Scientific). Pooled libraries were denatured with NaOH and finally diluted to 1.8pM according to the Denature and Dilute Libraries Guide (document # 15048776 v02; Illumina). 1.3 ml of denatured pool was loaded on an Illumina NextSeq 550 sequencer using a High Output Flowcell for 75 bp single reads (#FC-404-2005; Illumina). BCL files were converted to FASTQ files using bcl2fastq Conversion Software version v2.20.0.422 (Illumina). Raw data processing and quality control were conducted by use of nfcore/rnaseq (version 1.5dev). The genome reference and annotation data were taken from GENCODE.org (*Homo sapiens*; GRCh38.p12; release 28). Normalization and differential expression analyses were performed with DESeq2 (Galaxy Tool Version 2.11.40.2) with default settings except for “Output normalized counts table” which was set to “Yes”. FACS sorted samples were collected and cells were lysed by TRIzol reagent. Total RNA was isolated using DirectZOL RNA miniprep kit (ZymoResearch) according to the manufacturer’s instructions. Quantification and quality check (RNA integrity number RIN > 7) were assessed by using Qubit4 (Invitrogen) instrument. Libraries preparation and processing were performed with Lexogen protocol, using the QuantSeq 3’ mRNA-Seq Library Prep kit to generate Illumina-compatible libraries of sequences close to the 3’-end of poly(A) RNA. Sequencing was performed using a NextSeq 500 (Illumina), producing an average of 5 × 10^6^ reads/sample (single-end, 75 bp).

### Bioinformatics analysis of transcriptomic data generated on the in vitro model

Reads from RNA-sequencing were subjected to quality check and trimming using the FastqQC and BBduk tools and to alignment using the STAR method. The Phread quality score was greater than 20, and the percentage of alignment along the reference genome was higher than 80% along all the samples. Reads were aligned along genes using the HTseq count tool. Counts matrix was normalized with the TMM method (EdgeR 3.24.3). Gene expression TMM matrix counted a total of 17,650 genes for each of the 132 samples. Principal Component Analysis (PCA) was performed using Variance Stabilizing Transformation (VST, DESeq2 1.22.2) on all genes under investigation. The single sample GSEA was performed starting from the normalized counts matrix with Hallmark collection gene set. For this analysis, a new gene expression matrix was defined, considering the mean expression of replicates (matrix 17.650 × 44). In the ssGSEA algorithm, averaged replicates were considered one by one. A score for each signature for each sample was calculated determining an absolute values score matrix (signature x samples). The score matrix was normalized considering the absolute value of the maximum value and the absolute value of the minimum one. The normalized matrix was plotted in a heatmap. The relative contribution of each variable and multiple combined effects was evaluated by supervised analysis at three levels of increasing complexity, as reported in the Supplementary methods section.

### Bioinformatics analysis of transcriptomic data generated on ex vivo samples

Ex vivo data were analyzed with the same protocol by comparing pathological and control samples. Signatures generated by in vitro and ex vivo datasets were compared by using a multi-step method that we have developed. Pathway enrichment in the pathological samples was analyzed by Metascape online tool^45^, considering DEGs in nephrectomy ex vivo data (logFC ≥ 1 and *p* ≤ 0.01). Only pathways with a –log_10_ (*p*-value) that exceeds the 1.3 threshold value were selected and represented by bar plots. Pre-ranked GSEA were analyzed by ranking ex vivo data on logFC, using in vitro data as input gene set signature. For each differential analysis a signature was obtained and the overlap between ex vivo and in vitro data was tested. Only in vitro signatures significantly enriched and overlapped with ex vivo signature were selected. See the Supplementary methods section for a detailed description of pre-ranked GSEA and gene overlap analysis.

### Mathematical modeling

An ordinary differential equation model was constructed to describe the dynamics of activated Mφ and fibroblasts and analyze the corresponding qualitative behavior in potential in vivo scenarios. The equations consist of terms related to the in vitro experimental observations and biological assumptions:

A1. Proinflammatory Mφ are recruited proportionally to the inflammation level.

A2. Profibrotic Mφ proliferate in the presence of Fb, in hypoxic and proinflammatory environments, while in normoxic environments, they become senescent (A, B).

A3. Fb become activated and promote cell growth in the presence of Mφ in hypoxic conditions.

A4. All cell types become deactivated at a constant rate.

A5. Due to the short phenotypic switch rates compared to the system’s characteristic time scale (inverse Mφ deactivation rate), Mφ were assumed to be in equilibrium with respect to their phenotypic switch.

Under these assumptions, we define the following system of ordinary differential equations:

1 $$\dot{{M}_{1}}={lY}+{E}_{1,2}-{d}_{1}{M}_{1},$$

2 $$\dot{{M}_{2}}={rH}\frac{{M}_{2}F\left(Y+{Y}_{0}\right)}{{M}_{2}+K}-{E}_{1,2}-{d}_{2}{M}_{2},$$

3 $$\dot{F}={gH}{M}_{2}F\left({F}_{0}-F\right)-{d}_{3}F,$$

where ${M}_{1}$ represents the number of proinflammatory Mφ, ${M}_{2}$ the number of profibrotic Mφ, $F$ the number of activated Fb, $Y$ the inflammation level, $H$ the hypoxia level, $l$ the level of macrophage recruitment due to local inflammation, ${d}_{1}$ the death rate of proinflammatory Mφ, $r$ the profibrotic macrophage proliferation rate, ${Y}_{0}$ a reference level of inflammation, $K$ the base inhibition of profibrotic macrophage proliferation, ${d}_{2}$ the death rate of profibrotic Mφ, $g$ the activation rate of Fb, ${F}_{0}$ the mean maximum number of Fb in fibrosis, and ${d}_{3}$ the death rate of Fb. Phenotypic switch between profibrotic and proinflammatory Mφ was considered via the switching term:

4 $${E}_{1,2}=-{k}_{1\to 2}{M}_{1}+{k}_{2\to 1}{M}_{2}Y,$$

where ${{k}}_{1\to 2}$ and ${k}_{2\to 1}$ are the switching rates from proinflammatory to profibrotic phenotypes and vice versa.

Due to the short phenotypic switch rates compared to the rates of cell proliferation and deactivation, Mφ were assumed to be in equilibrium with respect to phenotypic switch. Assumption A5 allow us to link the number of profibrotic and proinflammatory Mφ as:

5 $$Y={\lambda }^{-1}\frac{{M}_{1}}{{M}_{2}},$$

where $\lambda=\frac{{k}_{2\to 1}}{{k}_{1\to 2}}$ is the ratio of the switching rates. Using this expression, we can combine the equations for both macrophage phenotypes into a single equation for the total macrophage number, $M={M}_{1}+{M}_{2}.$ The model is simplified further by non-dimensionalizing all quantities (Box 1). The fully parameterized model has been deposited in the Biomodels database (MODEL2209160001).

When the cytokines PDGF and CSF1 are assumed to be in a quasi-steady state, the reduced model (Box 1) has a similar structure to the one developed by ref. 25. For a comparison of the two models refer to the mathematical modeling appendix in the Supplementary information section. In contrast to ref. 25, the activation rates of the two cell populations are modified by the impact of inflammation and hypoxia. Under normoxic conditions ($H=0$), there is a single stable steady state:

6 $$m=\frac{y\left(1+\lambda y\right)}{\lambda y+{\delta }_{1}},$$

7 $$f=0.$$

In hypoxia, this steady state exists along with two additional steady states. Linear stability analysis reveals that the steady state common to hypoxic and normoxic conditions (the “healthy” steady state) is a stable node as long as:

8 $$H \, < \, \frac{{\delta }_{2}}{\beta }\left(\lambda+\frac{{\delta }_{1}}{y}\right),$$

or written in terms of the inflammation level, which defines a critical inflammation, above which the system cannot return to the healthy state:

9 $$y \ < \ {y}_{{{\mbox{cr}}}}=\frac{{\delta }_{1}}{\frac{\beta }{{\delta }_{2}}H-\lambda }.$$

It should be noted that, in the limit of very high inflammation (${\delta }_{1}\ \ll \ \lambda$), stability of the healthy state is still possible, as the stability condition reduces to:

10 $$H \, < \, \frac{\lambda {\delta }_{2}}{\beta }.$$

One of the two steady states found only in hypoxic conditions is numerically found to correspond to a saddle point, while the other can be a stable or unstable node (“fibrotic” steady state) with $f\approx 1$ and $m\,\gg\, 1$. The inflammation-hypoxia space can be partitioned into three regions depending on the stability of the two nodes: (I) the healthy state is stable, the fibrotic state is unstable; (II) both states are stable; (III) the healthy steady state is unstable, the fibrotic state is stable (Fig. 10b).

Box 1 Mathematical model The mathematical model describes the change in total number of macrophages and fibroblasts through the following system of coupled ordinary differential equations:

$$\dot{m}=y-m\frac{\lambda y+{\delta }_{1}}{1+\lambda y}+\alpha H\frac{\left(y+1\right)m}{m+1+\lambda y}f,$$

$$\dot{f}=\beta H\frac{m}{1+\lambda y}f\left(1-f\right)-{\delta }_{2}f,$$

where $m$ and $f$ are the non-dimensional macrophage and fibroblast populations, respectively, $y$ represents the non-dimensional inflammation, ${\delta }_{1}$ is related to the ratio of profibrotic to proinflammatory macrophage deactivation rates, ${\delta }_{2}$ is the ratio of fibroblast to proinflammatory deactivation rates, $\alpha$ is proportional to the profibrotic macrophage proliferation rate, $\beta$ is proportional to the fibroblast proliferation rate, $H$ is the hypoxia level, and $\lambda$ is the ratio of macrophage switching rates from proinflammatory to profibrotic phenotypes and vice versa. All quantities are non-dimensional.

### Statistical analysis

Statistical analysis was performed using Prism version 7.0 (GraphPad software). Comparisons were calculated by two-way ANOVA test applying Tukey’s multiple comparisons correction. The level of statistically significant difference was defined as *p* < 0.05.

### Reporting summary

Further information on research design is available in the Nature Research Reporting Summary linked to this article.

## Supplementary information

Supplementary Information

Description of Additional Supplementary Files

Supplementary Data 1

Supplementary Data 2

Supplementary Data 3

Supplementary Data 4

Reporting Summary

## Data Availability

The RNA-sequencing data generated in this study and raw data for Fig. 6h–j have been deposited in the Zenodo database (https://zenodo.org/record/7016644#.YwSzUhxByN4). 10.5281/zenodo.7016644. The data supporting the findings of this study are provided in the Supplementary Information/Source Data file. Source data are provided with this paper.

## Source data

Source Data

## Code availability

All codes used in this study are consistent with the “best practice workflow” suggested by the developers of each used tool as described in Methods section. RNA-sequencing data were analyzed with the same pipeline, based on described software. Heatmaps and plots were generated with standard parameters for each of the described tools. Codes are deposited in github (github.com/AlessandraCastagna/RNASeq_NatureComm2022). Mathematical code: the “fully parameterized” model has been deposited in BioModels^46^ and assigned the identifier MODEL2209160001. The code used to perform neighborhood analysis is provided in the Source Data file.
