# Supplementary material for: Understanding fibrosis pathogenesis via modeling macrophage-fibroblast interplay in immune-metabolic context
Source: Nat Commun. 2022 Oct 30;13:6499. doi: 10.1038/s41467-022-34241-5 (PMC9618579; doi:10.1038/s41467-022-34241-5)
Supplement: Supplementary file 1 — Supplementary Information [file 41467_2022_34241_MOESM1_ESM.pdf]

Supplementary information

Understanding fibrosis pathogenesis via modelling macrophage-fibroblast interplay in immune-metabolic context

Elisa Setten[1,2], Alessandra Castagna[1], Josué Manik Nava-Sedeño[3], Jonathan Weber[4], Roberta Carriero[1], Andreas Reppas[5], Valery Volk[6], Jessica Schmitz[6], Wilfried Gwinner[7], Haralampos Hatzikirou[8,9,10§], Friedrich Feuerhake[6,11§], Massimo Locati[1,2,§ *]

[1]IRCCS Humanitas Research Hospital, Rozzano, Italy; [2]Department of Medical Biotechnologies and Translational Medicine, Università degli Studi di Milano, Milan, Italy; [3]Mathematics Department, National Autonomous University of Mexico, Mexico City, Mexico; [4]IRIMAS Institute, Université de Haute-Alsace, Mulhouse, France; [5]Charité-Universitäts medizin Berlin, corporate member of Freie Universität Berlin, Humboldt-Universität, Berlin, Germany; [6]Institute for Pathology, Hannover Medical School, Hannover, Germany; [7]Department of Nephrology, Hannover Medical School, Hannover, Germany; [8]Mathematics Department, Khalifa University, PO Box 127788, Abu Dhabi, United Arab Emirates; [9]Technische Univesität Dresden, Center for Information Services and High Performance Computing, Nöthnitzer Straße 46, 01062, Dresden, Germany; [10]Healthcare Engineering Innovation Center (HEIC), Khalifa University of Science and Technology, Abu Dhabi, UAE; [11]Institute for Neuropathology, University Clinic Freiburg, Freiburg, Germany.

[§] Equally Contributing Authors

*** Corresponding author:**
Locati Massimo, MD
Humanitas Research Hospital
Via Manzoni 56; I-20089 Rozzano, Italy
Tel: +39-(0)2.8224.5116; E-mail: massimo.locati@humanitasresearch.it

Supplementary figures

a

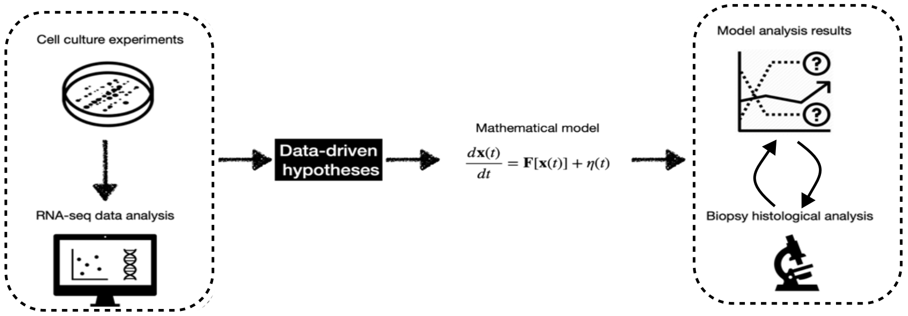

b

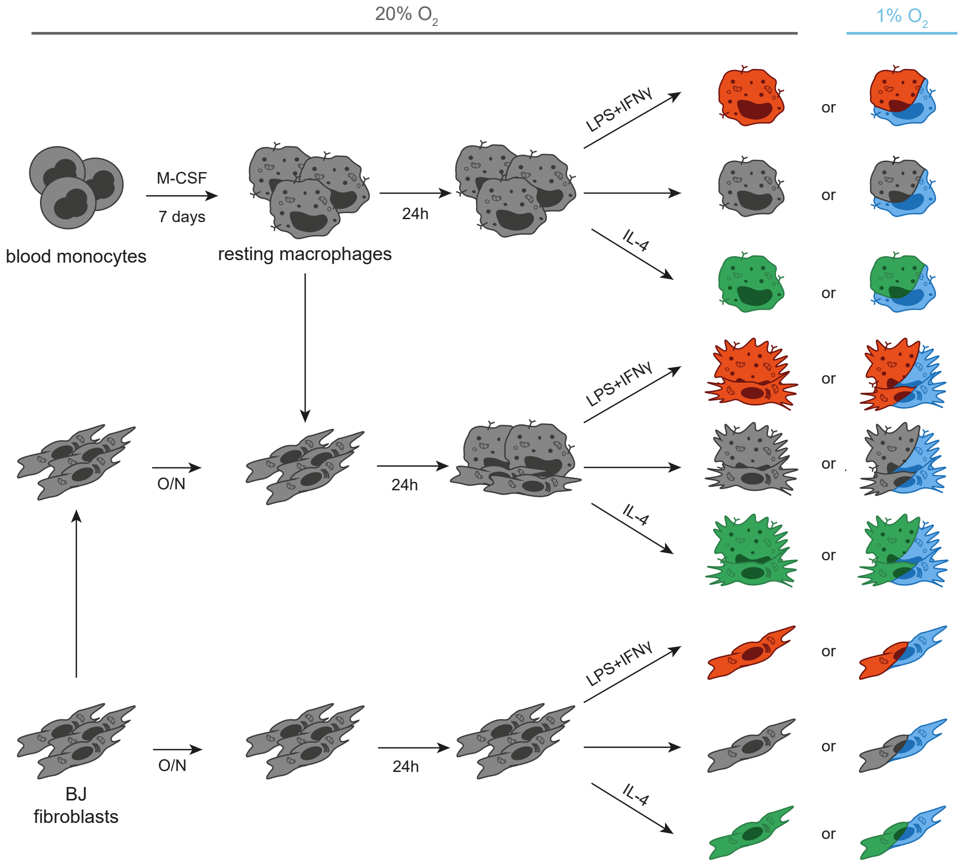

	single culture (SC)					
	M0	MI (LPS+IFNγ)	MF (IL-4)	Fb0	FbI (LPS+IFNγ)	FbF (IL-4)
normoxia 4h (4N)		MI SC 4N	MF SC 4N		FbI SC 4N	FbF SC 4N
hypoxia 4h (4H)	M0 SC 4H	MI SC 4H	MF SC 4H	Fb0 SC 4H	FbI SC 4H	FbF SC 4H
normoxia 24h (24N)	M0 SC 24N	MI SC 24N	MF SC 24N	Fb0 SC 24N	FbI SC 24N	FbF SC 24N
hypoxia 24h (24H)	M0 SC 24H	MI SC 24H	MF SC 24H	Fb0 SC 24H	FbI SC 24H	FbF SC 24H
	coculture (CC)					
	M0	MI (LPS+IFNγ)	MF (IL-4)	Fb0	FbI (LPS+IFNγ)	FbF (IL-4)
normoxia 4h (4N)		MI CC 4N	MF CC 4N		FbI CC 4N	FbF CC 4N
hypoxia 4h (4H)	M0 CC 4H	MI CC 4H	MF CC 4H	Fb0 CC 4H	FbI CC 4H	FbF CC 4H
normoxia 24h (24N)	M0 CC 24N	MI CC 24N	MF CC 24N	Fb0 CC 24N	FbI CC 24N	FbF CC 24N
hypoxia 24h (24H)	M0 CC 24H	MI CC 24H	MF CC 24H	Fb0 CC 24H	FbI CC 24H	FbF CC 24H

c

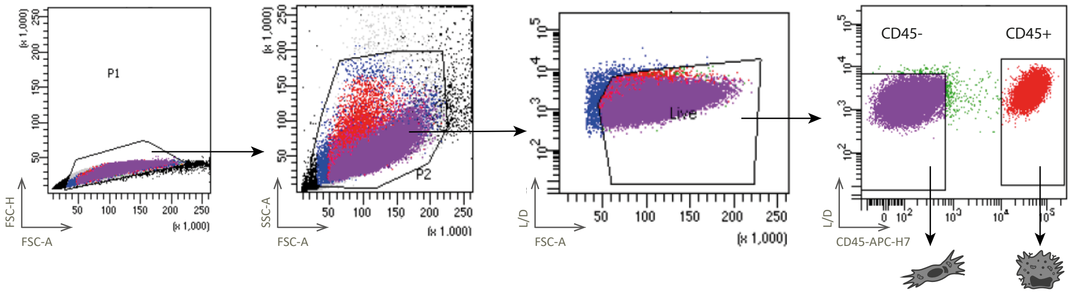

Figure S1. Experimental design and sample coding.

a) Study experimental workflow. RNA sequencing was performed on in vitro Mφ/Fb cocultures to obtain data-driven mechanistic hypothesis operating in the different experimental conditions, which informed the mathematical model that was sustained by ex vivo generated data.

b) Schematic representation of experimental conditions applied to Mφ and Fb in single culture (top and bottom lanes, respectively; round-shaped cells) and coculture conditions (middle lane; frayed cells). Resting macrophages were obtained after 7 days of differentiation from blood monocytes and plated for 24 h before starting coculture or treatment; BJ fibroblasts were plated for an overnight (O/N) plus 24h simultaneously to Mφ. Then cells were treated with LPS+IFNγ to mimic inflammation and Th1-derived immune polarization (red) or IL-4 to mimic Th2-derived immune polarization (green). Stimulations were performed in normoxic (20% O_2) or hypoxic (1% O_2) conditions (half color in light blue), and investigated at 4 and 24h. This experimental design generated the 44 different experimental conditions, coded as reported in the table, investigated by RNAseq in biological triplicates. Colors used in the figure and letter codes used in the table were maintained in all other figures of the manuscript.

c) Representative gating strategy applied on cocultivated cells after treatment. Cells were first gated to exclude doublets, on forward (FSC) and side scatter (SSC), then live cells (L/D$^-$) were sorted based on CD45 staining (sorted cell numbers are provided as a Source Data file).

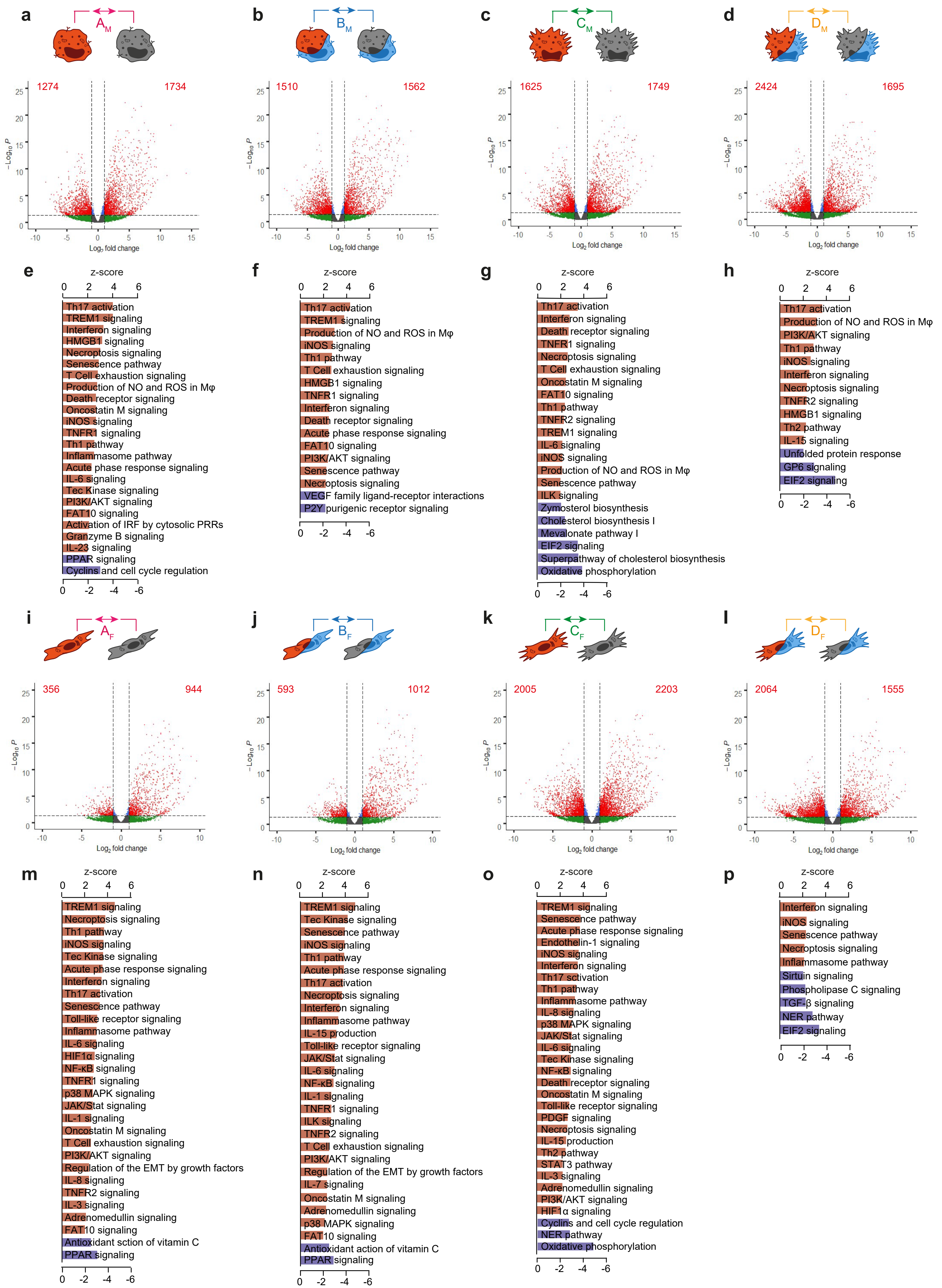
a
A_M
1274
1734
b
B_M
1510
1562
c
C_M
1625
1749
d
D_M
2424
1695
$-Log_{10}$ P
Log_2 fold change
e
z-score
Th17 activation
TREM1 signaling
Interferon signaling
HMGB1 signaling
Necroptosis signaling
Senescence pathway
T Cell exhaustion signaling
Production of NO and ROS in Mφ
Death receptor signaling
Oncostatin M signaling
iNOS signaling
TNFR1 signaling
Th1 pathway
Inflammasome pathway
Acute phase response signaling
IL-6 signaling
Tec Kinase signaling
PI3K/AKT signaling
FAT10 signaling
Activation of IRF by cytosolic PRRs
Granzyme B signaling
IL-23 signaling
PPAR signaling
Cyclins and cell cycle regulation
f
z-score
Th17 activation
TREM1 signaling
Production of NO and ROS in Mφ
iNOS signaling
Th1 pathway
T Cell exhaustion signaling
HMGB1 signaling
TNFR1 signaling
Interferon signaling
Death receptor signaling
Acute phase response signaling
FAT10 signaling
PI3K/AKT signaling
Senescence pathway
Necroptosis signaling
VEGF family ligand-receptor interactions
P2Y purigenic receptor signaling
g
z-score
Th17 activation
Interferon signaling
Death receptor signaling
TNFR1 signaling
Necroptosis signaling
T Cell exhaustion signaling
Oncostatin M signaling
FAT10 signaling
Th1 pathway
TNFR2 signaling
TREM1 signaling
IL-6 signaling
iNOS signaling
Production of NO and ROS in Mφ
Senescence pathway
ILK signaling
Zymosterol biosynthesis
Cholesterol biosynthesis I
Mevalonate pathway I
EIF2 signaling
Superpathway of cholesterol biosynthesis
Oxidative phosphorylation
h
z-score
Th17 activation
Production of NO and ROS in Mφ
PI3K/AKT signaling
Th1 pathway
iNOS signaling
Interferon signaling
Necroptosis signaling
TNFR2 signaling
HMGB1 signaling
Th2 pathway
IL-15 signaling
Unfolded protein response
GP6 signaling
EIF2 signaling
i
A_F
356
944
j
B_F
593
1012
k
C_F
2005
2203
l
D_F
2064
1555
m
z-score
TREM1 signaling
Necroptosis signaling
Th1 pathway
iNOS signaling
Tec Kinase signaling
Acute phase response signaling
Interferon signaling
Th17 activation
Senescence pathway
Toll-like receptor signaling
Inflammasome pathway
IL-6 signaling
HIF1α signaling
NF-κB signaling
TNFR1 signaling
p38 MAPK signaling
JAK/Stat signaling
IL-1 signaling
Oncostatin M signaling
T Cell exhaustion signaling
PI3K/AKT signaling
Regulation of the EMT by growth factors
IL-8 signaling
TNFR2 signaling
IL-3 signaling
Adrenomedullin signaling
FAT10 signaling
Antioxidant sction of vitamin C
PPAR signaling
n
z-score
TREM1 signaling
Tec Kinase signaling
Senescence pathway
iNOS signaling
Th1 pathway
Acute phase response signaling
Th17 activation
Necroptosis signaling
Interferon signaling
Inflammasome pathway
IL-15 production
Toll-like receptor signaling
JAK/Stat signaling
IL-6 signaling
NF-κB signaling
IL-1 signaling
TNFR1 signaling
ILK signaling
TNFR2 signaling
T Cell exhaustion signaling
PI3K/AKT signaling
Regulation of the EMT by growth factors
IL-7 signaling
Oncostatin M signaling
Adrenomedullin signaling
p38 MAPK signaling
FAT10 signaling
Antioxidant action of vitamin C
PPAR signaling
o
z-score
TREM1 signaling
Senescence pathway
Acute phase response signaling
Endothelin-1 signaling
iNOS signaling
Interferon signaling
Th17 activation
Th1 pathway
Inflammasome pathway
IL-8 signaling
p38 MAPK signaling
JAK/Stat signaling
IL-6 signaling
Tec Kinase signaling
NF-κB signaling
Death receptor signaling
Oncostatin M signaling
Toll-like receptor signaling
PDGF signaling
Necroptosis signaling
IL-15 production
Th2 pathway
STAT3 pathway
IL-3 signaling
Adrenomedullin signaling
PI3K/AKT signaling
HIF1α signaling
Cyclins and cell cycle regulation
NER pathway
Oxidative phosphorylation
p
z-score
Interferon signaling
iNOS signaling
Senescence pathway
Necroptosis signaling
Inflammasome pathway
Sirtuin signaling
Phospholipase C signaling
TGF-β signaling
NER pathway
EIF2 signaling

Figure S2. Effects of inflammation on Mφ and Fb exposed to different metabolic and culture conditions.

a-d, i-l) Volcano plots of DEGs induced by inflammatory stimuli (LPS+IFNγ) in Mφ (a-d) and Fb (i-l) when compared to resting counterparts in normoxic and single culture conditions (a, i), in hypoxic and single cultures conditions (b, j), in normoxic and cocultures conditions (c, k), in hypoxic and cocultures conditions (d, l). Number of DEGs (FDR < 0.05, threshold line: 1.3) upregulated (logFC ≥ 1) and downregulated (logFC ≤ -1) are reported in red.

e-h, m-p) Ingenuity pathway analysis identified functional pathways with a significant positive (z-score ≥ 2, in orange) or negative (z-score ≤ -2, in violet) enrichment associated to DEGs in Mφ (e-h) and Fb (m-p), under same experimental conditions reported for the corresponding volcano plot. Enriched pathways were used to generate heatmaps reported in Fig. 2e and 2f for Mφ and Fb, respectively.

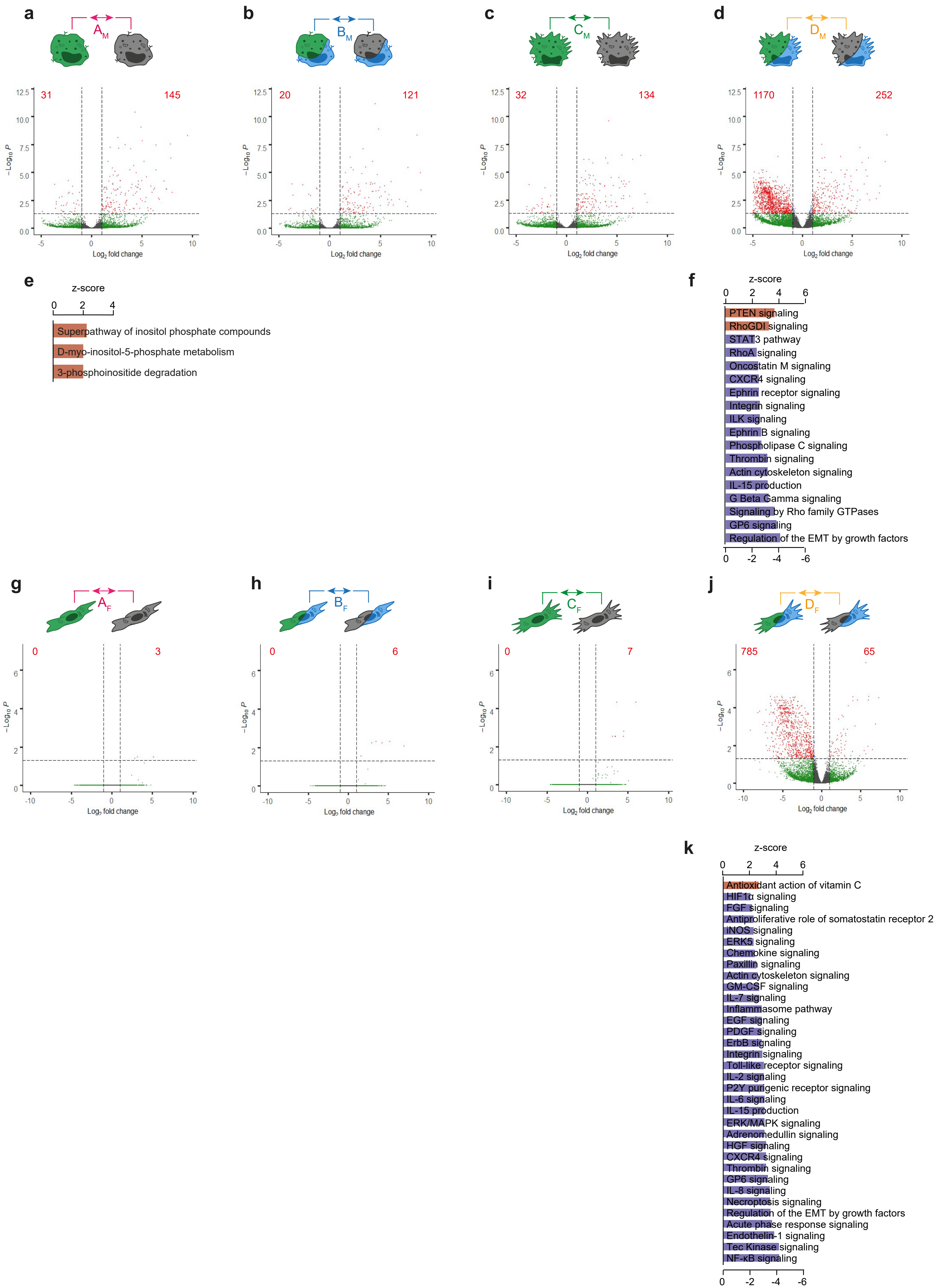
a
A_M
31
145
b
B_M
20
121
c
C_M
32
134
d
D_M
1170
252
$-Log_{10}$ P
Log$_2$ fold change
e
z-score
Superpathway of inositol phosphate compounds
D-myo-inositol-5-phosphate metabolism
3-phosphoinositide degradation
f
z-score
PTEN signaling
RhoGDI signaling
STAT3 pathway
RhoA signaling
Oncostatin M signaling
CXCR4 signaling
Ephrin receptor signaling
Integrin signaling
ILK signaling
Ephrin B signaling
Phospholipase C signaling
Thrombin signaling
Actin cytoskeleton signaling
IL-15 production
G Beta Gamma signaling
Signaling by Rho family GTPases
GP6 signaling
Regulation of the EMT by growth factors
g
A_F
0
3
h
B_F
0
6
i
C_F
0
7
j
D_F
785
65
k
z-score
Antioxidant action of vitamin C
HIF1α signaling
FGF signaling
Antiproliferative role of somatostatin receptor 2
iNOS signaling
ERK5 signaling
Chemokine signaling
Paxillin signaling
Actin cytoskeleton signaling
GM-CSF signaling
IL-7 signaling
Inflammasome pathway
EGF signaling
PDGF signaling
ErbB signaling
Integrin signaling
Toll-like receptor signaling
IL-2 signaling
P2Y purigenic receptor signaling
IL-6 signaling
IL-15 production
ERK/MAPK signaling
Adrenomedullin signaling
HGF signaling
CXCR4 signaling
Thrombin signaling
GP6 signaling
IL-8 signaling
Necroptosis signaling
Regulation of the EMT by growth factors
Acute phase response signaling
Endothelin-1 signaling
Tec Kinase signaling
NF-κB signaling

Figure S3. Effect of IL-4 on Mφ and Fb exposed to different metabolic and culture conditions.

a-d, g-j) Volcano plots of DEGs induced by IL-4 in Mφ (a-d) and fb (g-j) when compared to resting counterparts in normoxic and single culture conditions (a, g), in hypoxic and single cultures conditions (b, h), in normoxic and cocultures conditions (c, i), in hypoxic and cocultures conditions (d, j). Number of DEGs (FDR < 0.05, threshold line: 1.3) upregulated (logFC ≥ 1) and downregulated (logFC ≤ -1) are reported in red.

e, f, k) Ingenuity pathway analysis identified functional pathways with a significant positive (z-score ≥ 2, in orange) or negative (z-score ≤ -2, in violet) enrichment associated to DEGs in Mφ (e, f) and Fb (k), under same experimental conditions reported for the corresponding volcano plot. Enriched pathways were used to generate heatmaps reported in Fig. 3e and 3f for Mφ and Fb, respectively.

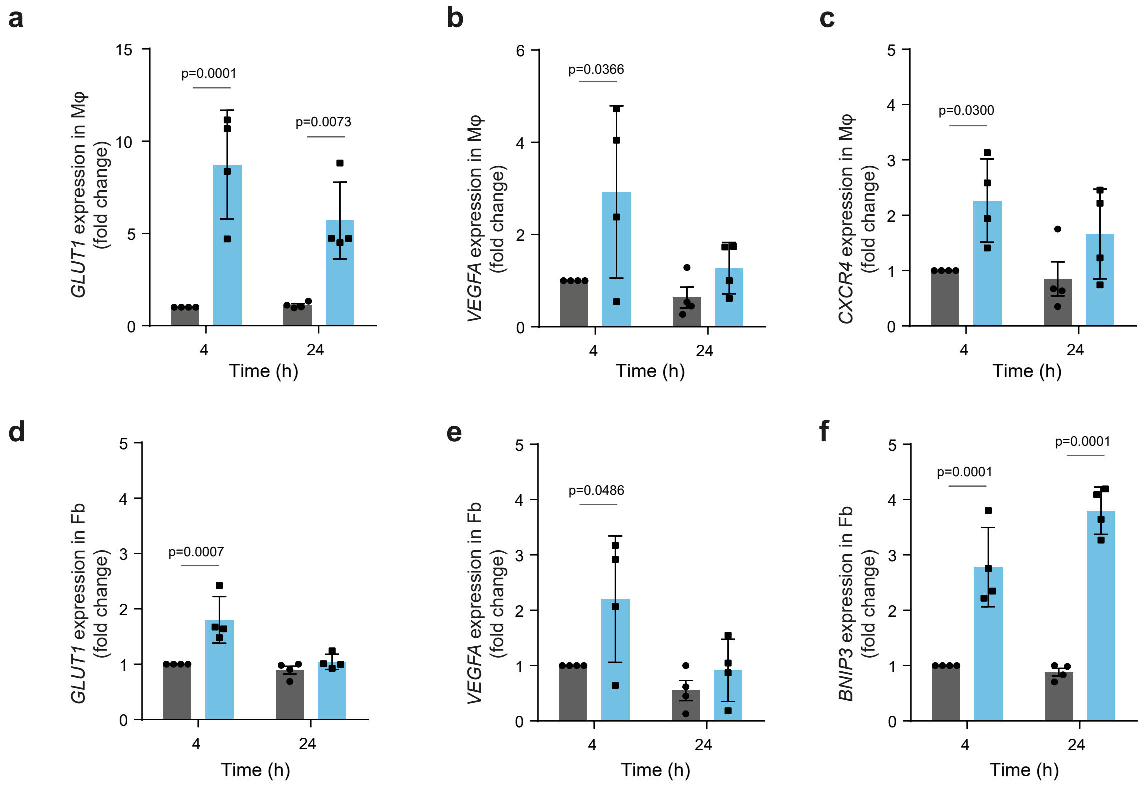

Figure S4. Transcript levels of hypoxia-responsive genes.

Hypoxia-responsive genes were investigated at different time points in single cultivated Mφ (comparison A_M; see panels a-c) and Fb (comparison A_F; see panels d-f). Transcripts abundance is reported as fold change over resting cells at 4 h. Grey and light blue bars indicate normoxia and hypoxia culture conditions, respectively. Data distribution is represented as mean of independent biological replicates (n=4) +/- SD . Comparisons are calculated by two-way ANOVA test applying Sidak's multiple comparisons correction. Significative p-values are reported on the top of the bars.

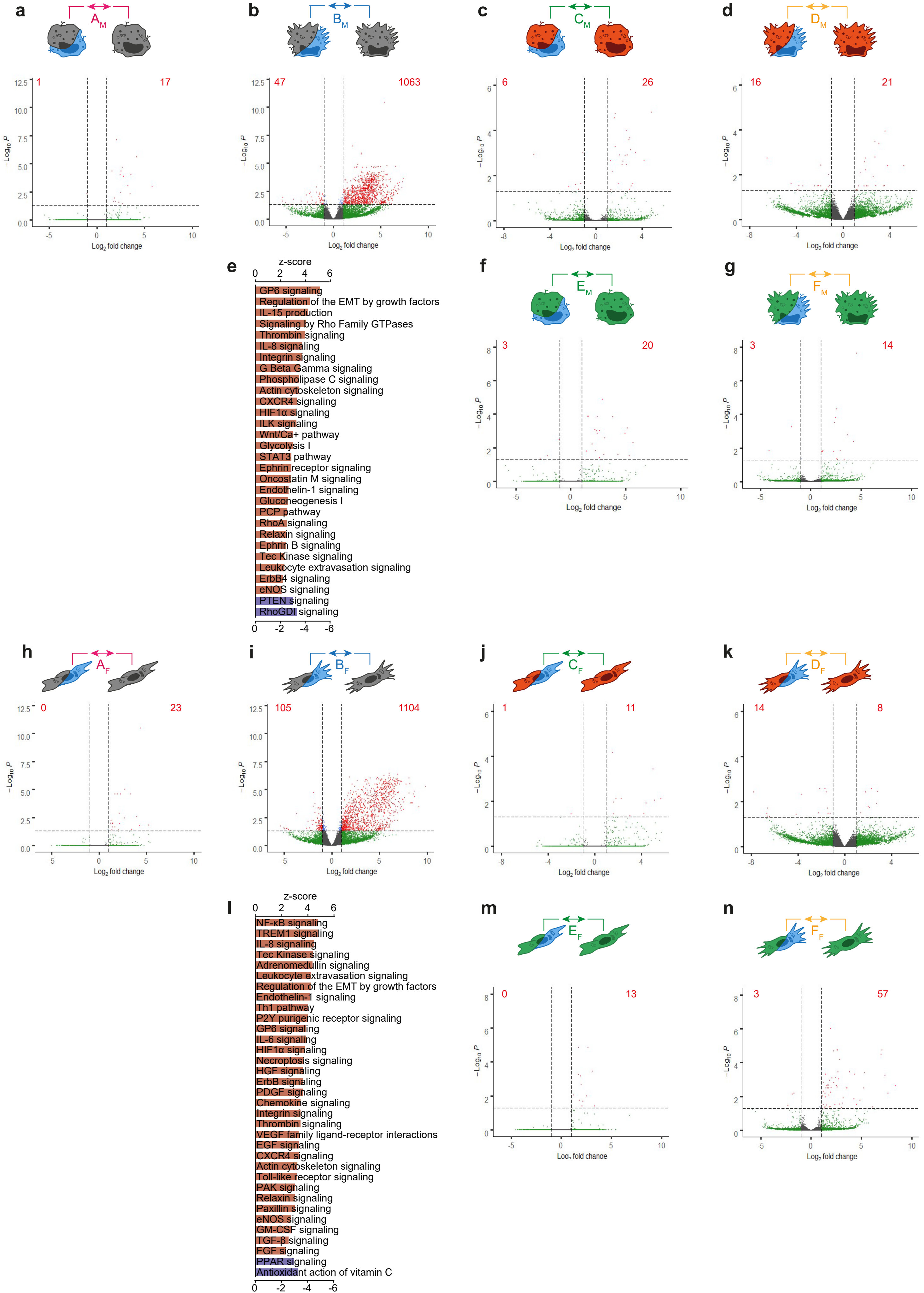
a
A_M
1
17
−Log10 P
Log2 fold change
b
B_M
47
1063
c
C_M
6
26
d
D_M
16
21
e
z-score
GP6 signaling
Regulation of the EMT by growth factors
IL-15 production
Signaling by Rho Family GTPases
Thrombin signaling
IL-8 signaling
Integrin signaling
G Beta Gamma signaling
Phospholipase C signaling
Actin cytoskeleton signaling
CXCR4 signaling
HIF1α signaling
ILK signaling
Wnt/Ca+ pathway
Glycolysis I
STAT3 pathway
Ephrin receptor signaling
Oncostatin M signaling
Endothelin-1 signaling
Gluconeogenesis I
PCP pathway
RhoA signaling
Relaxin signaling
Ephrin B signaling
Tec Kinase signaling
Leukocyte extravasation signaling
ErbB4 signaling
eNOS signaling
PTEN signaling
RhoGDI signaling
f
E_M
3
20
g
F_M
3
14
h
A_F
0
23
i
B_F
105
1104
j
C_F
1
11
k
D_F
14
8
l
z-score
NF-κB signaling
TREM1 signaling
IL-8 signaling
Tec Kinase signaling
Adrenomedullin signaling
Leukocyte extravasation signaling
Regulation of the EMT by growth factors
Endothelin-1 signaling
Th1 pathway
P2Y purigenic receptor signaling
GP6 signaling
IL-6 signaling
HIF1α signaling
Necroptosis signaling
HGF signaling
ErbB signaling
PDGF signaling
Chemokine signaling
Integrin signaling
Thrombin signaling
VEGF family ligand-receptor interactions
EGF signaling
CXCR4 signaling
Actin cytoskeleton signaling
Toll-like receptor signaling
PAK signaling
Relaxin signaling
Paxillin signaling
eNOS signaling
GM-CSF signaling
TGF-β signaling
FGF signaling
PPAR signaling
Antioxidant action of vitamin C
m
E_F
0
13
n
F_F
3
57

Figure S5. Effect of hypoxia on Mφ and Fb exposed to different immune and culture conditions.

a-d, f,g, h-k, m,n) Volcano plots of DEGs induced by hypoxia in Mφ (a-d, f, g) and Fb (h-k, m, n) when compared to normoxic counterparts in single culture without immune challenge (a, h), in cocultures without immune challenge (b, i), in single culture stimulated with LPS+IFNγ (c, j), or in cocultures with LPS+IFNγ (d, k), in single culture stimulated with IL-4 (f, m), or in cocultures with IL-4 (g, n).

e, l) Ingenuity pathway analysis identified functional pathways with a significant positive (z-score ≥ 2, in orange) or negative (z-score ≤ -2, in violet) enrichment associated to DEGs in Mφ (e) and Fb (l), under same experimental conditions reported for the corresponding volcano plot. Enriched pathways were used to generate heatmaps reported in Figure 4e and 4f for Mφ and Fb, respectively.

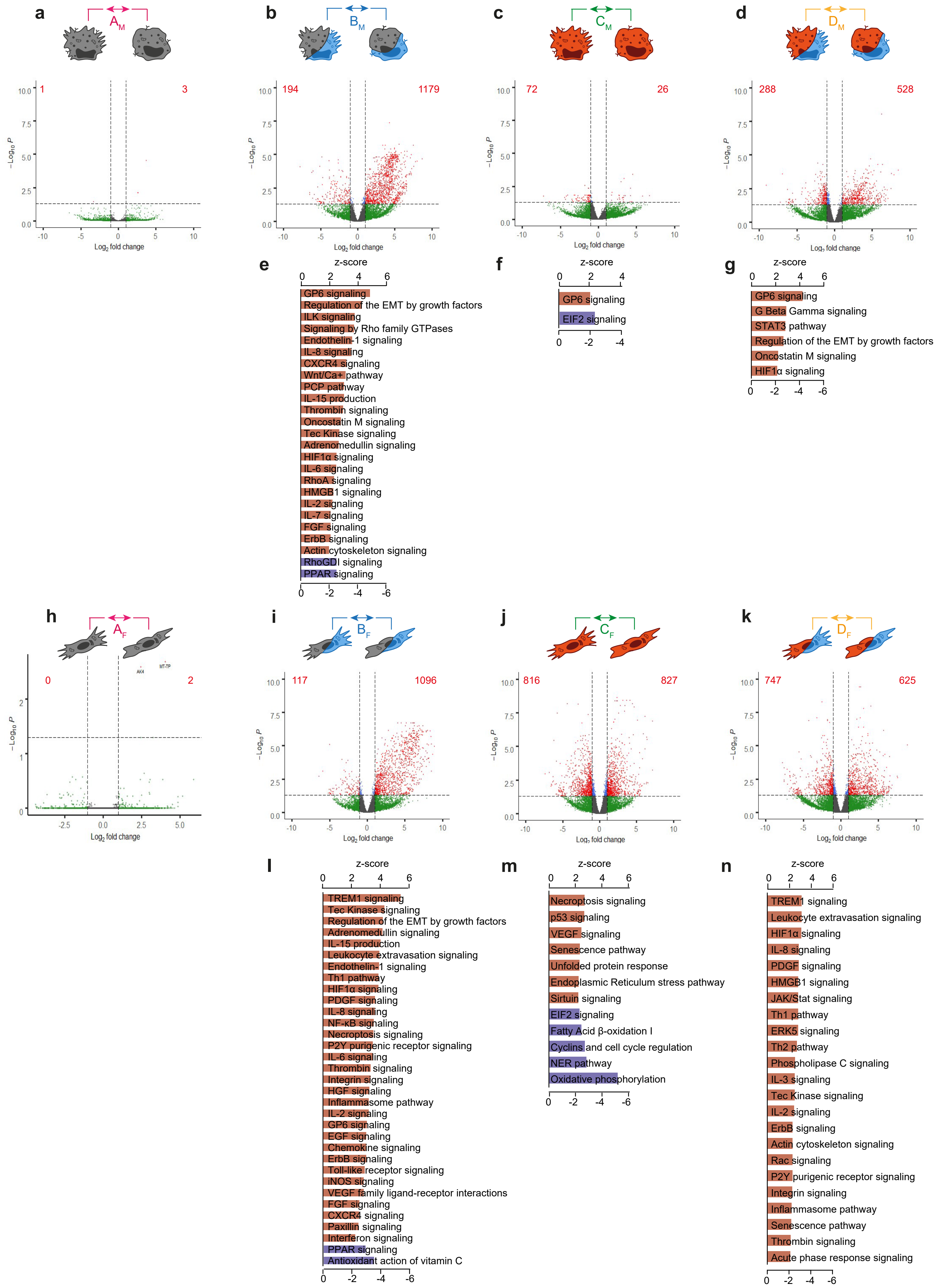

a
A_M
1
3
$-Log_{10} P$
Log_2 fold change
b
B_M
194
1179
$-Log_{10} P$
Log_2 fold change
c
C_M
72
26
$-Log_{10} P$
Log_2 fold change
d
D_M
288
528
$-Log_{10} P$
Log_2 fold change
e
z-score
GP6 signaling
Regulation of the EMT by growth factors
ILK signaling
Signaling by Rho family GTPases
Endothelin-1 signaling
IL-8 signaling
CXCR4 signaling
Wnt/Ca+ pathway
PCP pathway
IL-15 production
Thrombin signaling
Oncostatin M signaling
Tec Kinase signaling
Adrenomedullin signaling
HIF1α signaling
IL-6 signaling
RhoA signaling
HMGB1 signaling
IL-2 signaling
IL-7 signaling
FGF signaling
ErbB signaling
Actin cytoskeleton signaling
RhoGDI signaling
PPAR signaling
f
z-score
GP6 signaling
EIF2 signaling
g
z-score
GP6 signaling
G Beta Gamma signaling
STAT3 pathway
Regulation of the EMT by growth factors
Oncostatin M signaling
HIF1α signaling
h
A_F
0
2
AK4
MT-TP
$-Log_{10} P$
Log_2 fold change
i
B_F
117
1096
$-Log_{10} P$
Log_2 fold change
j
C_F
816
827
$-Log_{10} P$
Log_2 fold change
k
D_F
747
625
$-Log_{10} P$
Log_2 fold change
l
z-score
TREM1 signaling
Tec Kinase signaling
Regulation of the EMT by growth factors
Adrenomedullin signaling
IL-15 production
Leukocyte extravasation signaling
Endothelin-1 signaling
Th1 pathway
HIF1α signaling
PDGF signaling
IL-8 signaling
NF-κB signaling
Necroptosis signaling
P2Y purigenic receptor signaling
IL-6 signaling
Thrombin signaling
Integrin signaling
HGF signaling
Inflammasome pathway
IL-2 signaling
GP6 signaling
EGF signaling
Chemokine signaling
ErbB signaling
Toll-like receptor signaling
iNOS signaling
VEGF family ligand-receptor interactions
FGF signaling
CXCR4 signaling
Paxillin signaling
Interferon signaling
PPAR signaling
Antioxidant action of vitamin C
m
z-score
Necroptosis signaling
p53 signaling
VEGF signaling
Senescence pathway
Unfolded protein response
Endoplasmic Reticulum stress pathway
Sirtuin signaling
EIF2 signaling
Fatty Acid β-oxidation I
Cyclins and cell cycle regulation
NER pathway
Oxidative phosphorylation
n
z-score
TREM1 signaling
Leukocyte extravasation signaling
HIF1α signaling
IL-8 signaling
PDGF signaling
HMGB1 signaling
JAK/Stat signaling
Th1 pathway
ERK5 signaling
Th2 pathway
Phospholipase C signaling
IL-3 signaling
Tec Kinase signaling
IL-2 signaling
ErbB signaling
Actin cytoskeleton signaling
Rac signaling
P2Y purigenic receptor signaling
Integrin signaling
Inflammasome pathway
Senescence pathway
Thrombin signaling
Acute phase response signaling

Figure S6. Effect of cellular interplay on Mφ and Fb exposed to different metabolic and immune conditions.

a-d, h-k) Volcano plots of DEGs induced by coculture in Mφ (a-d) and Fb (h-k) when compared to single cultivated counterparts in normoxia without immune challenge (a, h), in hypoxia without immune challenge (b, i), in normoxia with LPS+IFNγ stimulation (c, j), or in hypoxia with LPS+IFNγ (d, k).

e-g, l-m) Ingenuity pathway analysis identified functional pathways with a significant positive (z-score ≥ 2, in orange) or negative (z-score ≤ -2, in violet) enrichment associated to DEGs in Mφ (e-g) and Fb (l-m), under same experimental conditions reported for the corresponding volcano plot. Enriched pathways were used to generate heatmaps reported in Fig. 5e and 5f for Mφ and Fb, respectively.

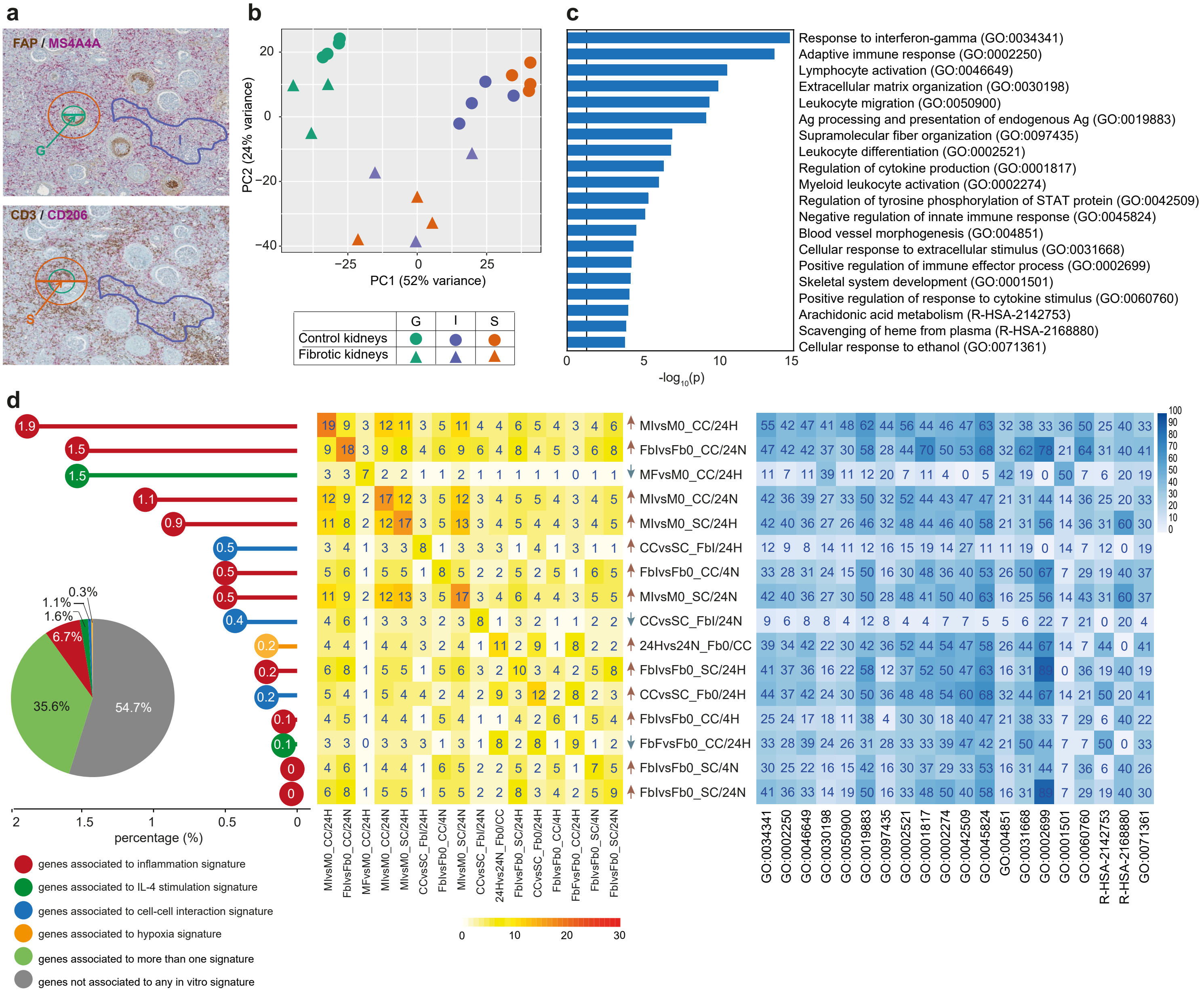

a
FAP / MS4A4A
G
I
CD3 / CD206
S
b
PC2 (24% variance)
PC1 (52% variance)
G
I
S
Control kidneys
Fibrotic kidneys
c
Response to interferon-gamma (GO:0034341)
Adaptive immune response (GO:0002250)
Lymphocyte activation (GO:0046649)
Extracellular matrix organization (GO:0030198)
Leukocyte migration (GO:0050900)
Ag processing and presentation of endogenous Ag (GO:0019883)
Supramolecular fiber organization (GO:0097435)
Leukocyte differentiation (GO:0002521)
Regulation of cytokine production (GO:0001817)
Myeloid leukocyte activation (GO:0002274)
Regulation of tyrosine phosphorylation of STAT protein (GO:0042509)
Negative regulation of innate immune response (GO:0045824)
Blood vessel morphogenesis (GO:004851)
Cellular response to extracellular stimulus (GO:0031668)
Positive regulation of immune effector process (GO:0002699)
Skeletal system development (GO:0001501)
Positive regulation of response to cytokine stimulus (GO:0060760)
Arachidonic acid metabolism (R-HSA-2142753)
Scavenging of heme from plasma (R-HSA-2168880)
Cellular response to ethanol (GO:0071361)
-log10(p)
d
MIvsM0_CC/24H
FbIvsFb0_CC/24N
MFvsM0_CC/24H
MIvsM0_CC/24N
MIvsM0_SC/24H
CCvsSC_FbI/24H
FbIvsFb0_CC/4N
MIvsM0_SC/24N
CCvsSC_FbI/24N
24Hvs24N_Fb0/CC
FbIvsFb0_SC/24H
CCvsSC_Fb0/24H
FbIvsFb0_CC/4H
FbFvsFb0_CC/24H
FbIvsFb0_SC/4N
FbIvsFb0_SC/24N
percentage (%)
54.7%
35.6%
6.7%
1.6%
1.1%
0.3%
genes associated to inflammation signature
genes associated to IL-4 stimulation signature
genes associated to cell-cell interaction signature
genes associated to hypoxia signature
genes associated to more than one signature
genes not associated to any in vitro signature

Figure S7. Gene overlap analysis of ex vivo nephrectomies with in vitro signatures.

a) IHC of renal section: green circle = glomeruli (G); orange circle = surrounding area (S); violet area = interstitium (I). Myofibroblasts = FAP^+ Fb (upper panel), T cells = $CD3^+$ cells (lower panel), alternatively activated Mφ = $MS4A4A^+$ (upper panel) or $CD206^+$ cells (lower panel).

b) PCA of total ex vivo samples. Colors indicate different anatomical regions (green = glomeruli, violet = interstitium, orange = surrounding); triangles = nephrectomies, dots = controls.

c) Bar graph reports pathways significantly enriched ($\log_{10}$(p-value) $\geq$ 1.3) in pathological vs control samples as calculated by Metascape software (for statistical type of analysis see ref 46).

d) Overlap analysis was performed comparing DEGs of ex vivo pathological vs control comparison with DEGs of each in vitro comparisons, which defined 78 corresponding signatures. Pie chart reveals that: 35.6% of the ex vivo signature is explained by at least one in vitro signature (in light green); 9.7% is specifically explained by only one in vitro signature and different colors are referred to the discriminating variable of in vitro signatures: inflammation in red, IL-4 stimulation in green, cell-cell interaction in blue, hypoxia in yellow; the remaining 54.7% (2,554 genes) is not explained by in vitro model (in grey). Dot chart (on left) reports in vitro signatures enriched in ex vivo model on vertical axis and their relative contribution with univocal expressed genes: white numbers in each dot represent relative percentages on the totality of explained and not explained genes. The central heatmap highlights the overlapping percentage of the same 16 in vitro signatures on the ex vivo signature. On the diagonal, numbers indicate the overlapping percentage of each signature; the other numbers explain the overlapping percentage shared between two in vitro and the ex vivo signatures. Color intensity indicates the overlapping percentage level referred to ex vivo signature. Arrows near each in vitro signature indicate the positive (red) or negative (blue) enrichment of first term of in vitro comparison. Best representative enriched in vitro signatures were also validated by GSEA analysis as reported in Fig. S8. The heatmap on the right reports Metascape pathways in ex vivo comparison (panel b) with the percentage of enrichment given by each in vitro signature (on the left).

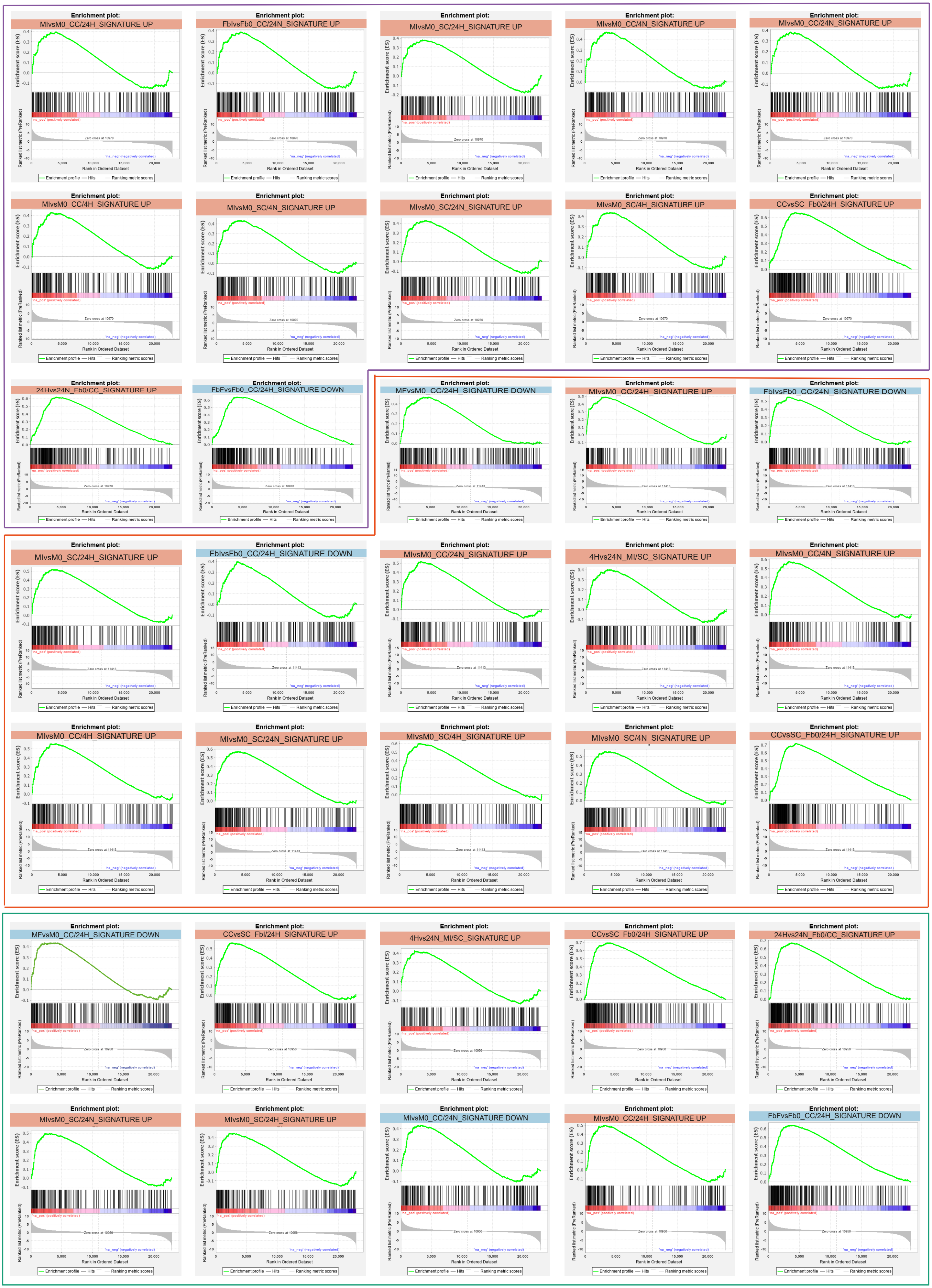
Enrichment plot:
MIvsM0_CC/24H_SIGNATURE UP
Enrichment plot:
FbIvsFb0_CC/24H_SIGNATURE UP
Enrichment plot:
MIvsM0_SC/24H_SIGNATURE UP
Enrichment plot:
MIvsM0_CC/4N_SIGNATURE UP
Enrichment plot:
MIvsM0_CC/24N_SIGNATURE UP
Enrichment plot:
MIvsM0_CC/4H_SIGNATURE UP
Enrichment plot:
MIvsM0_SC/4N_SIGNATURE UP
Enrichment plot:
MIvsM0_SC/24N_SIGNATURE UP
Enrichment plot:
MIvsM0_SC/4H_SIGNATURE UP
Enrichment plot:
CCvsSC_Fb0/24H_SIGNATURE UP
Enrichment plot:
24Hvs24N_Fb0/CC_SIGNATURE UP
Enrichment plot:
FbFvsFb0_CC/24H_SIGNATURE DOWN
Enrichment plot:
MFvsM0_CC/24H_SIGNATURE DOWN
Enrichment plot:
MIvsM0_CC/24H_SIGNATURE UP
Enrichment plot:
FbIvsFb0_CC/24H_SIGNATURE DOWN
Enrichment plot:
MIvsM0_SC/24H_SIGNATURE UP
Enrichment plot:
FbIvsFb0_CC/24H_SIGNATURE DOWN
Enrichment plot:
MIvsM0_CC/24N_SIGNATURE UP
Enrichment plot:
4Hvs24N_MI/SC_SIGNATURE UP
Enrichment plot:
MIvsM0_CC/4N_SIGNATURE UP
Enrichment plot:
MIvsM0_CC/4H_SIGNATURE UP
Enrichment plot:
MIvsM0_SC/24N_SIGNATURE UP
Enrichment plot:
MIvsM0_SC/4H_SIGNATURE UP
Enrichment plot:
MIvsM0_SC/4N_SIGNATURE UP
Enrichment plot:
CCvsSC_Fb0/24H_SIGNATURE UP
Enrichment plot:
MFvsM0_CC/24H_SIGNATURE DOWN
Enrichment plot:
CCvsSC_FbI/24H_SIGNATURE UP
Enrichment plot:
4Hvs24N_MI/SC_SIGNATURE UP
Enrichment plot:
CCvsSC_Fb0/24H_SIGNATURE UP
Enrichment plot:
24Hvs24N_Fb0/CC_SIGNATURE UP
Enrichment plot:
MIvsM0_SC/24N_SIGNATURE UP
Enrichment plot:
MIvsM0_SC/24H_SIGNATURE UP
Enrichment plot:
MIvsM0_CC/24N_SIGNATURE DOWN
Enrichment plot:
MIvsM0_CC/24H_SIGNATURE UP
Enrichment plot:
FbFvsFb0_CC/24H_SIGNATURE DOWN

Figure S8. Single sample Gene Set Enrichment Analysis of ex vivo nephrectomies signature with significant overlapping in vitro signatures.

GSEA of in vitro signatures with an overlapping percentage on the ex vivo respective signature ≥ 15%, as also reported in Fig. S7. Signatures underlined in red are positive enriched for the first term compared to the second term (_signature UP); on the contrary, signatures in blue are negative enriched for the first term (_signature DOWN). Violet, orange and green boxes refer to in vitro signatures enriched in ex vivo signature of interstitium, glomeruli surrounding area and glomeruli, respectively.

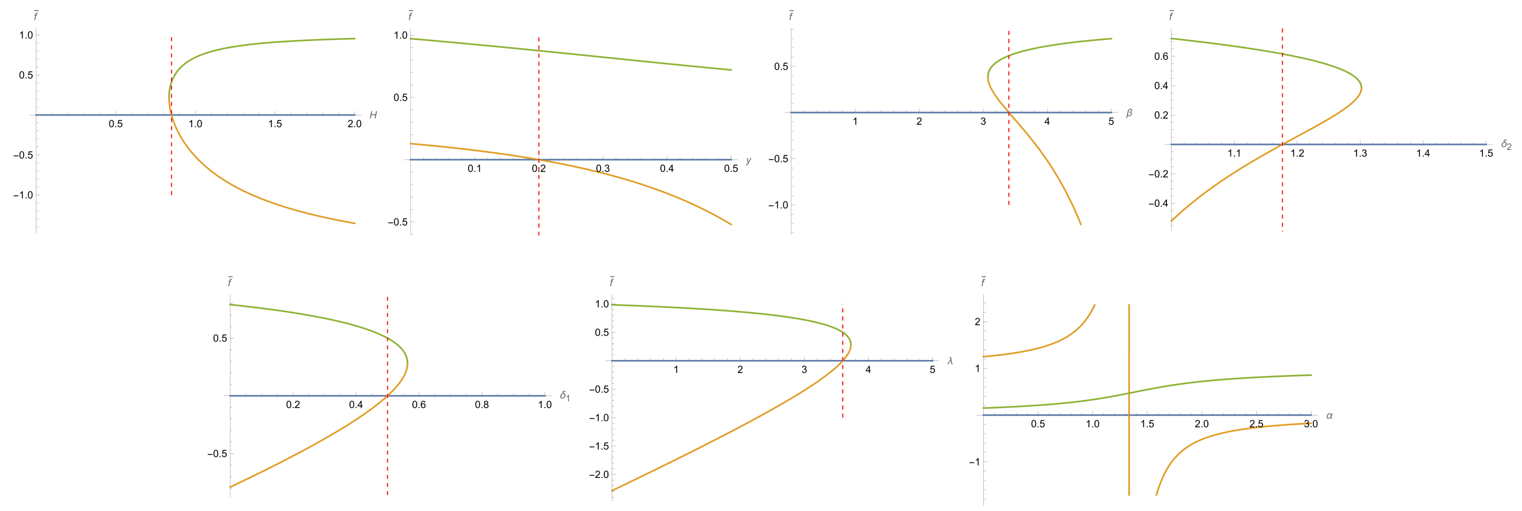

Figure S9. Bifurcation diagrams.

Plots show different steady states of the model represented with lines of different colors as functions of a varying parameter value. A red dashed line indicates the stability condition of the healthy steady state reported in the main text. Parameter values were set at α=2, β=4, δ1=0.2, δ2=1, H=1, λ=3, and y=0.5 when not variables. Left to right: Top row: variable H, y, β, and δ2. Bottom row: variable δ1, λ, and α.

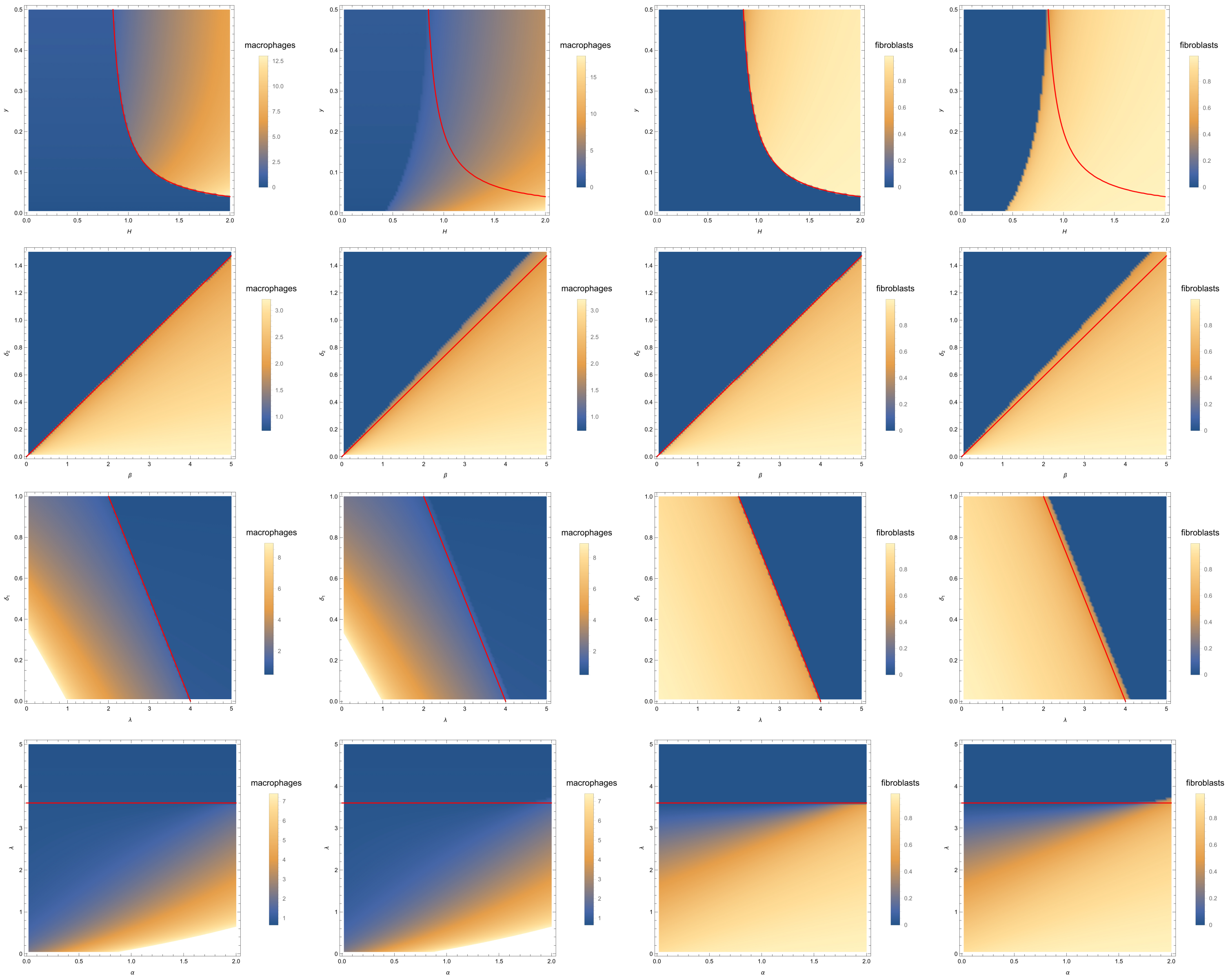

Figure S10. Numerical simulations of Mφ and Fb values at t=1000.

Simulation values should be close to the steady state. A red line shows the stability condition of the healthy state reported in the main text. Parameter values were set at α=2, β=4, δ_1=0.2, δ_2=1, H=1, λ=3, and y=0.5 when not variables. Top to bottom rows: variable y vs. H, δ2 vs. β, δ1 vs. λ, and λ vs. α. First and second column: Mφ concentrations. Third and fourth column: Fb concentration. First and third column: initial conditions f(0)=m(0)=1×10^{-3}. Second and fourth column: initial conditions m(0)=10, f(0)=0.2.

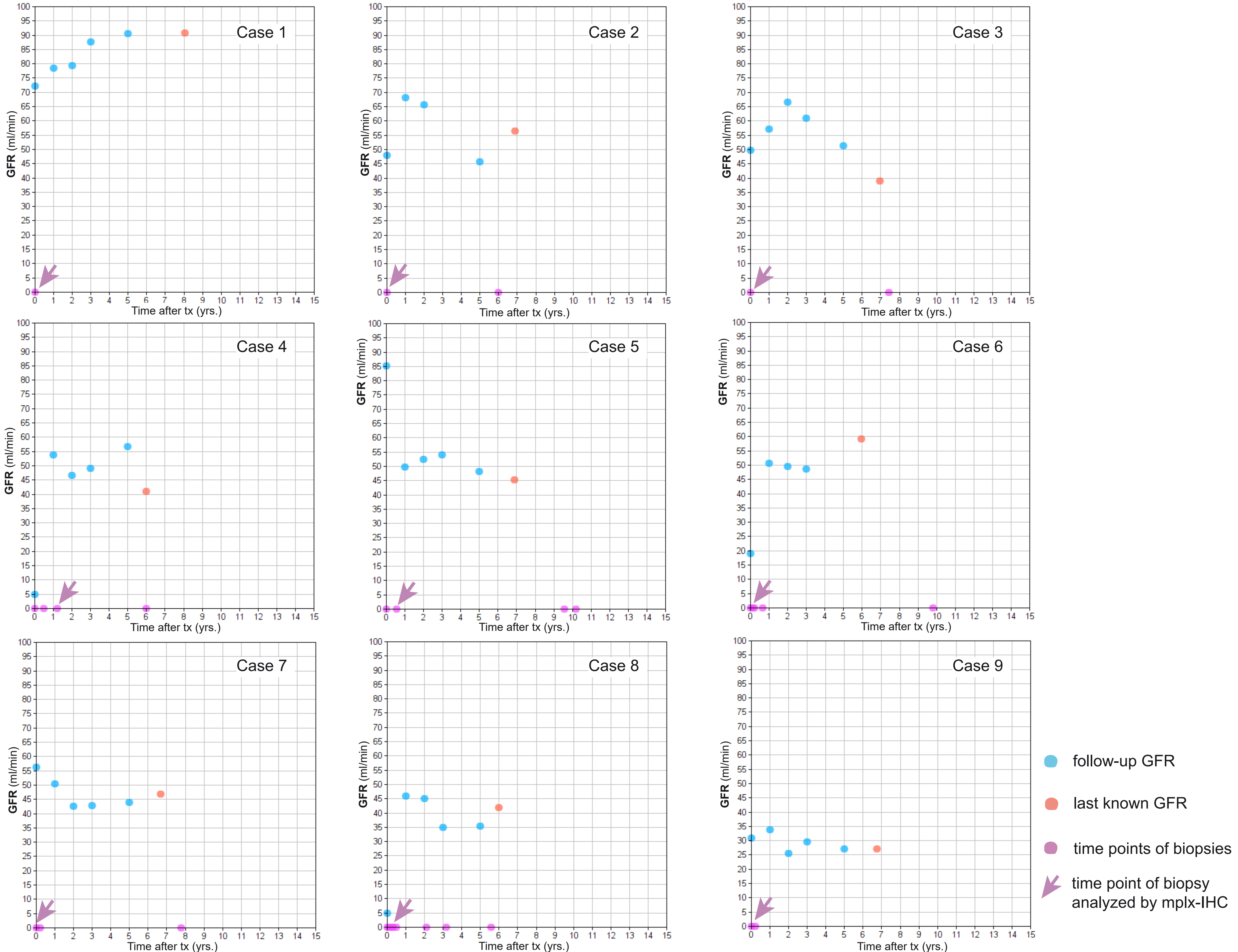
Case 1
Case 2
Case 3
Case 4
Case 5
Case 6
Case 7
Case 8
Case 9
GFR (ml/min)
Time after tx (yrs.)
follow-up GFR
last known GFR
time points of biopsies
time point of biopsy analyzed by mplx-IHC

Figure S11. Renal function over time and time points of longitudinal renal biopsy evaluation. The graphs depict the glomerular filtration rate (GFR) and the time points when consecutive biopsies were performed for the 9 patients depicted in Fig. 10c, selected to represent a large range of different underlying pathologies and patient characteristics (see Table S5). These cases are part of a large cohort of patients subjected to B cell analysis in an earlier work [see ref. 29 and 30] and were diagnosed as “borderline” at some point in the clinical course (see arrows highlighting that point in time).Though full criteria for rejection was not met, according to the Banff classification (version valid at time of diagnosis) this indicates the presence of considerable inflammation. The pie charts in the corresponding Fig. 10c show the cellular infiltrates at these time points in detail.

Supplementary tables

Case	Age Group (years)	Time Post-Tx	Clinical condition	Pathological assessment*	Banff Class.*	Renal function ml/min
A	61-65	7 mts. (N)	Loss of graft function after ca. 1 year	ABMR / TCMR	Cat 2, Type III<br>Cat 4, Type III<br>Cat 5, Grade II	loss
B	76-80	5 yrs. (N)	Loss of graft function after ca. 3 years	ABMR	Cat 2, Type III<br>Cat 5, Grade III	loss
C	61-65	NA (N)	kidney tumor in graft	Clear cell renal cell carcinoma, ABMR/ TCMR	Cat 5, Grade III	loss
D	51-55	3 mts. (N)	Loss of graft function after ca.1 year	TCMR	Cat 4, Type IIA<br>Cat 5, Grade I	loss
1	56-60	3 mts (C)	glomerulonephritis (GN)	Focal predominantly interstitial immune cell infiltrates	Borderline	GFR >90 (8 yrs.)
2	46-50	3 mts. (C)	Adult renal cystic disease (ARCD)	Minimal focal interstitial immune cell infiltrates	Borderline	GFR >55 (6 yrs.)
3	61-65	3 mts (C)	GN	Low focal interstitial infiltrates, no tubulitis	Borderline	GFR ~40 (7 yrs.)
4	41-45	15 mts. (I)	GN	Interstitial infiltrates and minimal tubulitis	Borderline	GFR >40 (6 yrs.)
5	36-40	10 mts. (I)	Fabry's disease	Focal tubulointerstitial damage &glomerulosclerosis	Borderline	GFR >45 (>6 yrs.)
6	51-55	2 mts (C)	ARCD	Few interstitial mononuclear infiltrates	Borderline	GFR >55 (>6 yrs.)
7	65-70	3 mts. (C)	polycystic kidney disease	Moderate focal interstitial immune cell infiltrates	Borderline	GFR >45 (>6 yrs.)
8	51-55	3 mts (C)	focal segmental glomerulosclerosis	Mononuclear interstitial infiltrates	Borderline	GFR >40 (6 yrs.)
9	51-55	2 mts (C)	ARCD	Moderate focal tubular atrophy, interstitial infiltrates	Borderline	GFR ~30 (>6 yrs.)
10	61-70	9 mts. (I)	unknown	Diagnostic criteria ABMR or TCMR not met	Borderline	NA
11	61-65	5 yrs. (I)	unknown	Suspicion of TCMR (borderline)	Borderline	Good (>10 yrs.)

Supplementary Table 1.

Clinical characteristics of patients A-D (nephrectomies) and patients 1-11 (renal biopsies), referring to Figures 6 and 10, respectively. Patients were grouped into age groups in 5 years increments. The

male/female distribution was largely balanced (8 female, 7 male patients). GFR measurements (when available) were grouped in 5 ml/min increments.

N = nephrectomy; C = control biopsy; I = indicated biopsy; GFR = glomerular filtration rate; Cat = category (according to the Banff – classification); ABMR = antibody – mediated (“humoral”) rejection; TCMR = T-cell – mediated (“cellular”) rejection; mts. = months; yrs. = years; NA = not available

Supplementary methods

Multilevel analysis of transcriptomic data generated in the in vitro model.

The relative contribution of each variable and multiple combined effects was evaluated by supervised analysis at three levels of increasing complexity. In the 1st level analysis, one by one variable was taken into account: differential gene expression analysis was assessed on normalized counts and performed in paired. For each single comparison, we defined an ad hoc design matrix taking into account replicates, phenotype, culture condition, O_2 status, and polarization information. For each gene statistical significance of differential expression was tested using the QL F-test, only genes with a False Discovery Rate (FDR, p-value adjusted considering Benjamini-Hochberg correction) ≤ 0.05 were selected and referred to as "differentially expressed genes" (DEG). DEGs positively and negatively regulated were then shown in volcano plots. DEGs associated to each comparison and their logFC values ($|logFC| \geq 1$) were used for the identification of enriched pathways using the Ingenuity Pathway Analysis software (IPA v01-13; Qiagen). Only pathways with $|z\text{-score}| \geq 2$ and $log_{10}(p\text{-value}) \geq 1.3$ were selected. 1st level comparisons were based on each of the following variables:

- OX = hypoxia (H) vs normoxia (N) (23 analyses)
- COL = coculture (CC) vs single culture (SC) (23 analyses)
- POL = IFNγ or IL-4 vs no stimulation (0) (32 analyses)

The 2nd level analysis started from the signatures defined in the first level and analyzed their overlaps (Euler-Venn diagrams) to evaluate the following combination of two variables:

- (OX)|(COL) = [(H vs N)|CC vs (H vs N)|SC] (12 analyses)
- (COL)|(OX) = [(CC vs SC)|H vs (CC vs SC)|N] (12 analyses)
- (POL)|(COL) = [(POL vs 0)|CC vs (POL vs 0)|SC] (16 analyses)
- (POL)|(OX) = [(POL vs 0)|H vs (POL vs 0)|N] (16 analyses)

The 3rd level analysis started from the signatures defined in the first and second levels and analyzed their overlaps (Euler-Venn diagrams) to evaluate the following combination of three variables:

- [(POL)|OX]COL = [(POL vs 0)|H vs (POL vs 0)|N]|CC vs [(POL vs 0)|H vs (POL vs 0)|N]|SC (8 analyses)
- [(POL)|COL]OX = [(POL vs 0)|CC vs (POL vs 0)|SC]|H vs [(POL vs 0)|CC vs (POL vs 0)|SC]|N (8 analyses)
- [(COL)|POL]OX = [(CC vs SC)|POL vs (CC vs SC)|0]|H vs [(CC vs SC)|POL vs (CC vs SC)|0]N (8 analyses)
- [(COL)|OX]POL = [(CC vs SC)|H vs (CC vs SC)|N]|POL vs [(CC vs SC)|H vs (CC vs SC)|N]|0 (8 analyses)
- [(OX)|POL]COL = [(H vs N)|POL vs (H vs N)|0]|CC vs [(H vs N)|POL vs (H vs N)|0]|SC (8 analyses)
- [(OX)|COL]POL = [(H vs N)|CC vs (H vs N)|SC]|POL vs [(H vs N)|CC vs (H vs N)|SC]|0 (8 analyses)

Pathway enrichment analysis was performed also by Metascape online tool, considering DEGs with the same parameters applied for IPA. Only pathways with a $-\log_{10}$(p-value) that exceeded the 1.3 threshold value were selected and reported in Supplementary Data 1-4.

Pre-ranked Gene Set Enrichment Analysis (GSEA).

GSEA was performed in order to evaluate which in vitro signatures were significantly enriched in ex vivo data. In the first step we defined in vitro signatures: for each previous performed differential expression analysis of in vitro data, two signatures were created: an "UP regulation" signature (gene subset parameters: FDR $\leq$ 0.05 and logFC $\geq$ 1) and a "DOWN regulation" signature (FDR $\leq$ 0.05 and logFC $\leq$ -1). Only signatures with more than 15 genes were taken into account. If the length of the signature was greater than 250 genes, only first 250 (ranked by logFC) genes were considered.

Then we generated four ranked lists (defined on logFC values) referred to the general comparison of pathological and control samples and to the same comparison but specific for the three distinct anatomical regions (interstitium, glomeruli surrounding area and glomeruli). Finally, pre-ranked GSEA was performed for each ranked list applying 1000 permutations, weighted enrichment statistic method; seed set for permutation was 149.

Gene overlap analysis.

We defined in vitro and ex vivo signatures on previous performed differential expression analysis (78 and 4, respectively) considering FDR$\leq$ 0.05 and $|logFC| \geq 1$ and p-value ≤ 0.01 and $|logFC| \geq 1$. The overlap analysis was performed with Gene Overlap Bioconductor package, which evaluates p-value, odds-ratio and Jaccard index of in vitro and ex vivo signatures overlaps.

Percentage explained analysis.

Enriched in vitro signatures and overlap significance for each ex vivo region are known. Considering one by one region, only in vitro signatures respecting specific filters were taken into account (for GSEA: Normalized Enrichment score greater than 1, nominal p-value lower than 0.01; for Gene Overlap: p-value < 0.05 and odds-ratio > 1).

Let signature A, B, C etc. be in vitro signatures (with AA as last signature), let signature α be the ex vivo signature of the selected region, let signature $_M$ be the Metascape signature, K the number of the enriched Metascape signatures and let n assume the meaning of "number of", considering signatures as defined in "Gene overlap" section, we determined:

a) percentage of ex vivo genes explained by in vitro model (with genes defined as union of all genes of the selected in vitro signatures). The results are represented in the pie chart.

$$\frac{n\ (\bigcup_{i=A}^{AA} signature_i \cap signature_\alpha)}{numeric(signature_\alpha)} \cdot 100\ \%$$

b) percentage of ex vivo genes explained by only one signature of in vitro model (unique genes) represented in the bar plot.

$$\frac{n\left(\left(signature_i - \cup_{j=A,j\neq i}^{AA} signature_j\right)\right) \cap signature_\alpha)}{n(signature_\alpha)} \cdot 100\ \%$$

$$i \in [A, AA]$$

c) percentage of ex vivo genes explained by one signature of in vitro model (diagonal values, heatmaps in the middle panel of Figures 7c, 8c, 9c and S7d):

$$\frac{n(signature_i \cap signature_\alpha)}{n(signature_\alpha)} \cdot 100\ \% i \in [A, AA]$$

d) percentage of ex vivo genes explained by two signatures of in vitro model (values outside the diagonal, heatmaps in the middle panel of Figures 7c, 8c, 9c and S7d):

$$\frac{n(signature_i \cap signature_j \cap signature_\alpha)}{n(signature_\alpha)} \cdot 100\ \%$$

$$i \in [A, AA], j \in [A, AA], i \neq j$$

e) percentage of ex vivo genes in enrichment analysis (heatmaps in the right panel of Figures 7c, 8c, 9c and S7d):

$$\frac{n(signature_i \cap signature_{M_k})}{n(signature_{M_k})} \cdot 100\ \%$$

$$i \in [A, AA], k \in [1, K]$$

Specific pathway analysis.

Considering glomeruli, interstitium, and glomeruli surrounding area, genes not shared were marked as region-specific genes. Signatures obtained as just described were been input data in new pathway analyses. Pathway resulted were subsets of previous pathway analyses (see the Methods section) and were highlighted with asterisks.

Real-time quantitative PCR (qPCR).

Total mRNA was extracted from human macrophages and fibroblasts using DirectZOL RNA miniprep kit (Cat.No:R2050; Zymo Research) according to the manufacturer's instructions. Reverse transcription was done using High-Capacity cDNA Reverse Transcription kit (Cat.No:4368813; Applied Biosystems). qPCR was performed using TaqMan Fast Advanced Master Mix 2X (Applied Biosystems) and TaqMan probes (Cat.No:4444557; Thermo Fisher) for *GLUT1* (Hs00892681_m1), *VEGFA* (Hs00900055_m1), and *CXCR4* (Hs00607978_s1), *BNIP3* (Hs00969291_m1). Reactions were performed on a VIIA-7 qPCR Detection System (Software V1.2 Applied Biosystems). The thermal cycling conditions were standard fast-cycling. Relative expression values were calculated using the ΔΔCT method normalized on the housekeeping gene *GAPDH* (Hs_99999905_m1). Source data are reported in Source Data file.

Statistical analysis.

Statistical analysis was performed using Prism version 7.0 (GraphPad software). Comparisons were calculated by two-way ANOVA test applying Sidak's multiple comparisons correction. The level of statistically significant difference was defined as $p \leq 0.05$.

Mathematical modelling appendix: differences to the Adler et al model.

The differential equation model by Adler et al. (see Eqs. 1-4 of the Supplemental Information in Adler et al. 2020) describes the macrophage-fibroblast (S1,S2) interplay based on the CSF and PDGF dynamics (S3,S4):

$$\dot{m} = \frac{\lambda_2 \mathrm{CSF}}{\mathrm{CSF} + k_2} m - \mu_1 m, \qquad \text{(S1)}$$

$$\dot{f} = \frac{\lambda_1 \mathrm{PDGF}}{\mathrm{PDGF} + k_1} f(1 - f) - \mu_2 f, \text{(S2)}$$

$$\dot{\mathrm{CSF}} = \beta_1 f - \alpha_1 \frac{\mathrm{CSF}}{\mathrm{CSF} + k_2} m - \gamma\, \mathrm{CSF}, \qquad \text{(S3)}$$

$$\dot{\mathrm{PDGF}} = \beta_2 m + \beta_3 f - \alpha_2 f \frac{\mathrm{PDGF}}{\mathrm{PDGF} + k_1} - \gamma \mathrm{PDGF}, \qquad \text{(S4)}$$

Setting the protein dynamics in steady state (Eqs.S3, S4) and substituting into Eqs. (S1) and (S2),our main text model (Box 1) maintains the structure of the following equations (S5, S6) as shown below (parameters c and c' are proportional to CSF and PDGF steady states, respectively):

$$\dot{m} = (\beta_1' f + c)m - \mu_1 m, \qquad \text{(S5)}$$

$$\dot{f} = (\beta_2' m + \beta_3' f + c')(1 - f) - \mu_2 f \text{ (S6)}$$

However, our model includes the effect of hypoxia and inflammation, which is derived from the analysis of our experimental observation. Therefore the proliferation and death parameters of our main text model (Box 1) are non-trivially depending also on our experimental parameters y (inflammation) and H (hypoxia). To facilitate the reader, we repeat our model (Box 1) below, where is easy to identify the differences with the system (S5, S6):

$$\dot{m} = y - m \frac{\lambda y + \delta_1}{1 + \lambda y} + \alpha H \frac{(y + 1)m}{m + 1 + \lambda y} f, \qquad (1)$$

$$\dot{f} = \beta H \frac{m}{1 + \lambda y} f(1 - f) - \delta_2 f, \quad (2)$$

Robustness analysis of model's dynamic behaviour.

To assess the robustness of the model's qualitative behaviours, we our analysis with respect to variations all free parameter in the model.

Bifurcation analysis.

As a first step, we performed a bifurcation study by fixing all parameter values and varying the values of each parameter individually (Fig. S8). We found that all parameters are bifurcating, except for the parameter α, which only affects the value of the fibrotic steady state. With respect to all other parameters, we found that two bifurcations take place: for a considerable parameter range, only the healthy steady state exists and is globally stable. At certain parameter values, a saddle-node bifurcation occurs which creates a stable fibrotic steady state, and a saddle point. In this parameter range, the system is bistable and the initial conditions dictate whether the system ends up in the fibrotic or healthy steady states. Further change of the parameter values, a second bifurcation happens when the saddle node and healthy steady states collide, a transcritical bifurcation. Thus, the healthy steady state loses stability, and the fibrotic steady state becomes the only biologically relevant stable steady state. No other bifurcations occur. Since the system is two-dimensional, no chaotic behaviour or hidden attractors are possible. Thus, the only possible long-time behaviour according to the model is observing the fibrotic or healthy steady states. Furthermore, we confirmed this long-time dynamic behaviour through numerical calculation of the ODE system solutions (Fig. S9). For all parameters, we find that after a long time, solutions either reach the healthy or the fibrotic steady state, according to the stability criterion of the healthy steady state reported in the main text, when the initial fibroblast and macrophage concentrations are low. When the initial macrophage and fibroblast concentrations are high, we observe the bistable regime (after the saddle-node bifurcation has happened) as a small region in the parameter space where the solutions reach the fibrotic steady state even below the instability threshold of the healthy steady state. The sole noteworthy behaviour we find from simulations is visible in the H vs. y parameter space. While for

all other parameters, the bistability region remains small, in this case the bistable region grows with decreasing inflammation y, which may seem counterintuitive at first. However, we should recall that the fibrotic steady state in the bistable regime can only be reached for high initial macrophage and fibroblast concentrations. If inflammation is low (or absent) then only pro-fibrotic macrophages and fibroblasts are present, which cannot decrease significantly on their own, thus leading to fibrosis. In other words, high levels of active fibroblasts in the absence of damage can only lead to fibrosis.
