# Supplementary material for: Understanding fibrosis pathogenesis via modeling macrophage-fibroblast interplay in immune-metabolic context
Source: Nat Commun. 2022 Oct 30;13:6499. doi: 10.1038/s41467-022-34241-5 (PMC9618579; doi:10.1038/s41467-022-34241-5)
Supplement: Supplementary file 2 — Description of Additional Supplementary Files [file 41467_2022_34241_MOESM2_ESM.pdf]

Description of Additional Supplementary Files

Supplementary Data 1.

Enriched pathways from Metascape analysis reported for most informative comparisons when cells were stimulated by LPS+IFNγ in different metabolic and culture settings. Each sheet is named with the comparison label followed by '_up' or '_down', indicating up-regulated or down-regulated pathways, respectively. For each pathway is reported the related annotation term, the log(p-value) and the list of enriched genes. Statistics is automatically calculated by Metascape, for details please refer to Zhou et al. Nature Commun. 2019 10(1):1523. DEGs used for this analysis are the same used for IPA in Figure 2 and S2.

Supplementary Data 2.

Enriched pathways from Metascape analysis reported for most informative comparisons when cells were stimulated by IL-4 in different metabolic and culture settings. Each sheet is named with the comparison label followed by '_up' or '_down', indicating up-regulated or down-regulated pathways, respectively. For each pathway is reported the related annotation term, the log(p-value) and the list of enriched genes. Statistics is automatically calculated by Metascape, for details please refer to Zhou et al. Nature Commun. 2019 10(1):1523. DEGs used for this analysis are the same used for IPA in Figure 3 and S3.

Supplementary Data 3.

Enriched pathways from Metascape analysis reported for most informative comparisons when cells were treated with hypoxia in pro-inflammatory or resting context and in different culture

settings. Each sheet is named with the comparison label followed by '_up' or '_down', indicating up-regulated or down-regulated pathways, respectively. For each pathway is reported the related annotation term, the log(p-value) and the list of enriched genes. Statistics is automatically calculated by Metascape, for details please refer to Zhou et al. Nature Commun. 2019 10(1):1523. DEGs used for this analysis are the same used for IPA in Figure 4 and S5.

Supplementary Data 4.

Enriched pathways from Metascape analysis reported for most informative comparisons when cells were co-cultivated in different metabolic condition, in pro-inflammatory or resting context. Each sheet is named with the comparison label followed by '_up' or '_down', indicating up-regulated or down-regulated pathways, respectively. For each pathway is reported the related annotation term, the log(p-value) and the list of enriched genes. Statistics is automatically calculated by Metascape, for details please refer to Zhou et al. Nature Commun. 2019 10(1):1523. DEGs used for this analysis are the same used for IPA in Figure 5 and S6.
