# Supplementary material for: Understanding fibrosis pathogenesis via modeling macrophage-fibroblast interplay in immune-metabolic context
Source: Nat Commun. 2022 Oct 30;13:6499. doi: 10.1038/s41467-022-34241-5 (PMC9618579; doi:10.1038/s41467-022-34241-5)
Supplement: Supplementary file 7 — Reporting Summary [file 41467_2022_34241_MOESM7_ESM.pdf]

nature portfolio

Corresponding author(s): Massimo Locati

Last updated by author(s): Sep 16, 2022

Reporting Summary

Nature Portfolio wishes to improve the reproducibility of the work that we publish. This form provides structure for consistency and transparency in reporting. For further information on Nature Portfolio policies, see our Editorial Policies and the Editorial Policy Checklist.

Statistics

For all statistical analyses, confirm that the following items are present in the figure legend, table legend, main text, or Methods section.

n/a	Confirmed	
☐	☒	The exact sample size (*n*) for each experimental group/condition, given as a discrete number and unit of measurement
☐	☒	A statement on whether measurements were taken from distinct samples or whether the same sample was measured repeatedly
☐	☒	The statistical test(s) used AND whether they are one- or two-sided<br>*Only common tests should be described solely by name; describe more complex techniques in the Methods section.*
☒	☐	A description of all covariates tested
☐	☒	A description of any assumptions or corrections, such as tests of normality and adjustment for multiple comparisons
☐	☒	A full description of the statistical parameters including central tendency (e.g. means) or other basic estimates (e.g. regression coefficient) AND variation (e.g. standard deviation) or associated estimates of uncertainty (e.g. confidence intervals)
☐	☒	For null hypothesis testing, the test statistic (e.g. *F*, *t*, *r*) with confidence intervals, effect sizes, degrees of freedom and *P* value noted<br>*Give P values as exact values whenever suitable.*
☒	☐	For Bayesian analysis, information on the choice of priors and Markov chain Monte Carlo settings
☒	☐	For hierarchical and complex designs, identification of the appropriate level for tests and full reporting of outcomes
☒	☐	Estimates of effect sizes (e.g. Cohen's *d*, Pearson's *r*), indicating how they were calculated

Our web collection on statistics for biologists contains articles on many of the points above.

Software and code

Policy information about availability of computer code

Data collection	FACS sorting: BD FACSDiva 8.0.1<br>qRT-PCR: ViiA7 Software V1.2 (Applied Biosystems)
Data analysis	RNA sequencing analysis:<br>analysis performed with R version 3.5.2 and following packages:<br>AnnotationDbi 1.44.0<br>org.Hs.eg.db 3.7.0<br>pheatmap 1.0.12<br>edgeR 3.24.3<br>DESeq2 1.22.2<br>ggplot2 3.3.1<br>RColorBrewer 1.1-2<br>GSVA 1.30.0<br>gplots 3.0.1.1<br>eulerr 6.0.<br>Further RNA sequencing analysis performed with:<br>Metascape online tool<br>IPA v01-13 (Qiagen)<br><br>Multispectral image analysis:<br>inForm v2.4.8 software (Akoya Bioscience)<br><br>qRT-PCR, cell density and neighbourhood analysis:<br>Prism 7.0.0 (Graphpad)

Excel 2007 v12.0 (Microsoft)
Python 3.8

For manuscripts utilizing custom algorithms or software that are central to the research but not yet described in published literature, software must be made available to editors and reviewers. We strongly encourage code deposition in a community repository (e.g. GitHub). See the Nature Portfolio guidelines for submitting code & software for further information.

Data

Policy information about availability of data

All manuscripts must include a data availability statement. This statement should provide the following information, where applicable:

- Accession codes, unique identifiers, or web links for publicly available datasets
- A description of any restrictions on data availability
- For clinical datasets or third party data, please ensure that the statement adheres to our policy

Data availability:
The RNA-sequencing data generated in this study and raw data for Fig. 6h-j have been deposited in the Zenodo database (https://zenodo.org/record/7016644#.YwSzUhxByN4) DOI: 10.5281/zenodo.7016644.
The data supporting the findings of this study are provided in the Supplementary Information/Source Data file.

Code avaliability:
All codes used in this study are consistent with the "best practice workflow" suggested by the developers of each used tool as decribed in Methods section. RNA-sequencing data were analyzed with the same pipeline, based on described software. Heatmaps and plots were generated with standard parameters for each of the described tools. Codes are deposited in github (github.com/AlessandraCastagna/RNASeq_NatureComm2022)
Mathematical code: the "fully parameterized" model has been deposited in the Biomodels database assigned the identifier MODEL2209160001.
The code used to perform neighbourhood analysis is provided in the Source Data file.

Field-specific reporting

Please select the one below that is the best fit for your research. If you are not sure, read the appropriate sections before making your selection.

☒ Life sciences ☐ Behavioural & social sciences ☐ Ecological, evolutionary & environmental sciences

For a reference copy of the document with all sections, see nature.com/documents/nr-reporting-summary-flat.pdf

Life sciences study design

All studies must disclose on these points even when the disclosure is negative.

Sample size

For in vitro studies, sample size was defined on the basis of past experience with monocytes-derived macrophages. We observed that from 60ml of blood we were able to obtain a range of 50-150 million of monocytes that differenciate into macrophages after one week with M-CSF. We performed a pilot experiment in which we observed that 1 million of macrophages for each experimental condition was sufficient to perform FACS sorting and to obtain RNA with a good quantity and quality to perform the sequencing. Indeed, a single donor was sufficient for one replicate. We used three different donors for three replicates. About fibroblasts, we used a commercial cell line and we plated 450.000 cells for condition. Given that cell type utilized showed low variability and high riproducibility, and our past experience (in particular with primary macrophages cultures), we conclude that a biological triplicate was sufficient to produce statistically significant data. No statistical methods were used to predetermine sample size.
For ex vivo studies, we performed experiments on exemplary samples from nephrectomies and/or biopsies from 11 patients without applying statistical methods other than descriptive observations; for multiplexed immunohistochemistry (mIHC) experiments 2-4 validation experiments per IHC assay were performed on subsequent nearly consecutive sections with identical primary antibodies in automated chromogenic single or duplex immunohistochemical staining experiments, confirming identical staining patterns in comparison with single-channel view after multiplexed immunohistochemistry; for laser captured microdissection experiments we used sections from three different transplanted kidneys at different stages of fibrosis and in control samples, represented by non-fibrotic tissue specimen obtained from tumor-distant areas of kidneys removed for renal cell carcinomas. We decided to use samples in triplicate for RNASeq. No statistical methods were used to predetermine sample size for the fibrotic kidneys, as we used the maximum of suitable cases available from our archive, and the maximum of areas that (1) met the criterias specified in Suppl. Figure 7, and (2) that could be technically dissected by LMD. The sample size for control samples was chosen based on the number of fibrotic kidneys and was matched to cover a similar age and sex distribution.

Data exclusions

Technical failures were excluded. For experiments with human monocytes-derived macrophages samples with eritrocytes or lymphocytes contamination (inefficient Lympholyte or Percoll gradient) were excluded (as detailed in materials and methods section).
During sample processing for laser micro dissection (LMD) of human paraffin-embedded archival material, samples with insufficient RNA amount/quality were excluded (details in materials and methods section)

Replication

Experiments were repeated at least three times with reproducible results. The dataset for RNA Sequencing in vitro study was composed by three independent replicates.
Technical replicates of the RNASeq experiments after LMD were performed to control analytical quality. Due to the limited amount of RNS yield after LMD from paraffin section, we could not perform biological replicates for the materials from archival paraffin-embedded samples.

Randomization

For in vitro experiments, all stimulation with cytokines, hypoxia treatment and co-culture were performed simoultaneously (4h or 24h). Macrophages from each healthy donor were divided randomly from the starting pool and in the same way for all the replicates.
Ex vivo experiments of LMD do not require randomization due to the experimental design.

Blinding

For this type of study blinding was not applicable since most of the experiments were made by a single researcher from the beginning to the end. However samples were prepared, treated and analyzed by the same standard procedure. Moreover, RNA-sequencing, qRT-PCR and IHC are techniques in which blinding of researcher is not relevant thanks to the objective nature of the data produced.

Reporting for specific materials, systems and methods

We require information from authors about some types of materials, experimental systems and methods used in many studies. Here, indicate whether each material, system or method listed is relevant to your study. If you are not sure if a list item applies to your research, read the appropriate section before selecting a response.

Materials & experimental systems

n/a	Involved in the study	
☐	☒	Antibodies
☐	☒	Eukaryotic cell lines
☒	☐	Palaeontology and archaeology
☒	☐	Animals and other organisms
☐	☒	Human research participants
☒	☐	Clinical data
☒	☐	Dual use research of concern

Methods

n/a	Involved in the study	
☒	☐	ChIP-seq
☐	☒	Flow cytometry
☒	☐	MRI-based neuroimaging

Antibodies

Antibodies used

For flow cytometry the following antibodies were used:
CD45 APC-H7 mouse anti human clone 2D1 (RUO), cat. number 560178 (BD)
Live/Dead - Zombie Aqua Fixable Viability kit, cat. number 423101 (BioLegend)

For consecutive IHC staining the following primary antibodies were used:
anti-CD4 (clone SP35; Zytomed Systems, 1:50, #503-3354), anti-CD8 (clone C8/144B; Agilent/Dako, 1:350, #M7103), anti-CD20 (clone L26; Dako, 1:500, #M0755), anti-CD68 (clone PG-M1; Dako, 1:1000, #M0876), anti-CD206 (clone 5C11; Bio-Rad, 1:1500, #MCA5552Z), anti-FAP (clone D8; Vitatex, #MABS1001, 1:1500), polyclonal anti-MS4A4A (Sigma Life Science, 1:200, #HPA029323), and anti-SV40 (clone MRQ-4; Cell Marque, 1:750, #351M-16).

Validation

For flow cytometry, we choosed the CD45 APC-H7 mouse anti human antibody; The 2D1 monoclonal antibody recognizes an epitope on all forms of CD45, a tyrosine phosphatase. We checked manifacture usage and validation (Hermiston ML, Xu Z, Weiss A. CD45: a critical regulator of signaling thresholds in immune cells. Annu Rev Immunol. 2003; 21:107-137. (Biology);Knapp W. W. Knapp .. et al., ed. Leucocyte typing IV : white cell differentiation antigens. Oxford New York: Oxford University Press; 1989:1-1182. (Biology);Loken MR, Brosnan JM, Bach BA, Ault KA. Establishing optimal lymphocyte gates for immunophenotyping by flow cytometry. Cytometry. 1990; 11(4):453-459.(Biology)). For Live/Dead validation see references: Vom Berg J, et al. 2013. J Exp Med. 210:2803. PubMed and Radovanovic I, et al. 2014. J Immunol. 193:1290. PubMed .

All antibodies used in the multiplexing setting are well established in the clinical setting, and passed quality control/certification for diagnostic use. Broadly accepted control tissues with known distribution of the markers were used as positive controls: tonsil for CD20, CD4, CD8, CD68, MS4A4A. Polyoma virus- infected cells from CNS lesion (progressive multifocal leukencphalopathy/PML) was used as a positive control for SV-40 staining. FAP staining was controlled by staining of cancer-associated fibroblasts in a case of breast cancer with extensive desmoplastic tumor stroma. In addition, FAP, CD206, CD68, and MS4A4A staining was performed on paraffin-embedded cell pellets obtained under appropriate cell culture conditions to induce M2-like alternative activation (in macrophages) and myofibroblast activation (in fibroblasts).
The multiplex immunohistochemistry protocols were validated by varying the order of staining and the labeling with different OPAL-fluorophores, and by comparing single-channel staining results with corresponding staining patterns in DAB-based single staining.
We checked manufacture usage and validation provided by web sites: anti-CD4 (https://www.zytomed-systems.com/), anti-CD8 (Mason DY, Cordell JL, Gaulard P, Tse AGD, Brown MH. Immunohistological detection of human cytotoxic/suppressor T cells using antibodies to a CD8 peptide sequence. J Clin Pathol 1992:45:1084-8.; Nuckols JD, Shea CR, Horenstein MG, Burchette JL, Prieto VG. Quantitation of intraepidermal T-cell subsets in formalin-fixed, paraffin-embedded tissue helps in the diagnosis of mycosis fungoides. J Cutan Pathol 1999;26:169-75.; Yamagata K, Tanaka M, Kudo H. A quantitative immunohistochemical evaluation of inflammatory cells at the affected and unaffected sites of inflammatory bowel disease. J Gastroenterol 1998;13:801-8.) anti-CD20 (Blakolmer K, Vesely M, Kummer JA, Jurecka W, Mannhalter C, Chott A. Immunoreactivity of B-cell markers (CD79a, L26) in rare cases of extranodal cytotoxic peripheral T- (NK/T-) cell lymphomas. Mod Pathol 2000;13:766-72.; Tedder TF, Engel P. CD20: a regulator of cell-cycle progression of B lymphocytes. Immunology Today 1994;15:450-4.) anti-CD68 (Falini B, Flenghi L, Pileri S, Gambacorta M, Bigerna B, Durkop H, et al. PG-M1: A new monoclonal antibody directed against a fixative-resistant epitope on the macrophagerestricted form of the CD68 molecule. Am J Pathol 1993;142:1359-72.; Cordell JL, Falini B, Flenghi L, Jones DB, Pileri S, Radzun HJ, et al. M15. CD68 cluster workshop report. In: Knapp W, Dörken B, Gilks WR, Rieber EP, Schmidt RE, Stein H, et al., editors. Leukocyte typing IV. White cell differentiation antigens. Proceedings of the 4th International Workshop and Conference; 1989 Feb 21-25; Vienna, Austria. Oxford, New York, Tokyo: Oxford University Press; 1989. p. 925-7), anti-CD206(Komohara, Y. et al. (2016) Contribution of Macrophage Polarization to Metabolic Diseases.J Atheroscler Thromb. 23 (1): 10-7; Zúñiga-Castillo, M. et al. (2018) High density of M2-macrophages in acral lentiginous melanoma compared to superficial spreading melanoma. Histopathology. 72 (7): 1189-1198.), anti-FAP (https://vitatex.com/products/mabd8-against-seprase-fap) polyclonal anti-MS4A4A (Multiomics Analysis Reveals the Prognostic Non-tumor Cell Landscape in Glioblastoma Niches. Zixuan Xiao et al. Frontiers in genetics, 12, 741325-741325 (2021-10-05); Differential expression and regulation of MS4A family members in myeloid cells in physiological and pathological

conditions. Rita Silva-Gomes et al. Journal of leukocyte biology (2021-08-05)), and anti-SV40 (https://www.cellmarque.com/antibodies/CM/2040/SV40_MRQ-4).

Eukaryotic cell lines

Policy information about cell lines

Cell line source(s)	BJ fibroblast cell line (CRL-2522) was purchased from ATCC
Authentication	Cells were authenticated by checking morphology at microscope
Mycoplasma contamination	Cells were not tested for mycoplasma contamination
Commonly misidentified lines (See ICLAC register)	No commonly misidentified cell lines were used in this study

Human research participants

Policy information about studies involving human research participants

Population characteristics	Histological samples:<br>The cases include nephrectomies and/or biopsies from 11 patients both male and female ranging from 30-80 years of age (details are reported in Supplementary Table S5)<br><br>Monocyte-derived macrophages:<br>Healthy blood donors were both male and female ranging from 20-50 years of age.
Recruitment	Histological samples:<br>The cases include three explanted kidneys that lost function due to previous TCMR and/or ABMR, and one nephrectomy specimen surgically removed because of the development of renal cell carcinoma within the transplanted organ (Merveille, O., et al., An automatic framework for fusing information from differently stained consecutive digital whole slide images: A case study in renal histology. Comput Methods Programs Biomed.208:106157 (2021)).As non-fibrotic control samples we used tissue selected as distant as possible from the tumor margin form four tumor nephrectomy specimens. Protocol biopsies and indicated biopsies from kidney transplants were obtained from a previous study (29. Schaadt N. S., et al., Graph-based description of tertiary lymphoid organs at single-cell level. PLoS Comput Biol.16(2): p. e1007385 (2020)), and the clinical follow-up (development of GFR over time) was obtained in the context of the SYSIMIT systems medicine study (www.sysim.it).<br><br>Monocyted-derived macrophages:<br>No specific restrictions for healthy blood donors recruitment
Ethics oversight	Histological samples:<br>The study was approved by the local institutional review board (IRB) the Ethikkommission (Ethics Commission) of Hannover Medical School; approval number #2063-2013 and its amendment #2968-2015. Additional ethical approval was obtained according to the guideline for ERACoSysMed- funded translational projects, including Comité de Protection des Personnes, Est-IV (Ethical Research Committee), Strasbourg, France and the Comitato Etico Indipendente (Independent Ethics Committee) of IRCCS, Milan, Italia. The approvals cover (1) research use of surplus archivial material from nephrectomy and indicated biopsy samples after completion of the diagnostic workup and associated anonymized (non-identifiable) clinical information by a waiver for individual informed consent, and (2) research use of surplus archivial biopsy material and associated pseudonymised clinical information of patients who gave their written informed consent when entering the Hannover Medical School protocol biopsy registry program.<br><br>Monocyted-derived macrophages:<br>Monocytes-derived macrophages differentiation from healthy blood donors was approved by Humanitas Reasearch Hospital Ethical Committee.

Note that full information on the approval of the study protocol must also be provided in the manuscript.

Flow Cytometry

Plots

Confirm that:

☒ The axis labels state the marker and fluorochrome used (e.g. CD4-FITC).

☒ The axis scales are clearly visible. Include numbers along axes only for bottom left plot of group (a 'group' is an analysis of identical markers).

☒ All plots are contour plots with outliers or pseudocolor plots.

☒ A numerical value for number of cells or percentage (with statistics) is provided.

Methodology

Sample preparation

Human monocytes were obtained from healthy blood donor buffy coats. Monocytes were isolated by two-step density gradient centrifugations using Lympholyte H (Cederlane) and 46%Percoll (Lonza) followed by incubation of purified cells in RPMI 1640 (Lonza) without serum, for 20 min at RT. Adherent monocytes were washed twice with PBS and then cultured in RPMI medium supplemented with 10% fetal bovine serum (FBS; Lonza), 100 U/mL penicillin/streptomycin (Lonza), and 2 mM L-glutamine (Lonza). Mϕ were obtained by culturing monocytes for 7 days in complete RPMI supplemented with human M-CSF (100ng/ml; Miltenyi). The human dermal BJ fibroblast cell line (CRL-2522; ATCC) was cultivated in high glucose D-MEM (Lonza) 10% FBS, 100 U/mL penicillin/streptomycin, and 2 mM L-glutamine. When cultivated in normoxic conditions, Mϕ and Fb were maintained at 37°C in a humidified incubator settled at 20% O2, 5% CO2 in air, while hypoxic treatment was performed moving cells at 37°C in a humidified incubator with a mixture of 1% O2, 5% CO2 and 94% N2. Mϕ were polarized toward a proinflammatory phenotype (MI) by incubation with 100 ng/ml LPS (Sigma) plus 20 ng/ml IFNγ (R&D Systems) or into an alternative phenotype (MF) by incubation with 20 ng/ml IL-4 (Miltenyi). Resting Mϕ (M0) were left unstimulated for the same period. Fb were stimulated as Mϕ, with cells treated with LPS+IFNγ (FbI), IL-4 (FbF) or left unstimulated (Fb0). Polarizing stimuli and hypoxia were applied simultaneously. For coculture experiments, differentiated Mϕ were replated directly onto adherent Fb (plated 16h before) with a 2:1 ratio. After 24h of coculture in basal conditions (normoxia without stimuli), cells were stimulated as described above, detached, and FACS sorted based on staining with anti-human CD45 (BD Bioscience) to distinguish CD45+ Mϕ from CD45- Fb. Zombie Aqua Fixable Viability kit (BioLegend) was used to exclude dead cells.

Instrument

FACS Aria III cell sorter (BD Bioscience)

Software

BD FACSDiva

Cell population abundance

Macrophages and fibroblasts were discriminated by using CD45 antibody after dead cells exclusion. Positive fraction were represented by macrophages and negative fraction by fibroblasts. Percentages of each fraction was variable but the purity was always >95% for both macrophages and fibroblasts.

Gating strategy

Gating strategy is reported in Figure S1c; firstly doublets were excluded (FSC-H/FSC-A and SSC-A/FSC-A), then we excluded dead cells (positive for L/D staining) and finally we were able to clearly distinguish positive and negative CD45 cells.

☒ Tick this box to confirm that a figure exemplifying the gating strategy is provided in the Supplementary Information.
